# Supplementary material for: Conformational maps of human 20S proteasomes reveal PA28- and immuno-dependent inter-ring crosstalks
Source: Nat Commun. 2020 Dec 1;11:6140. doi: 10.1038/s41467-020-19934-z (PMC7708635; doi:10.1038/s41467-020-19934-z)
Supplement: Supplementary file 1 — Supplementary Information [file 41467_2020_19934_MOESM1_ESM.pdf]

Supplementary Table 1. Sequence coverage and number of peptides obtained upon pepsin digestion of the 20S and PA28 subunits

	20S alone		20S+PA28	
	sequence coverage across time points (%)	number of unique peptides	sequence coverage across time points (%)	number of unique peptides
α1	93.2	52	93.5	44
α2	94.9	60	92.7	45
α3	92	56	81.2	43
α4	92.7	55	72.2	38
α5	93.8	57	85.9	38
α6	93.2	58	89.4	37
α7	94.9	62	92.2	48
β1	96.6	59	93.2	49
β1i	80.9	32	77.4	26
β2	92.3	61	90.6	51
β2i	64.1	21	59	17
β3	89.7	41	80.4	29
β4	94	43	86.6	40
β5	85.3	55	85.3	45
β5i	60.3	19	51	14
β6	91.1	36	75.6	24
β7	78.1	35	77.6	31
	PA28 alone		PA28+20S	
PA28α	93.2	62	71.9	38
PA28β	94.6	69	80.3	39
PA28γ	90.9	99	81.5	53

average 20S alone	average PA28 alone	average 20S complex	average PA28 complex
89.1	92.9	81.4	77.9

Supplementary Table 2. Identity matrix generated by Clustal2.1 showing the percentage of identity between PfPA28 and human PA28α, PA28β and PA28γ.

	PfPA28 | PDBID|	PA28γ | P61289| 2-254	PA28α | Q06323| 1-249	PA28β | Q9UL46| 2-239
PfPA28 | PDBID|	100	34.13	27.82	26.58
PA28γ | P61289| 2-254	34.13	100	40.56	35.17
PA28α | Q06323| 1-249	27.82	40.56	100	49.15
PA28β | Q9UL46| 2-239	26.58	35.17	49.15	100

Supplementary Table 3. Proteolytic activity test (chymotrypsin-like) of the std20S alone and after incubation with PA28γ or PA28αβ.

	std20S			PA28γ			PA28αβ			std20S + PA28γ			std20S + PA28αβ		
Time	std20S chymo. R1	std20S chymo. R2	std20S chymo. R3	PA28γ chymo. R1	PA28γ chymo. R2	PA28γ chymo. R3	PA28αβ chymo. R1	PA28αβ chymo. R2	PA28αβ chymo. R3	std20S + PA28γ chymo. R1	std20S + PA28γ chymo. R2	std20S + PA28γ chymo. R3	std20S + PA28αβ chymo. R1	std20S + PA28αβ chymo. R2	std20S + PA28αβ chymo. R3
00:00:00	177	178	182	180	177	188	182	180	175	182	190	197	180	182	191
00:05:00	159	160	165	158	156	164	160	157	155	185	184	190	182	179	198
00:10:00	164	168	176	149	147	155	147	150	146	221	210	218	224	223	253
00:15:00	191	192	207	145	143	149	144	145	141	287	267	278	296	290	341
00:20:00	235	231	249	143	138	148	143	142	137	383	347	367	392	382	448
00:25:00	282	270	301	138	137	144	138	140	134	489	444	466	490	475	552
00:30:00	333	319	353	139	134	144	137	138	133	595	548	578	603	588	684
00:35:00	382	366	409	135	134	142	136	136	132	711	655	686	704	693	800
00:40:00	430	412	461	134	132	140	135	136	131	816	764	801	811	796	912
00:45:00	482	459	516	132	132	139	132	136	128	920	872	920	909	895	1027
00:50:00	531	502	569	132	130	138	131	135	128	1018	982	1022	1004	995	1134
00:55:00	577	556	621	131	130	138	130	134	127	1127	1089	1135	1100	1091	1236
01:00:00	629	596	671	129	126	134	127	134	127	1226	1194	1238	1186	1181	1337
01:05:00	675	640	719	128	126	134	127	133	125	1324	1293	1344	1283	1275	1432
01:10:00	719	683	769	128	126	134	128	131	125	1430	1392	1445	1371	1365	1528
01:15:00	764	726	816	127	127	133	127	129	123	1518	1491	1547	1451	1451	1615
01:20:00	811	771	863	125	126	132	125	130	122	1613	1593	1646	1542	1540	1710
01:25:00	855	812	909	125	124	131	126	129	120	1709	1692	1744	1622	1622	1799
01:30:00	901	855	957	123	126	131	124	129	120	1797	1792	1841	1705	1704	1889
slope	14144.9143	13494.8571	15428.5714	-419.657143	-263.314286	-353.828571	-436.114286	-189.257143	-370.285714	30322.2857	31219.2	32190.1714	28660.1143	28964.5714	31902.1714
R^2	0.99978436	0.9993114	0.99988813	0.86748193	0.91428571	0.91610239	0.96695353	0.85552561	0.93822394	0.99946123	0.99998768	0.99967496	0.99954606	0.99977246	0.99949225
activity (nmol AMC/min/mL)	3677.67771	3508.66286	4011.42857	-109.110857	-68.4617143	-91.9954286	-113.389714	-49.2068571	-96.2742857	7883.79429	8116.992	8369.44457	7451.62971	7530.78857	8294.56457
Modified Activity	3677.67771	3508.66286	4011.42857	0	0	0	0	0	0	7883.79429	8116.992	8369.44457	7451.62971	7530.78857	8294.56457
20S quantity (µg)	0.05	0.05	0.05	0	0	0	0	0	0	0.05	0.05	0.05	0.05	0.05	0.05
specific proteasome activity (nmol AMC/min/µg P	73553.5543	70173.2571	80228.5714	0	0	0	0	0	0	157675.886	162339.84	167388.891	149032.594	150615.771	165891.291
Average	74651.7943			0			0			160007.863			149824.183		
stdev	5116.82858			0			0			3297.9137			1119.47529		
Normalized Activity	98.53	94.00	107.47	0.00	0.00	0.00	0.00	0.00	0.00	211.22	217.46	224.23	199.64	201.76	222.22
Average	100.00			0.00			0.00			217.63			207.87		
stdev	6.85			0.00			0.00			6.51			12.47		
p-value (t-test)										0.0007			0.0013		

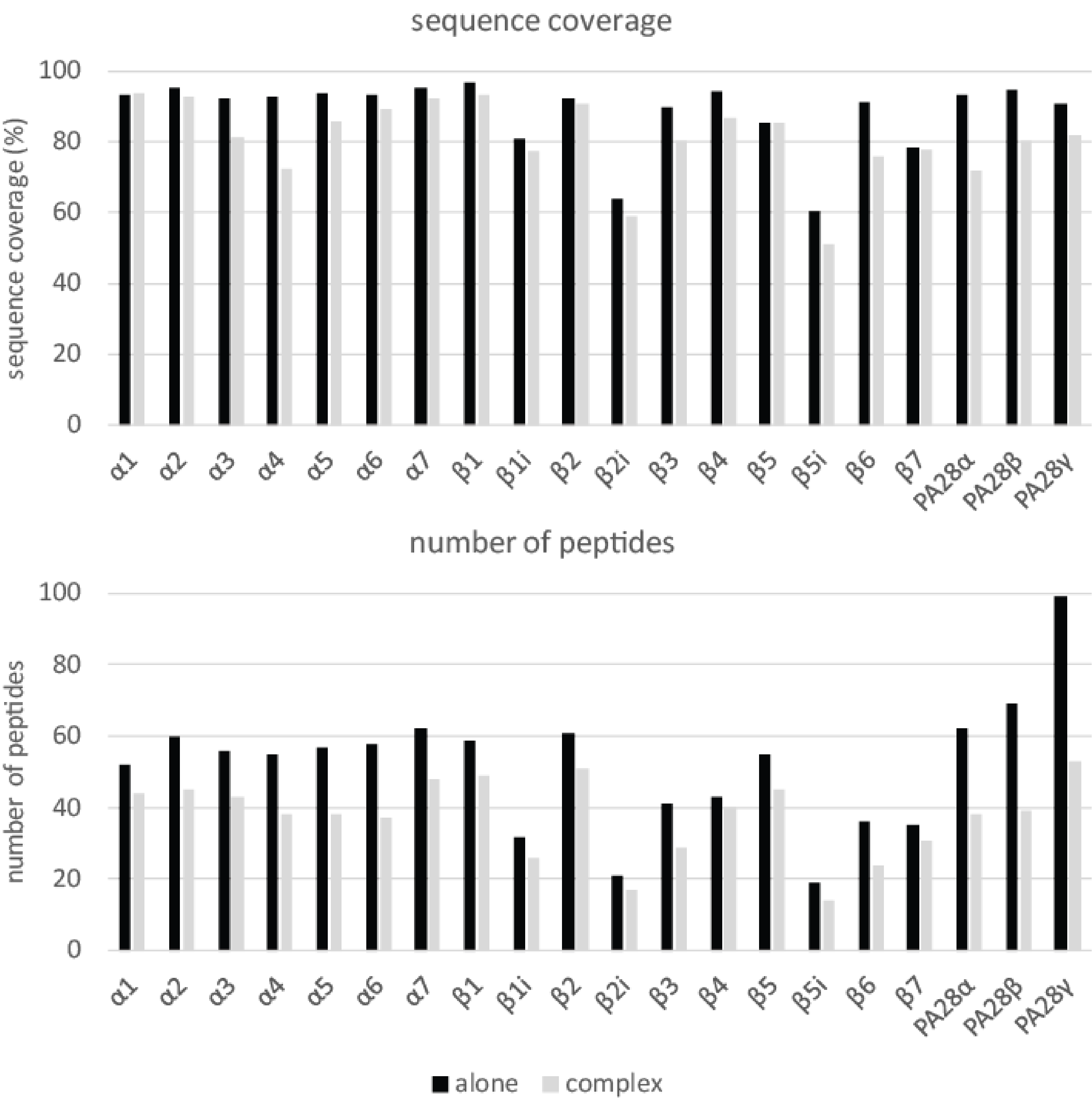

Supplementary Figure 1. Sequence coverage and number of peptides obtained upon pepsin digestion of the 20S and PA28 subunits. The bars present the sequence coverage (top) and number of peptides (bottom) obtained for each subunit in the samples with the proteins alone (black) or in complex (grey).

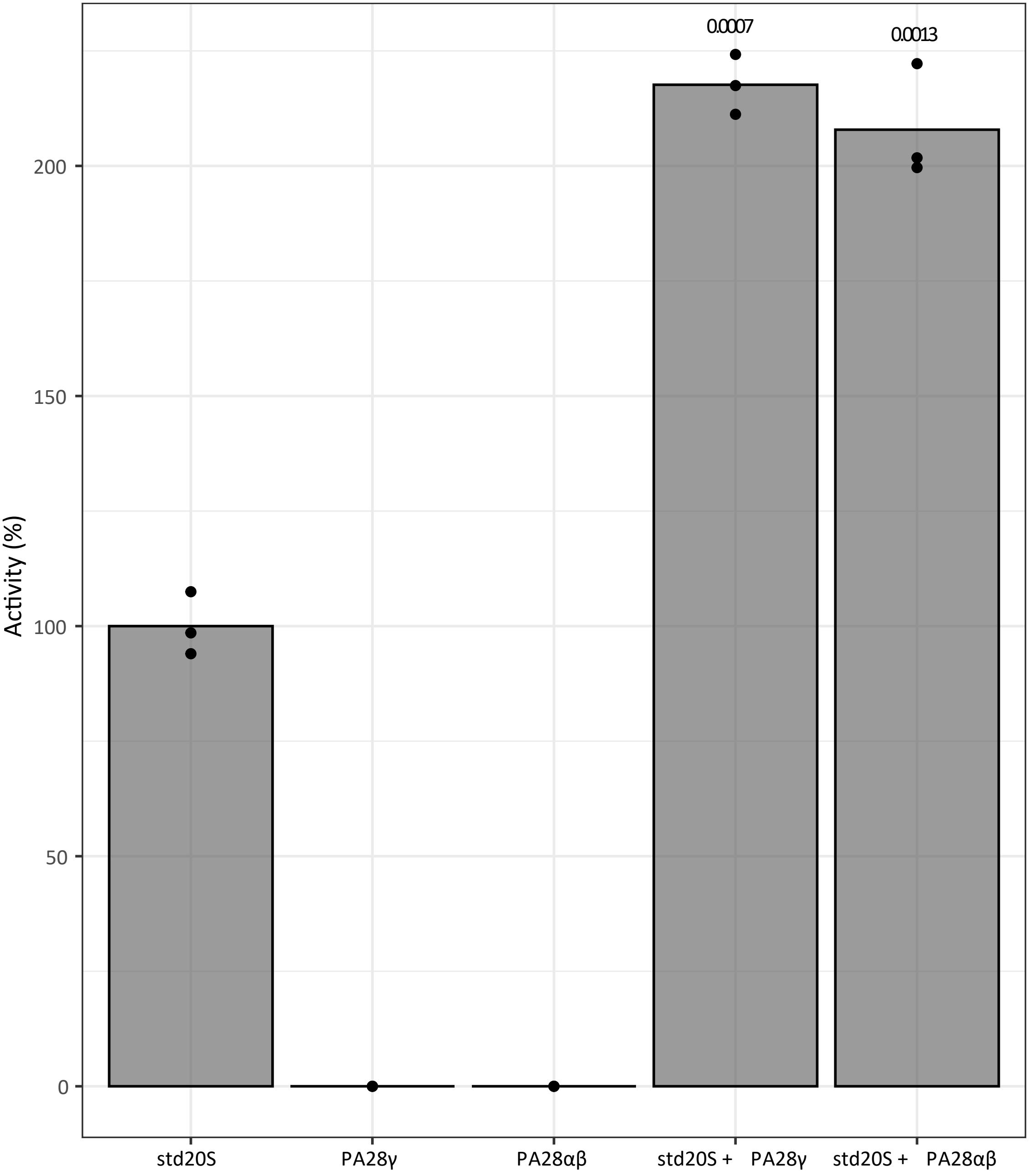

Supplementary Figure 2. Proteolytic activity test (chymotrypsin-like) of the std20S alone and after incubation with PA28γ or PA28αβ. Bars are the mean of protease activity relative to control (std20S) in 3 independent experiments (points). The P-values of a paired two-sided t-test against the std20S alone are presented above the corresponding bars.
