# Supplementary material for: Conformational maps of human 20S proteasomes reveal PA28- and immuno-dependent inter-ring crosstalks
Source: Nat Commun. 2020 Dec 1;11:6140. doi: 10.1038/s41467-020-19934-z (PMC7708635; doi:10.1038/s41467-020-19934-z)
Supplement: Supplementary file 3 — Dataset 1 [file 41467_2020_19934_MOESM3_ESM.pdf]

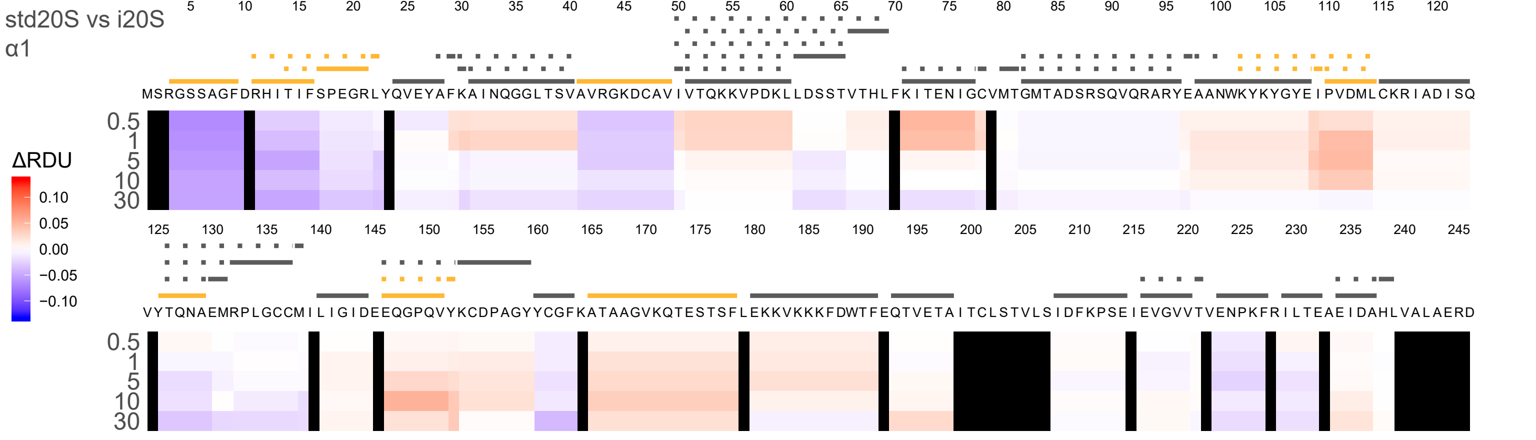
std20S vs i20S
α1
ΔRDU
0.10
0.05
0.00
−0.05
−0.10
MSRGSSAGFDRHITIFSPEGRLYQVEYAFKAINQGGLTSVAVRGKDCAVIVTQKKVPDKLLDSSTVTHLFKITENIGCVMTGMTADSRSQVQRARYEAANWKYKYGYEIPVDMLCKRIADISQ
VYTQNAEMRPLGCCMILIGIDEEQGPQVYKCDPAGYYCGFKATAAGVKQTESTSFLEKKVKKKFDWTFEQTVETAITCLSTVLSIDFKPSEIEVGVVTVENPKFRILTEAEIDAHLVALAERD
0.5
1
5
10
30

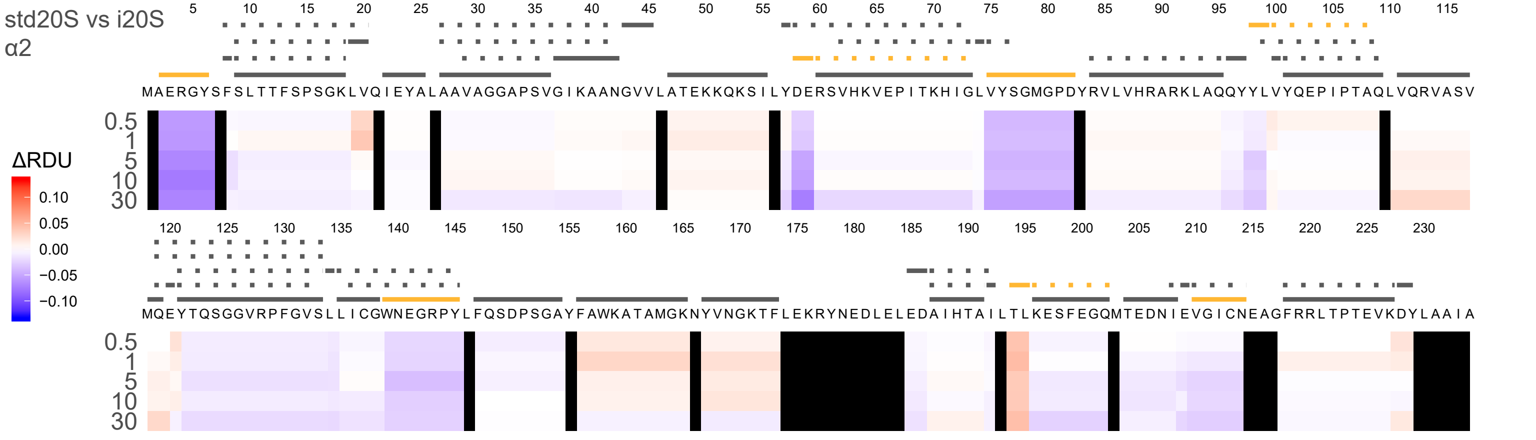

std20S vs i20S
α2
ΔRDU
0.10
0.05
0.00
−0.05
−0.10
0.5
1
5
10
30
MAERGYSFSLTTFSPSGKLVQIEYALAAVAGGAPSVGIKAANGVVLATEKKQKSILYDERSVHKVEPITKHIGLVYSGMGPDYRVLVHRARKLAQQYYLVYQEPIPTAQLVQRVASV
MQEYTQSGGVRPFGVSLLICGWNEGRPYLFQSDPSGAYFAWKATAMGKNYVNGKTFLEKRYNEDLELEDAIHTAILTLKESFEGQMTEDNIEVGICNEAGFRRLTPTEVKDYLAAIA

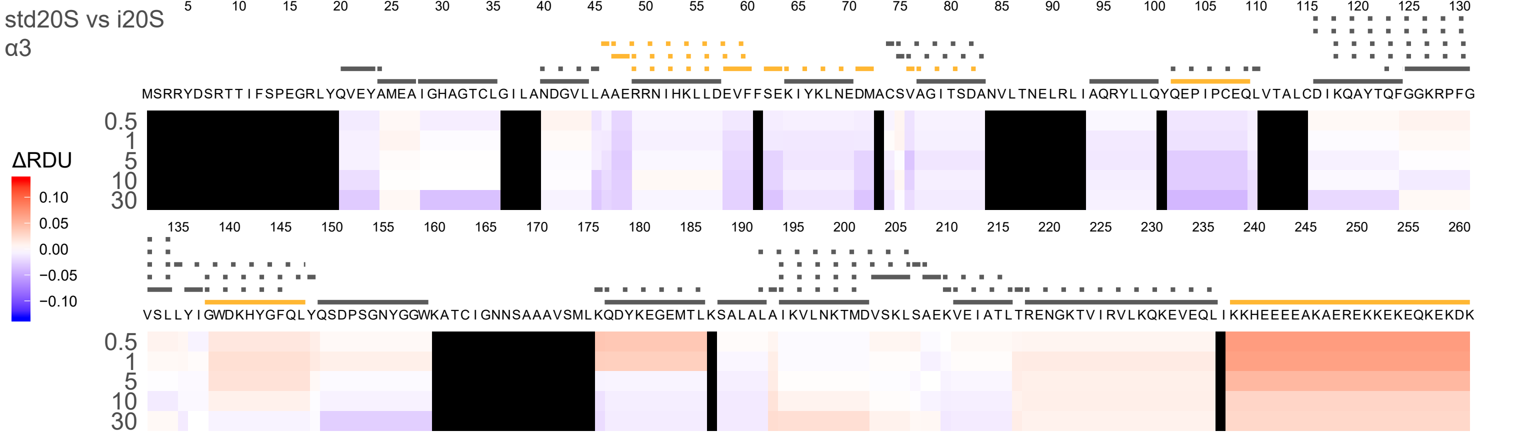

std20S vs i20S
α3
ΔRDU
0.10
0.05
0.00
−0.05
−0.10
MSRRYDSRTTIFSPEGRLYQVEYAMEAIGHAGTCLGILANDGVLLAAERRNIHKLLDEVFFSEKIYKLNEDMACSVAGITSDANVLTNELRLIAQRYLLQYQEPIPCEQLVTALCDIKQAYTQFGGKRPFG
VSLLYIGWDKHYGFQLYQSDPSGNYGGWKATCIGNNSAAAVSMLKQDYKEGEMTLKSALALAIKVLNKTMDVSKLSAEKVEIATLTRENGKTVIRVLKQKEVEQLIKKHEEEEAKAEREKKEKEQKEKDK
0.5
1
5
10
30

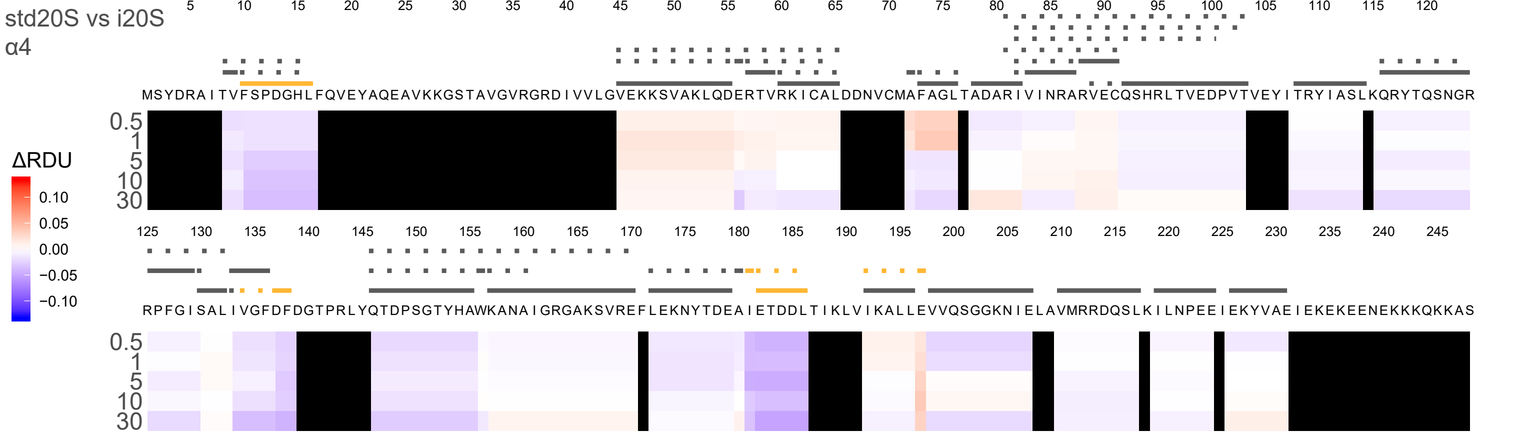

std20S vs i20S
α4
ΔRDU
0.10
0.05
0.00
−0.05
−0.10
0.5
1
5
10
30
MSYDRAITVFSPDGHLFQVEYAQEAVKKGSTAVGVRGRDIVVLGVEKKSVAKLQDERTVRKICALDDNVCMAFAGLTADARIVINRARVECQSHRLTVEDPVTVEYITRYIASLKQRYTQSNGR
RPFGISALIVGFDFDGTPRLYQTDPSGTYHAWKANAIGRGAKSVREFLEKNYTDEAIETDDLTIKLVIKALLEVVQSGGKNIELAVMRRDQSLKILNPEEIEKYVAEIEKEKEENEKKKQKKAS

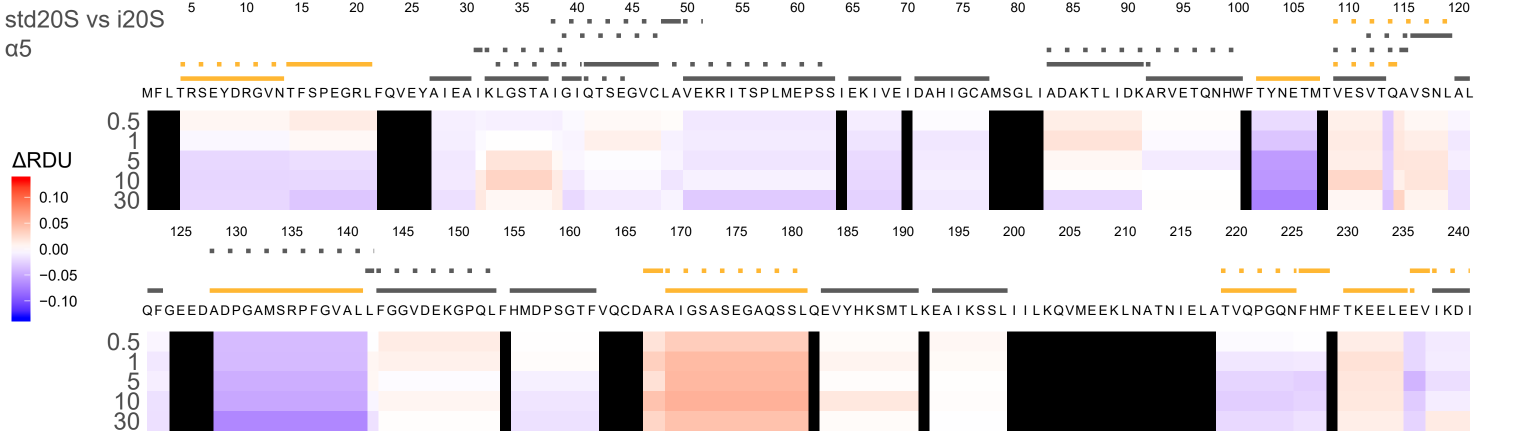
std20S vs i20S
α5
ΔRDU
0.10
0.05
0.00
−0.05
−0.10
0.5
1
5
10
30
5 10 15 20 25 30 35 40 45 50 55 60 65 70 75 80 85 90 95 100 105 110 115 120
MFLTRSEYDRGVNTFSPEGRLFQVEYAIEAIKLGSTAIGIQTSEGVCLAVEKRITSPLMEPSSIEKIVEIDAHIGCAMSGLIADAKTLIDKARVETQNHWFTYNETMTVESVTQAVSNLAL
125 130 135 140 145 150 155 160 165 170 175 180 185 190 195 200 205 210 215 220 225 230 235 240
QFGEEDADPGAMSRPFGVALLFGGVDEKGPQLFHMDPSGTFVQCDARAIGSASEGAQSSLQEVYHKSMTLKEAIKSSLIIILKQVMEEKLNATNIELATVQPGQNFHMFTKEELEEVIKDI

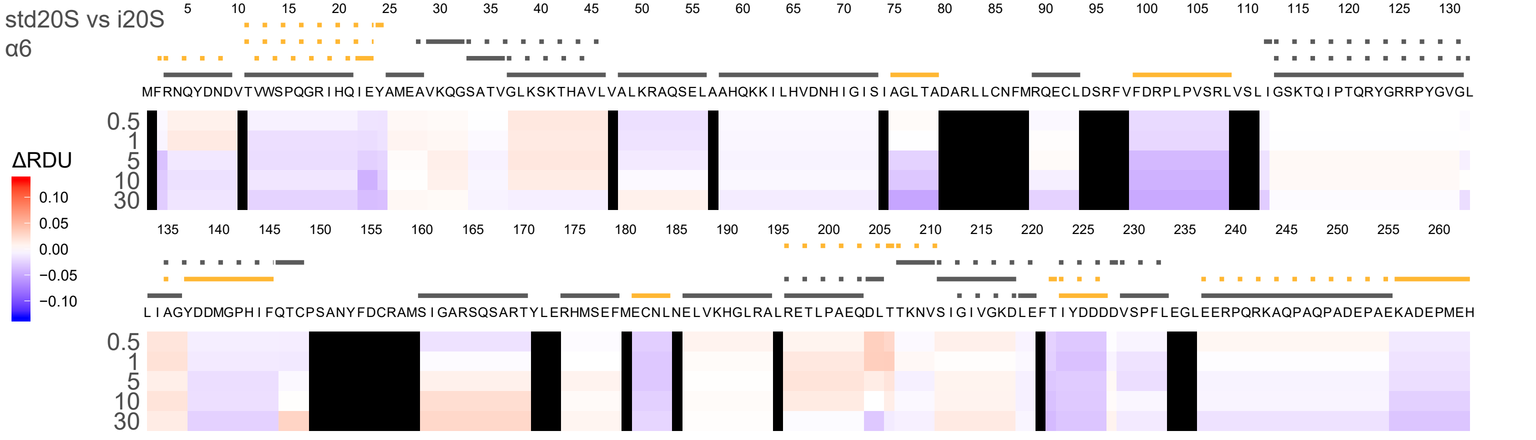

std20S vs i20S
α6
ΔRDU
0.10
0.05
0.00
−0.05
−0.10
MFRNQYDNDVTVWSPQGRIHQIEYAMEAVKQGSATVGLKSKTHAVLVALKRAQSELAAHQKKILHVDNHIGISIAGLTADARLLCNFMRQECLDSRFVFDRPLPVSRLVSLIGSKTQIPTQRYGRRPYGVGL
LIAGYDDMGPHIFQTCPSANYFDCRAMSIGARSQSARTYLERHMSEFMECNLNELVKHGLRALRETLPAEQDLTTKNVSIGIVGKDLEFTIYDDDDVSPFLEGLEERPQRKAQPAQPADEPAEKADEPMEH
0.5
1
5
10
30

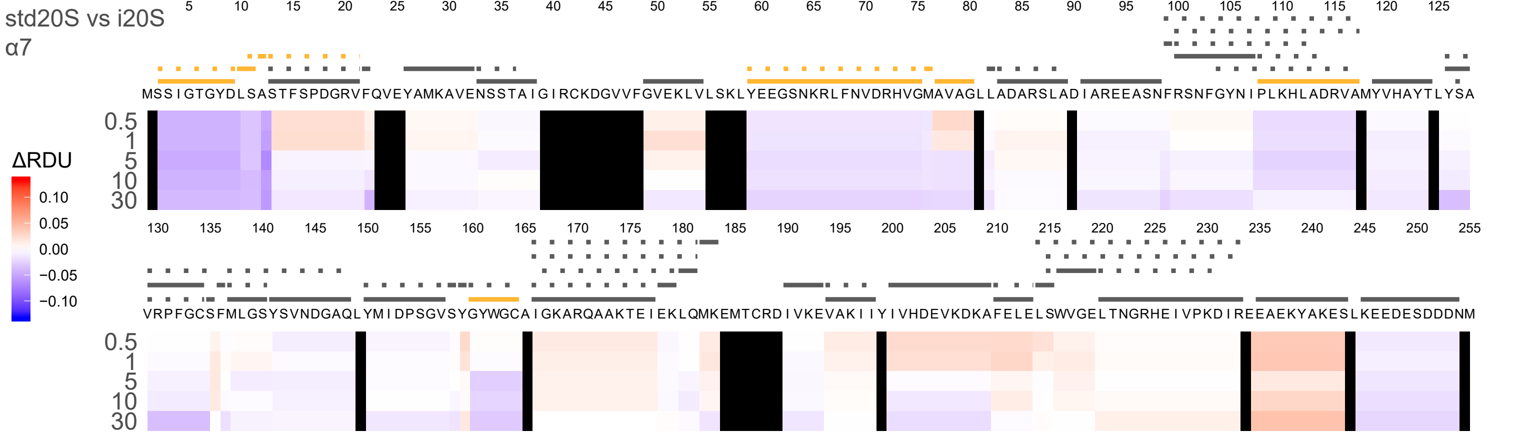

std20S vs i20S
α7
ΔRDU
0.10
0.05
0.00
−0.05
−0.10
MSSIGTGYDLSASTFSPDGRVFQVEYAMKAVENSSTAIGIRCKDGVVFGVEKLVLSKLYEEGSNKRLFNVDRHVGMAVAGLLADARSLADIAREEASNFRSNFGYNIPLKHLADRVAMYVHAYTLYSA
VRPFGCSFMLGSYSVNDGAQLYMIDPSGVSYGYWGCAIGKARQAAKTEIEKLQMKEMTCRDIVKEVAKIIYIVHDEVKDKAFELELSWVGELTNGRHEIVPKDIREEAEKYAKESLKEEDESDDDNM
0.5
1
5
10
30

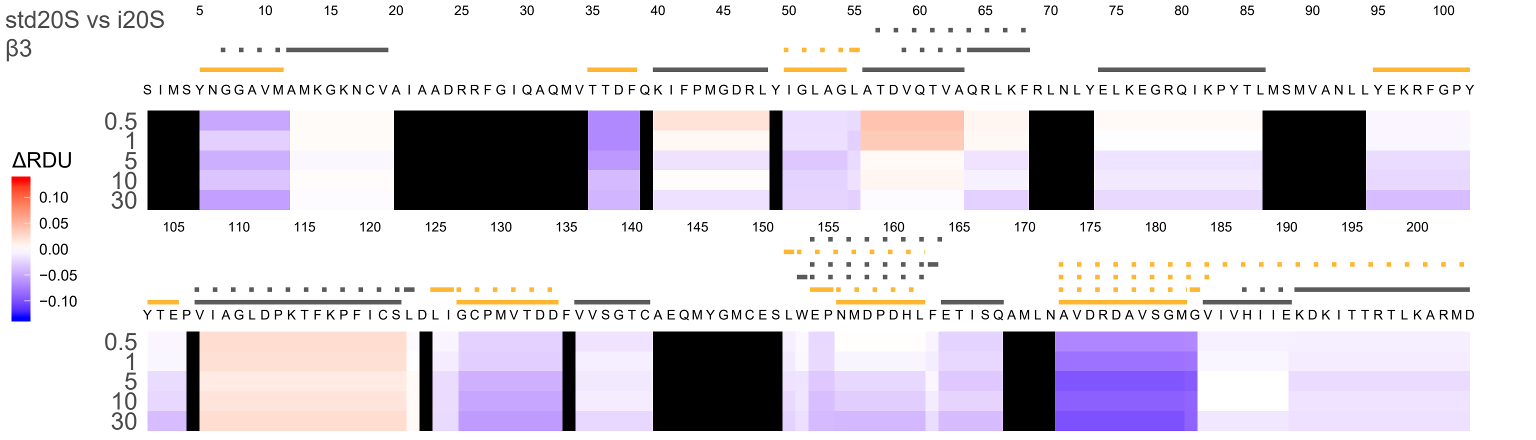
std20S vs i20S
β3
ΔRDU
0.10
0.05
0.00
−0.05
−0.10
0.5
1
5
10
30
S I M S Y N G G A V M A M K G K N C V A I A A D R R F G I Q A Q M V T T D F Q K I F P M G D R L Y I G L A G L A T D V Q T V A Q R L K F R L N L Y E L K E G R Q I K P Y T L M S M V A N L L Y E K R F G P Y
Y T E P V I A G L D P K T F K P F I C S L D L I G C P M V T D D F V V S G T C A E Q M Y G M C E S L W E P N M D P D H L F E T I S Q A M L N A V D R D A V S G M G V I V H I I E K D K I T T R T L K A R M D

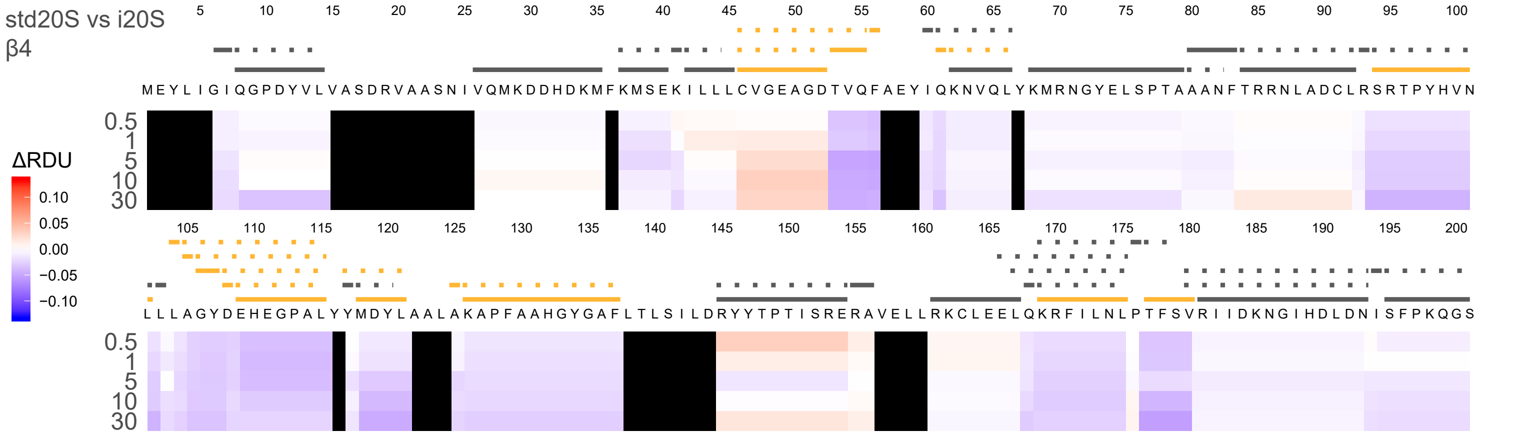

std20S vs i20S
β4
ΔRDU
0.10
0.05
0.00
−0.05
−0.10
5 10 15 20 25 30 35 40 45 50 55 60 65 70 75 80 85 90 95 100
MEYLIGIQGPDYVLVASDRVAASNIVQMKDDHDKMFKMSEKILLLCVGEAGDTVQFAEYIQKNVQLYKMRNGYELSPTAAANFTRRNLADCLRSRTPYHVN
0.5
1
5
10
30
105 110 115 120 125 130 135 140 145 150 155 160 165 170 175 180 185 190 195 200
LLLAGYDEHEGPALYYMDYLAALAKAPFAAHGYGAFLTLSILDRYYTPTISRERAVELLRKCLEELQKRFILNLPTFSVRIIDKNGIHDLDNISFPKQGS
0.5
1
5
10
30

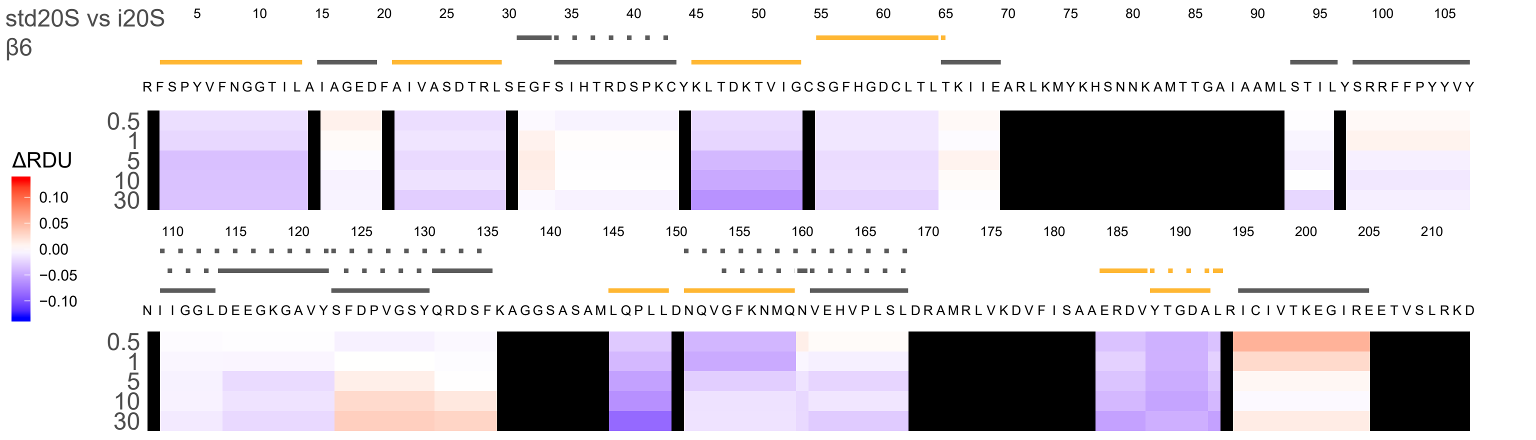

std20S vs i20S
β6
ΔRDU
0.10
0.05
0.00
−0.05
−0.10
0.5
1
5
10
30
RFSPYVFNGGTILAIAGEDFAIVASDTRLSEGFSIHTRDSPKCYKLTDKTVIGCSGFHGDCLTLTKIIEARLKMYKHSNNKAMTTGAIAAMLSTILYSRRFFPYYVY
NIIGGLDEEGKGAVYSFDPVGSYQRDSFKAGGSASAMLQPLLDNQVGFKNMQNVEHVPLSLDRAMRLVKDVFISAAERDVYTGDALRICIVTKEGIREETVSLRKD

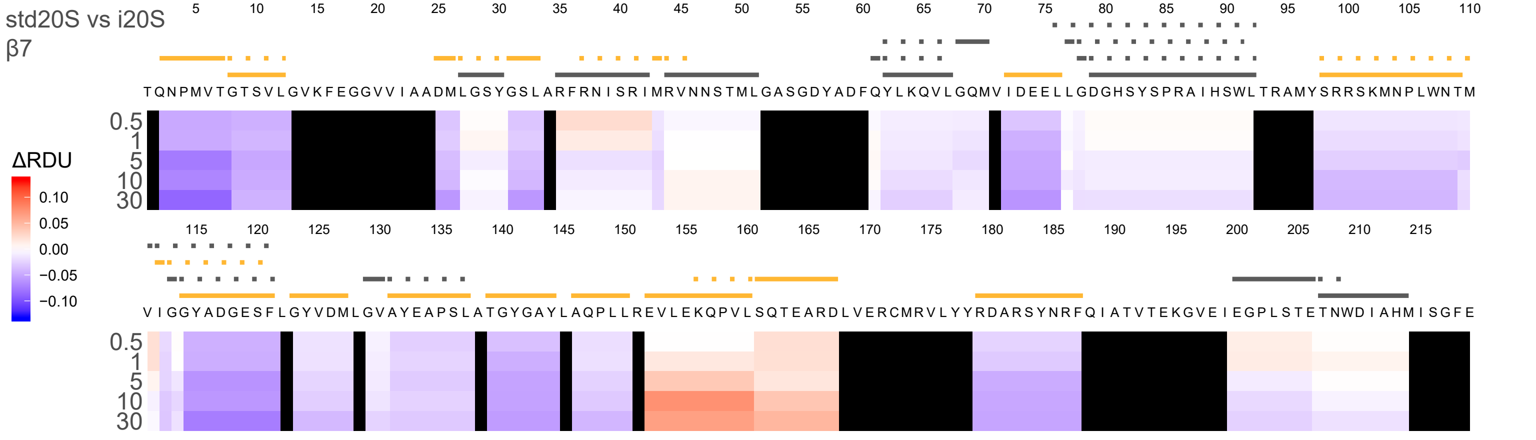
std20S vs i20S
β7
ΔRDU
0.10
0.05
0.00
−0.05
−0.10
5 10 15 20 25 30 35 40 45 50 55 60 65 70 75 80 85 90 95 100 105 110
T Q N P M V T G T S V L G V K F E G G V V I A A D M L G S Y G S L A R F R N I S R I M R V N N S T M L G A S G D Y A D F Q Y L K Q V L G Q M V I D E E L L G D G H S Y S P R A I H S W L T R A M Y S R R S K M N P L W N T M
0.5
1
5
10
30
115 120 125 130 135 140 145 150 155 160 165 170 175 180 185 190 195 200 205 210 215
V I G G Y A D G E S F L G Y V D M L G V A Y E A P S L A T G Y G A Y L A Q P L L R E V L E K Q P V L S Q T E A R D L V E R C M R V L Y Y R D A R S Y N R F Q I A T V T E K G V E I E G P L S T E T N W D I A H M I S G F E
0.5
1
5
10
30
