# Supplementary figures and images for: Conformational maps of human 20S proteasomes reveal PA28- and immuno-dependent inter-ring crosstalks
Source: Nat Commun. 2020 Dec 1;11:6140. doi: 10.1038/s41467-020-19934-z (PMC7708635; doi:10.1038/s41467-020-19934-z)

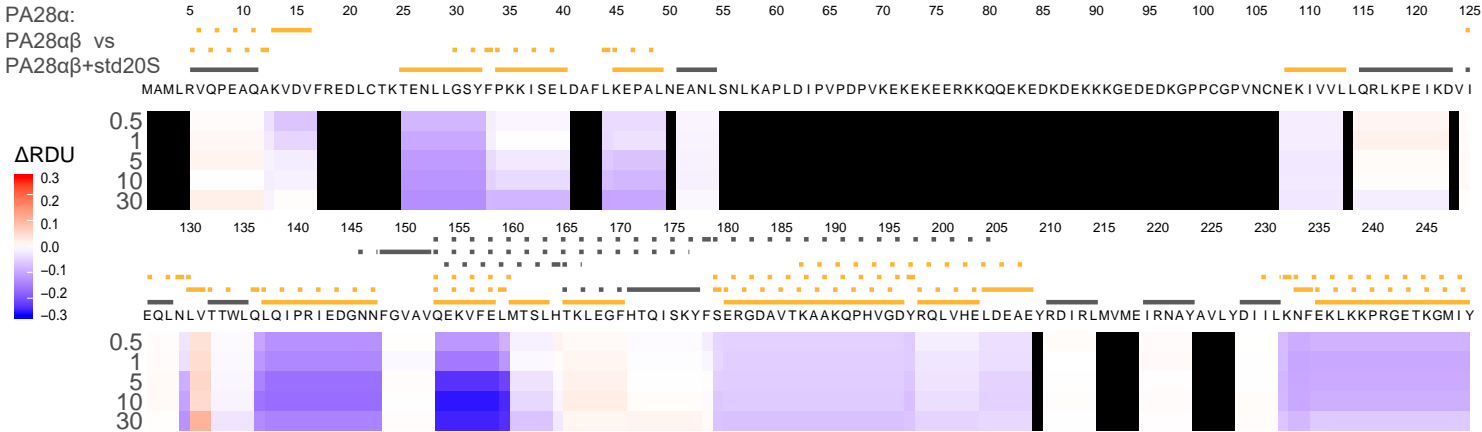

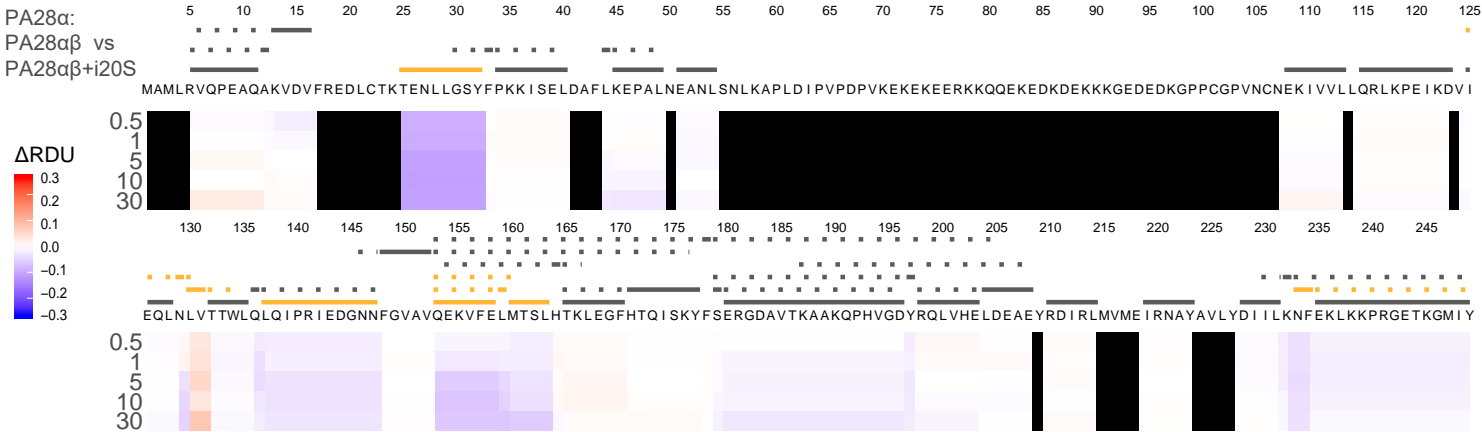

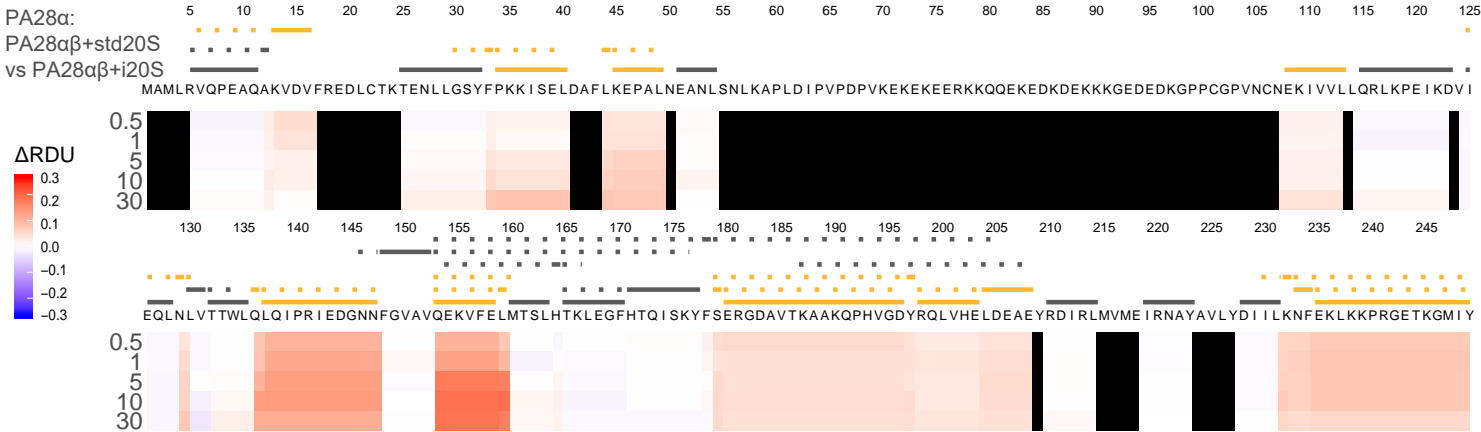

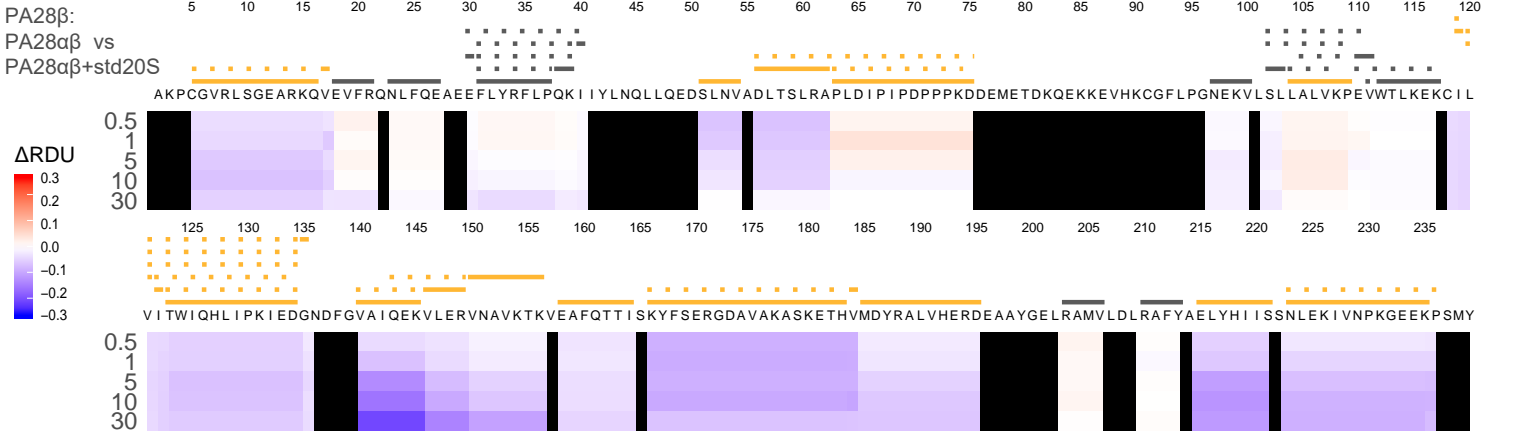

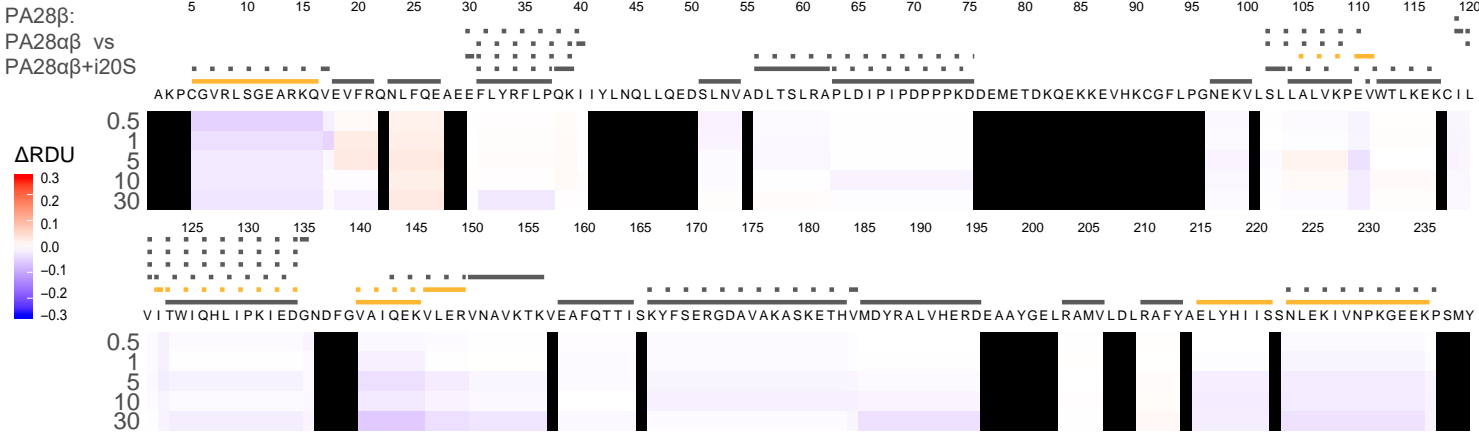

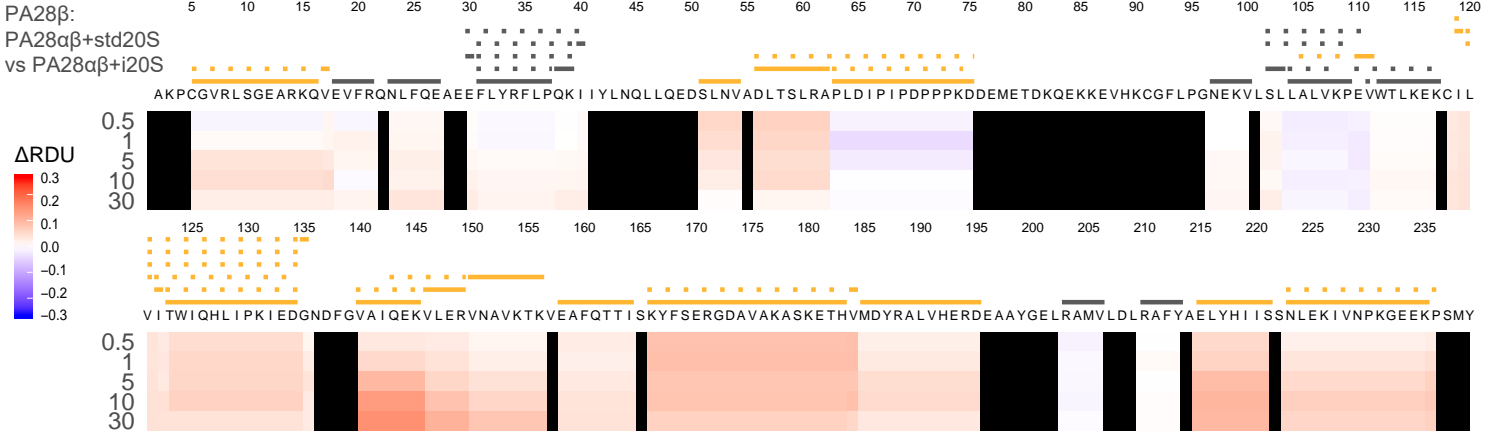

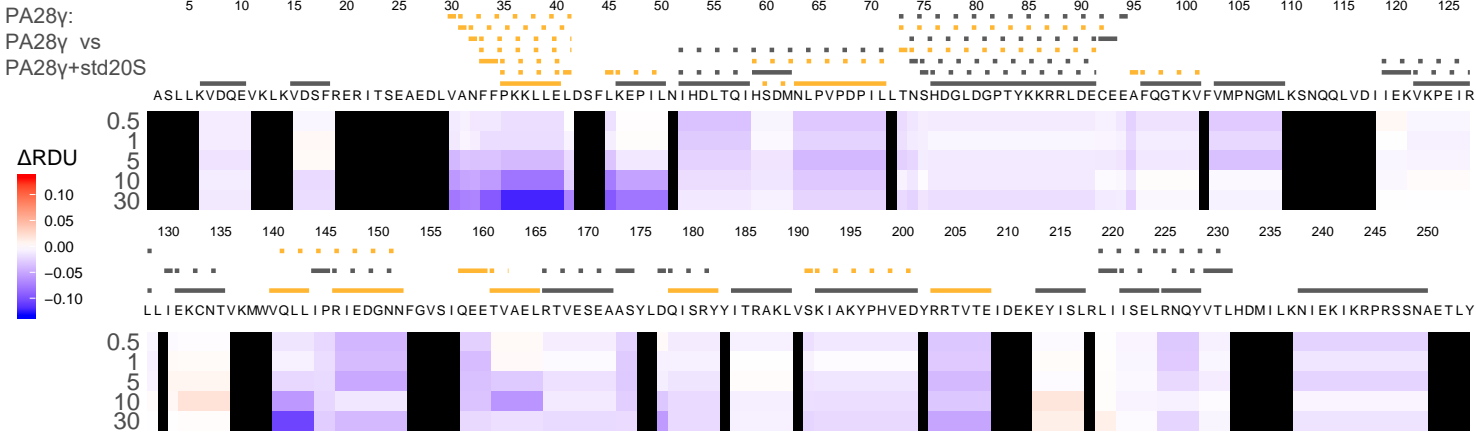

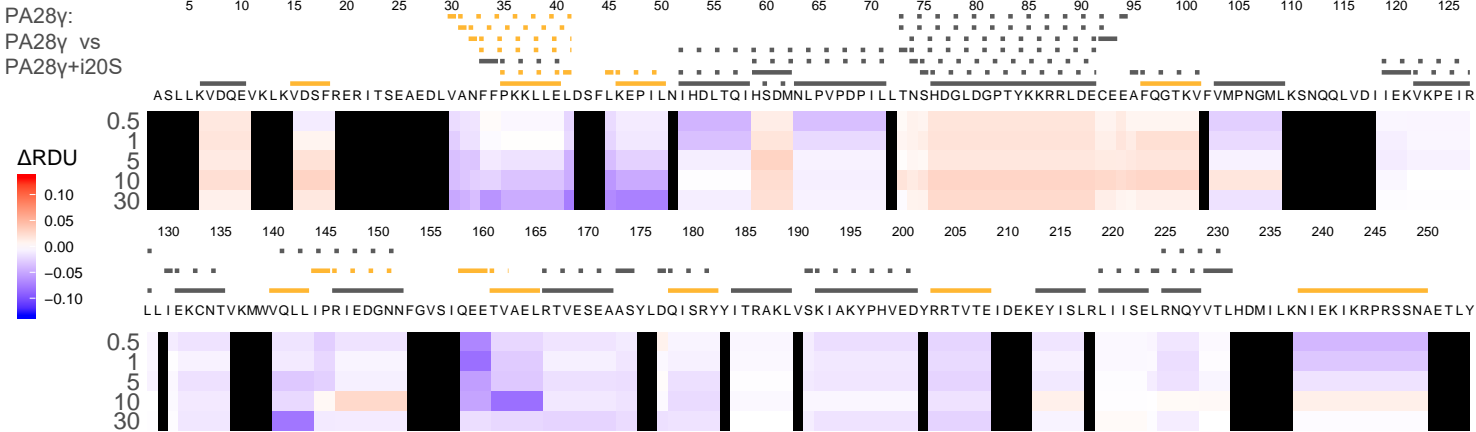

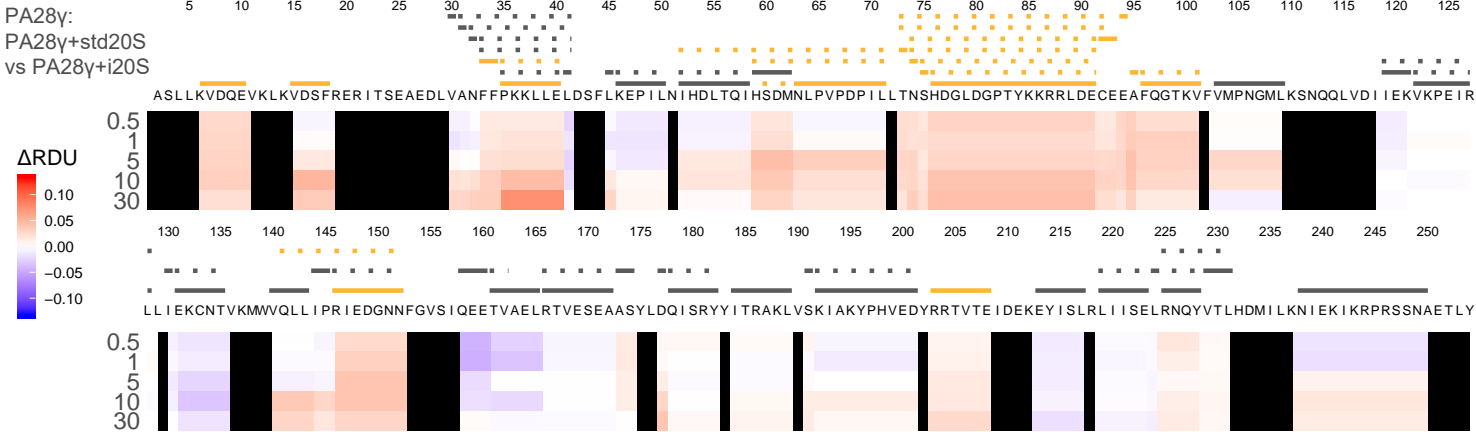

Supplement: Supplementary file 4 — Dataset 2 [file 41467_2020_19934_MOESM4_ESM.pdf]
