# Supplementary material for: Conformational maps of human 20S proteasomes reveal PA28- and immuno-dependent inter-ring crosstalks
Source: Nat Commun. 2020 Dec 1;11:6140. doi: 10.1038/s41467-020-19934-z (PMC7708635; doi:10.1038/s41467-020-19934-z)
Supplement: Supplementary file 5 — Dataset 3 [file 41467_2020_19934_MOESM5_ESM.pdf]

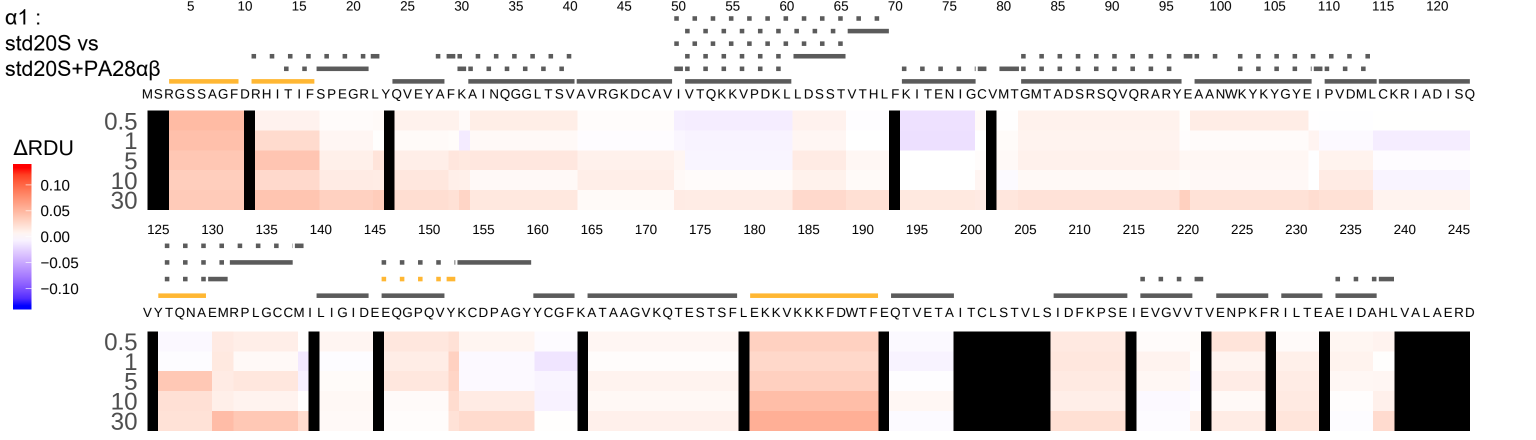

α1 :
std20S vs
std20S+PA28αβ
ΔRDU
0.10
0.05
0.00
−0.05
−0.10
0.5
1
5
10
30
MSRGSSAGFDRHITIFSPEGRLYQVEYAFKAINQGGLTSVAVRGKDCAVIVTQKKVPDKLLDSSTVTHLFKITENIGCVMTGMTADSRSQVQRARYEAANWKYKYGYEIPVDMLCKRIADISQ
VYTQNAEMRPLGCCMILIGIDEEQGPQVYKCDPAGYYCGFKATAAGVKQTESTSFLEKKVKKKFDWTFEQTVETAITCLSTVLSIDFKPSEIEVGVVTVENPKFRILTEAEIDAHLVALAERD

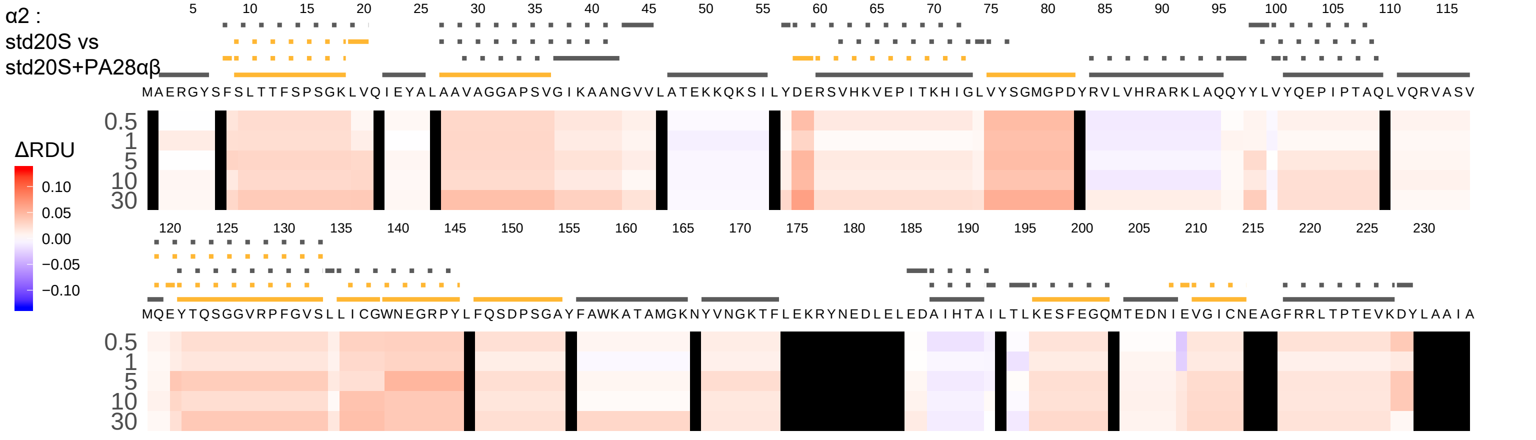

α2 :
std20S vs
std20S+PA28αβ
ΔRDU
0.10
0.05
0.00
−0.05
−0.10
MAERGYSFSLTTFSPSGKLVQIEYALAAVAGGAPSVGIKAANGVVLATEKKQKSILYDERSVHKVEPITKHIGLVYSGMGPDYRVLVHRARKLAQQYYLVYQEPIPTAQLVQRVASV
MQEYTQSGGVRPFGVSLLICGWNEGRPYLFQSDPSGAYFAWKATAMGKNYVNGKTFLEKRYNEDLELEDAIHTAILTLKESFEGQMTEDNIEVGICNEAGFRRLTPTEVKDYLAAIA

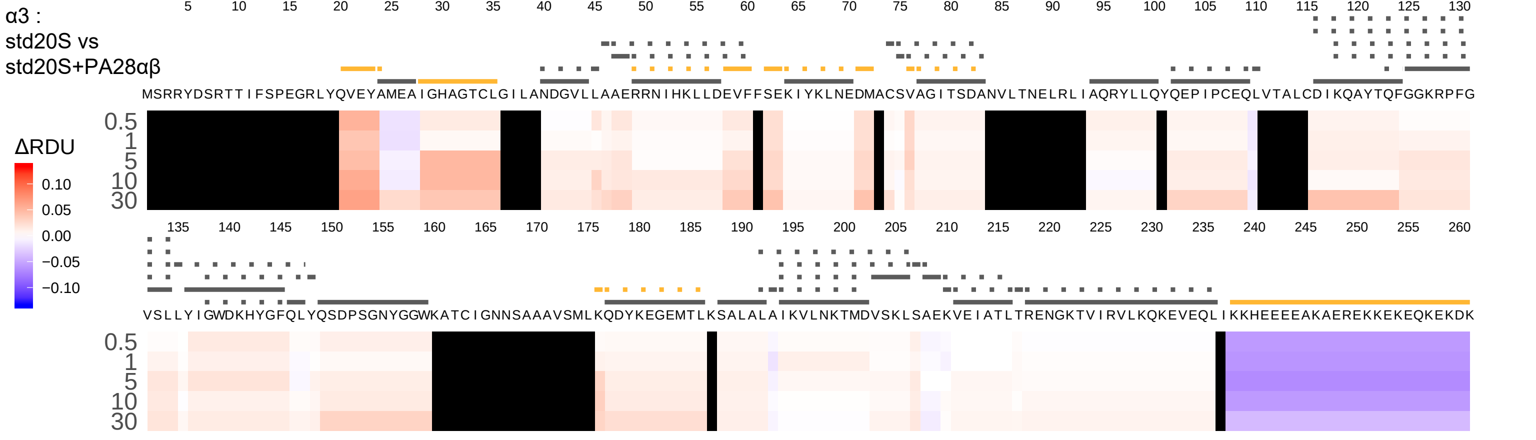
α3 :
std20S vs
std20S+PA28αβ
MSRRYDSRTTIFSPEGRLYQVEYAMEAIGHAGTCLGILANDGVLLAAERRNIHKLLDEVFFSEKIYKLNEDMACSVAGITSDANVLTNELRLIAQRYLLQYQEPIPCEQLVTALCDIKQAYTQFGGKRPFG
VSLLYIGWDKHYGFQLYQSDPSGNYGGWKATCIGNNSAAAVSMLKQDYKEGEMTLKSALALAIKVLNKTMDVSKLSAEKVEIATLTRENGKTVIRVLKQKEVEQLIKKHEEEEAKAEREKKEKEQKEKDK
ΔRDU
0.10
0.05
0.00
−0.05
−0.10
0.5
1
5
10
30

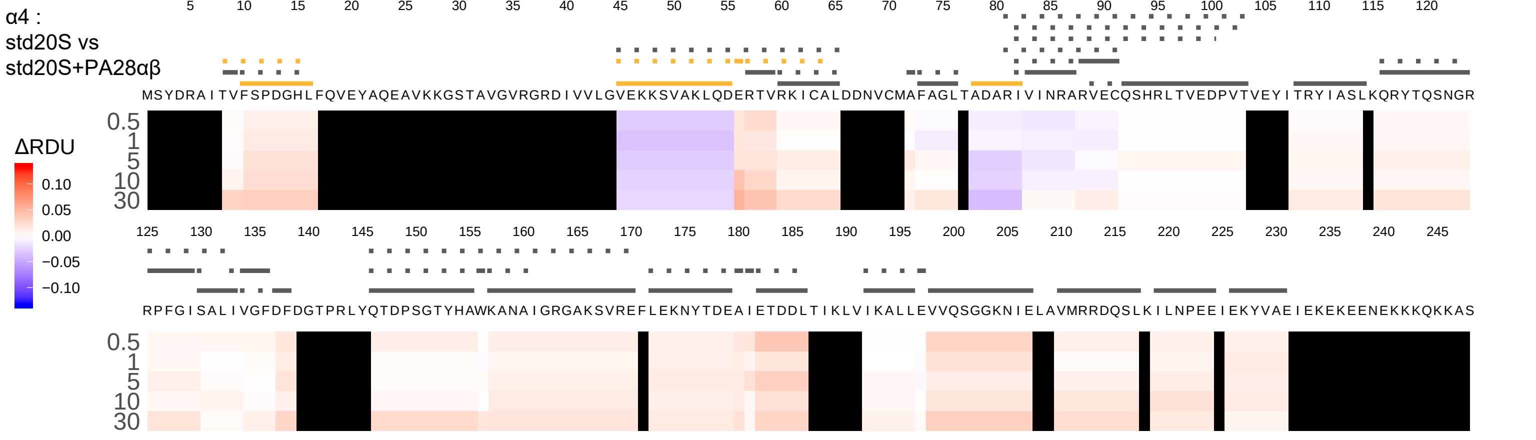

α4 :
std20S vs
std20S+PA28αβ
ΔRDU
0.10
0.05
0.00
−0.05
−0.10
MSYDRAITVFSPDGHLFQVEYAQEAVKKGSTAVGVRGRDIVVLGVEKKSVAKLQDERTVRKICALDDNVCMAFAGLTADARIVINRARVECQSHRLTVEDPVTVEYITRYIASLKQRYTQSNGR
RPFGISALIVGFDFDGTPRLYQTDPSGTYHAWKANAIGRGAKSVREFLEKNYTDEAIETDDLTIKLVIKALLEVVQSGGKNIELAVMRRDQSLKILNPEEIEKYVAEIEKEKEENEKKKQKKAS
0.5
1
5
10
30

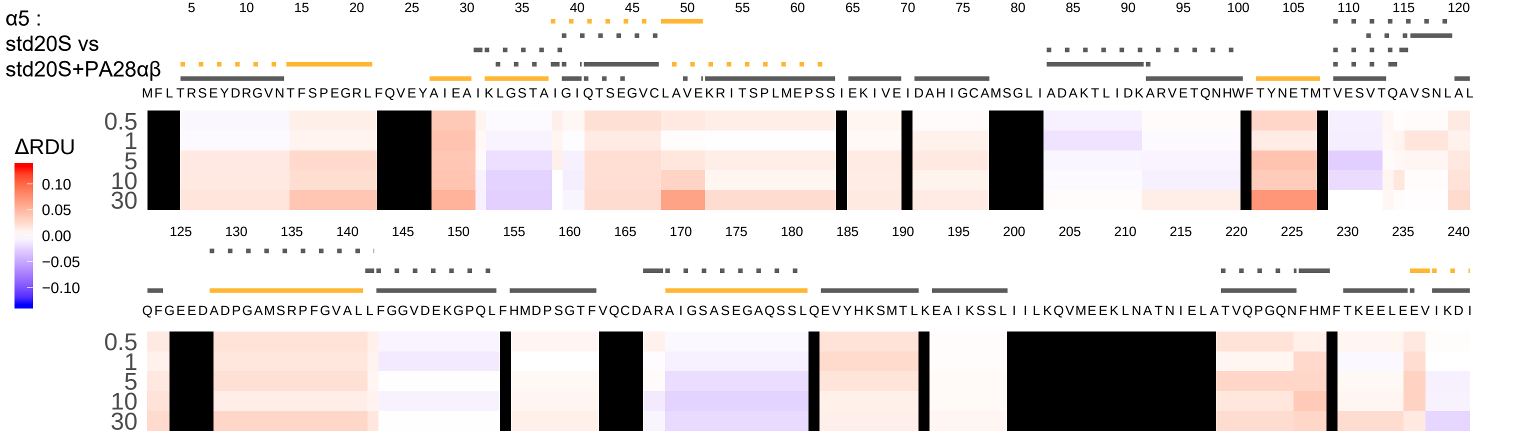

α5 :
std20S vs
std20S+PA28αβ
ΔRDU
0.10
0.05
0.00
−0.05
−0.10
0.5
1
5
10
30
MFLTRSEYDRGVNTFSPEGRLFQVEYAIEAIKLGSTAIGIQTSEGVCLAVEKRITSPLMEPSSIEKIVEIDAHIGCAMSGLIADAKTLIDKARVETQNHWFTYNETMTVESVTQAVSNLAL
QFGEEDADPGAMSRPFGVALLFGGVDEKGPQLFHMDPSGTFVQCDARAIGSASEGAQSSLQEVYHKSMTLKEAIKSSLIIILKQVMEEKLNATNIELATVQPGQNFHMFTKEELEEVIKDI

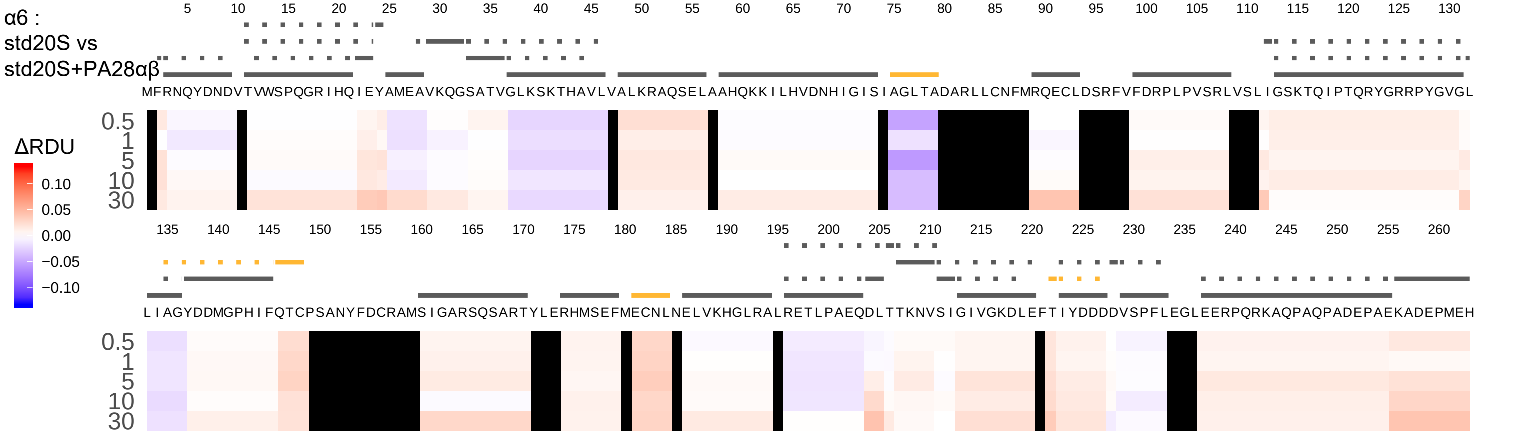
α6 :
std20S vs
std20S+PA28αβ
ΔRDU
0.10
0.05
0.00
−0.05
−0.10
MFRNQYDNDVTVWSPQGRIHQIEYAMEAVKQGSATVGLKSKTHAVLVALKRAQSELAAHQKKILHVDNHIGISIAGLTADARLLCNFMRQECLDSRFVFDRPLPVSRLVSLIGSKTQIPTQRYGRRPYGVGL
LIAGYDDMGPHIFQTCPSANYFDCRAMSIGARSQSARTYLERHMSEFMECNLNELVKHGLRALRETLPAEQDLTTKNVSIGIVGKDLEFTIYDDDDVSPFLEGLEERPQRKAQPAQPADEPAEKADEPMEH
0.5
1
5
10
30

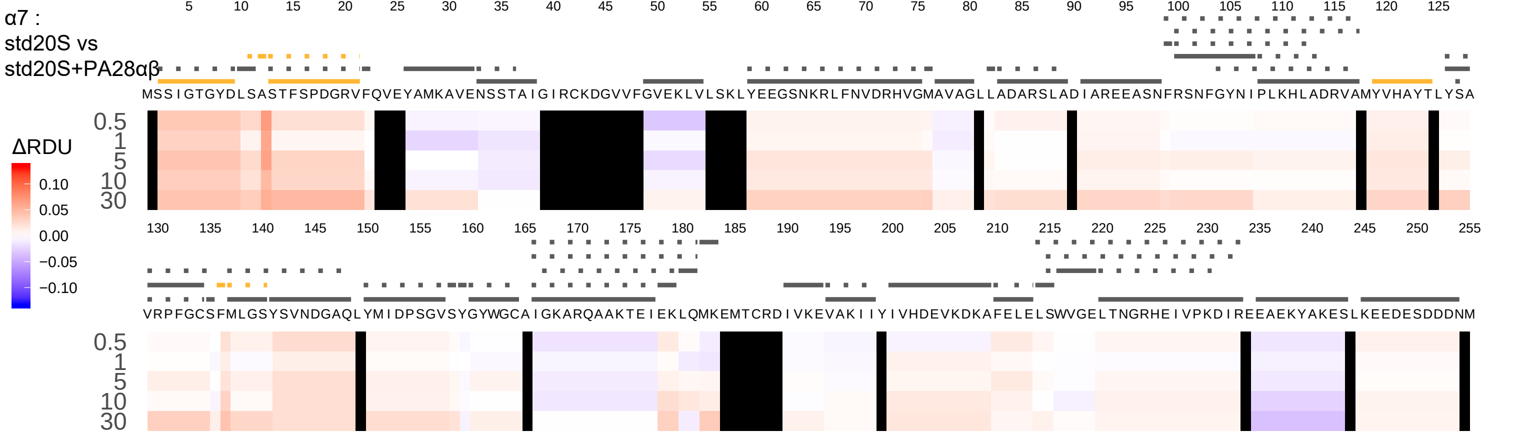

α7 :
std20S vs
std20S+PA28αβ
ΔRDU
0.10
0.05
0.00
−0.05
−0.10
0.5
1
5
10
30
MSSIGTGYDLSASTFSPDGRVFQVEYAMKAVENSSTAIGIRCKDGVVFGVEKLVLSKLYEEGSNKRLFNVDRHVGMAVAGLLADARSLADIAREEASNFRSNFGYNIPLKHLADRVAMYVHAYTLYSA
VRPFGCSFMLGSYSVNDGAQLYMIDPSGVSYGYWGCAIGKARQAAKTEIEKLQMKEMTCRDIVKEVAKIIYIVHDEVKDKAFELELSWVGELTNGRHEIVPKDIREEAEKYAKESLKEEDESDDDNM

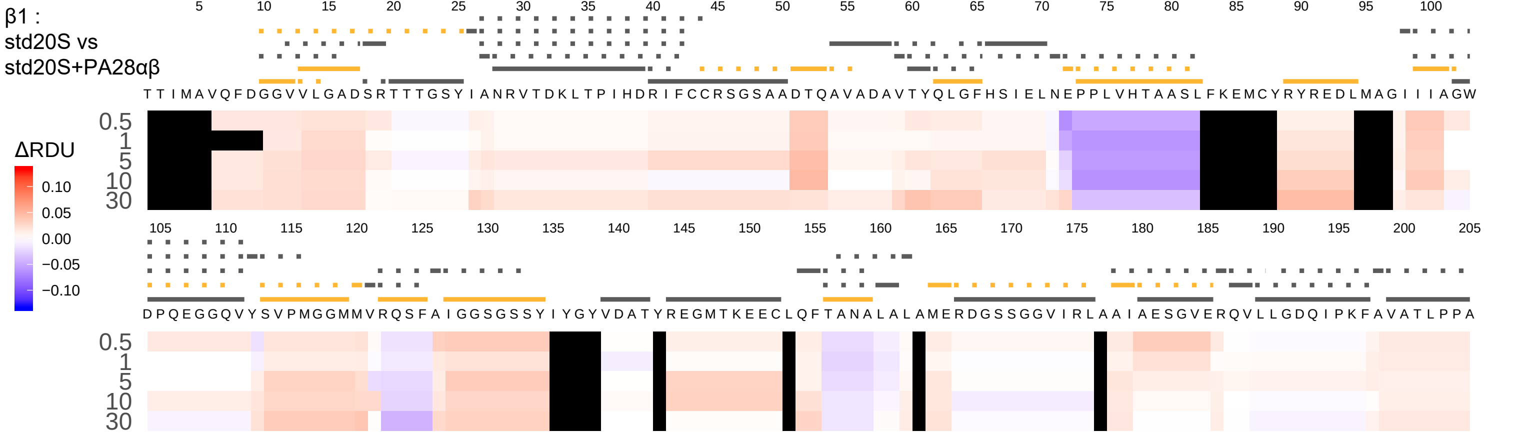

β1 :
std20S vs
std20S+PA28αβ
ΔRDU
0.10
0.05
0.00
−0.05
−0.10
0.5
1
5
10
30
T T I M A V Q F D G G V V L G A D S R T T T G S Y I A N R V T D K L T P I H D R I F C C R S G S A A D T Q A V A D A V T Y Q L G F H S I E L N E P P L V H T A A S L F K E M C Y R Y R E D L M A G I I I A G W
D P Q E G G Q V Y S V P M G G M M V R Q S F A I G G S G S S Y I Y G Y V D A T Y R E G M T K E E C L Q F T A N A L A L A M E R D G S S G G V I R L A A I A E S G V E R Q V L L G D Q I P K F A V A T L P P A

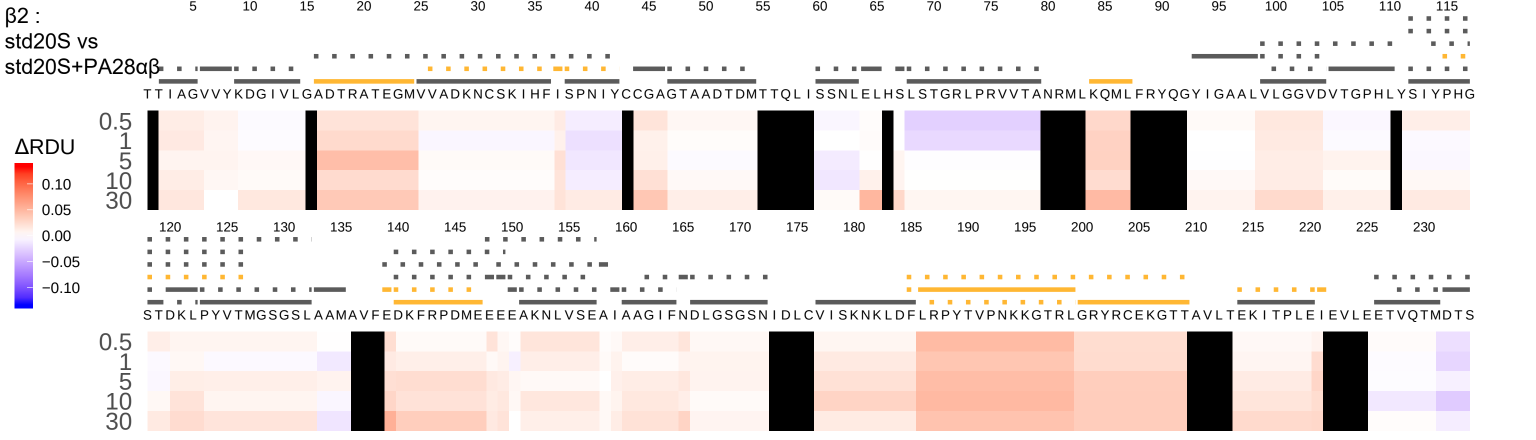
β2 :
std20S vs
std20S+PA28αβ
ΔRDU
0.10
0.05
0.00
−0.05
−0.10
0.5
1
5
10
30
5 10 15 20 25 30 35 40 45 50 55 60 65 70 75 80 85 90 95 100 105 110 115
T T I A G V V Y K D G I V L G A D T R A T E G M V V A D K N C S K I H F I S P N I Y C C G A G T A A D T D M T T Q L I S S N L E L H S L S T G R L P R V V T A N R M L K Q M L F R Y Q G Y I G A A L V L G G V D V T G P H L Y S I Y P H G
120 125 130 135 140 145 150 155 160 165 170 175 180 185 190 195 200 205 210 215 220 225 230
S T D K L P Y V T M G S G S L A A M A V F E D K F R P D M E E E E A K N L V S E A I A A G I F N D L G S G S N I D L C V I S K N K L D F L R P Y T V P N K K G T R L G R Y R C E K G T T A V L T E K I T P L E I E V L E E T V Q T M D T S

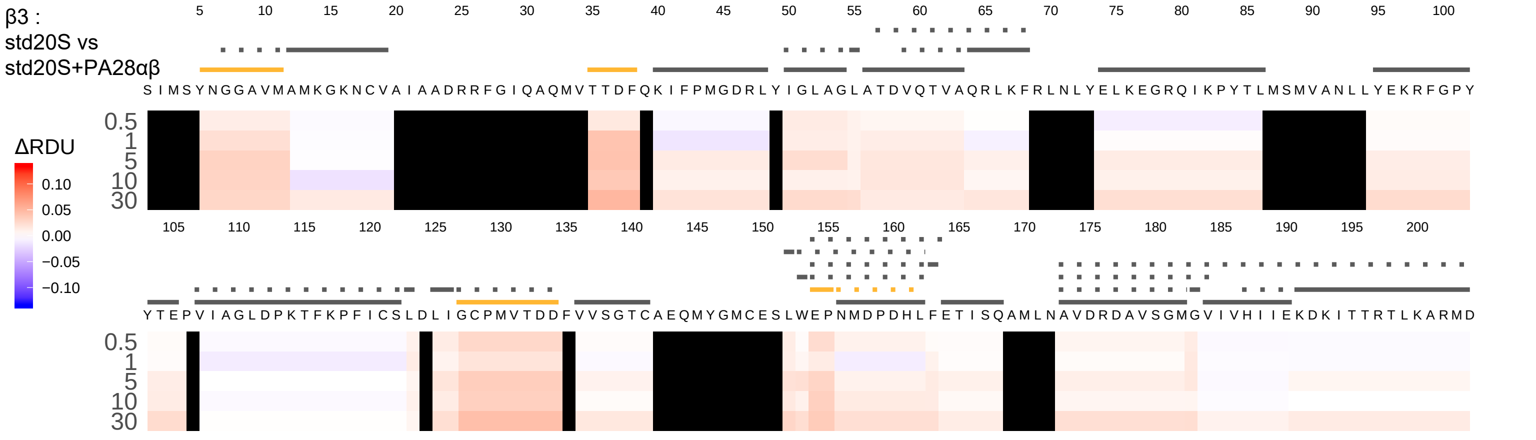
β3 :
std20S vs
std20S+PA28αβ
ΔRDU
0.10
0.05
0.00
−0.05
−0.10
0.5
1
5
10
30
SIMSYNGGAVMAMKGKNCVAIAADRRFGIQAQMVTTDFQKIFPMGDRLYIGLAGLATDVQTVAQRLKFRLNLYELKEGRQIKPYTLMSMVANLLYEKRFGPY
YTEPVIAGLDPKTFKPFICSLDLIGCPMVTDDFVVSGTCAEQMYGMCESLWEPNMDPDHLFETISQAMLNAVDRDAVSGMGVIVHIIEKDKITTRTLKARMD

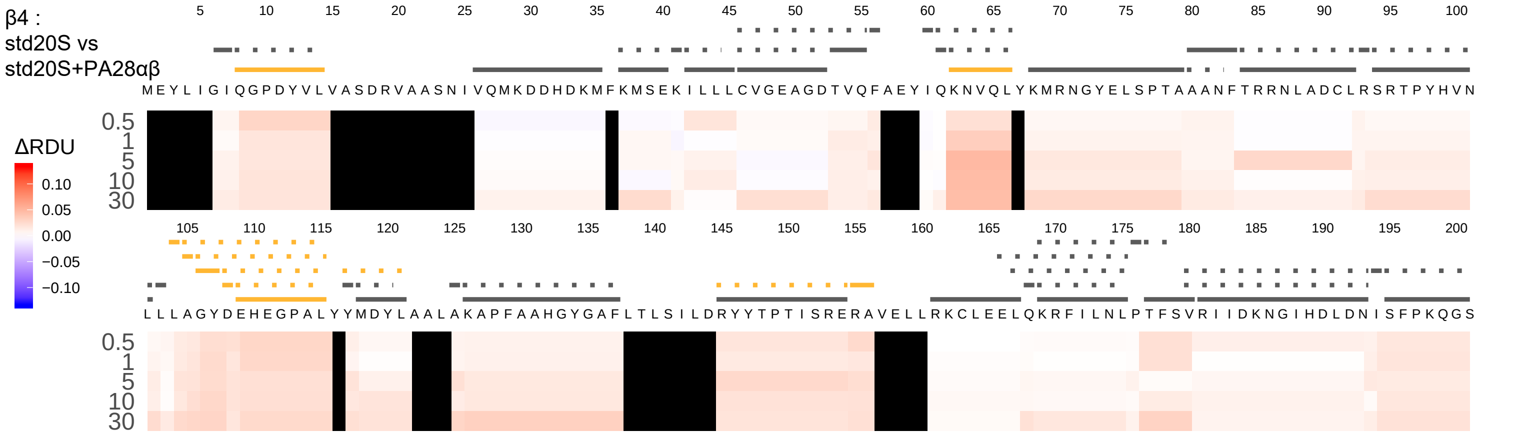

β4 :
std20S vs
std20S+PA28αβ
ΔRDU
0.10
0.05
0.00
−0.05
−0.10
0.5
1
5
10
30
M E Y L I G I Q G P D Y V L V A S D R V A A S N I V Q M K D D H D K M F K M S E K I L L L C V G E A G D T V Q F A E Y I Q K N V Q L Y K M R N G Y E L S P T A A A N F T R R N L A D C L R S R T P Y H V N
L L L A G Y D E H E G P A L Y Y M D Y L A A L A K A P F A A H G Y G A F L T L S I L D R Y Y T P T I S R E R A V E L L R K C L E E L Q K R F I L N L P T F S V R I I D K N G I H D L D N I S F P K Q G S

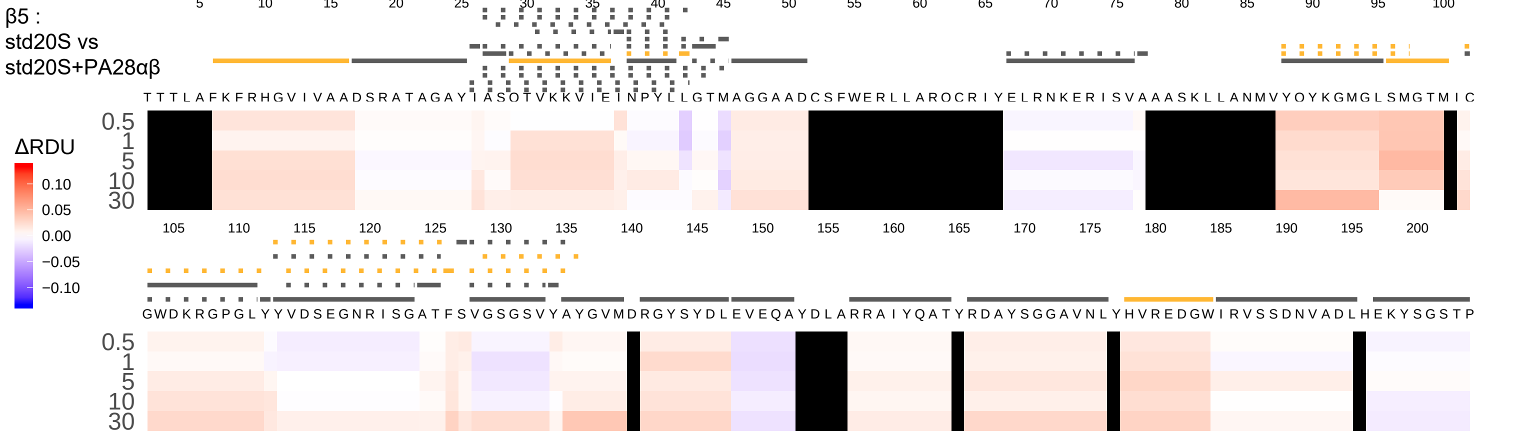
β5 :
std20S vs
std20S+PA28αβ
ΔRDU
0.10
0.05
0.00
−0.05
−0.10
0.5
1
5
10
30
TTTLAFKFRHGVIVAADSRATAGAYIASQTVKKVIEINPYLLGTMAGGAADCSFWERLLARQCRIYELRNKERISVAAASKLLANMVYQYKGMGLSMGTMIC
GWDKRGPGLYYVDSEGNRISGATFSVGSGSVYAYGVMDRGYSYDLEVEQAYDLARRAIYQATYRDAYSGGAVNLYHVREDGWIRVSSDNVADLHEKYSGSTP

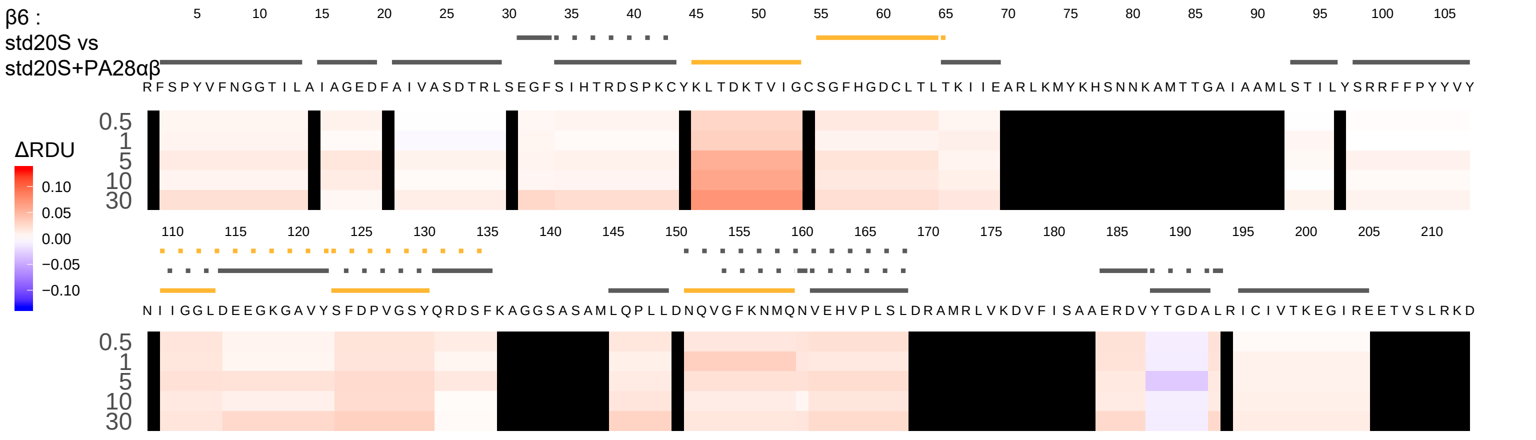

β6 :
std20S vs
std20S+PA28αβ
ΔRDU
0.10
0.05
0.00
−0.05
−0.10
0.5
1
5
10
30
RFSPYVFNGGTILAIAGEDFAIVASDTRLSEGFSIHTRDSPKCYKLTDKTVIGCSGFHGDCLTLTKIIEARLKMYKHSNNKAMTTGAIAAMLSTILYSRRFFPYYVY
NIIGGLDEEGKGAVYSFDPVGSYQRDSFKAGGSASAMLQPLLDNQVGFKNMQNVEHVPLSLDRAMRLVKDVFISAAERDVYTGDALRICIVTKEGIREETVSLRKD

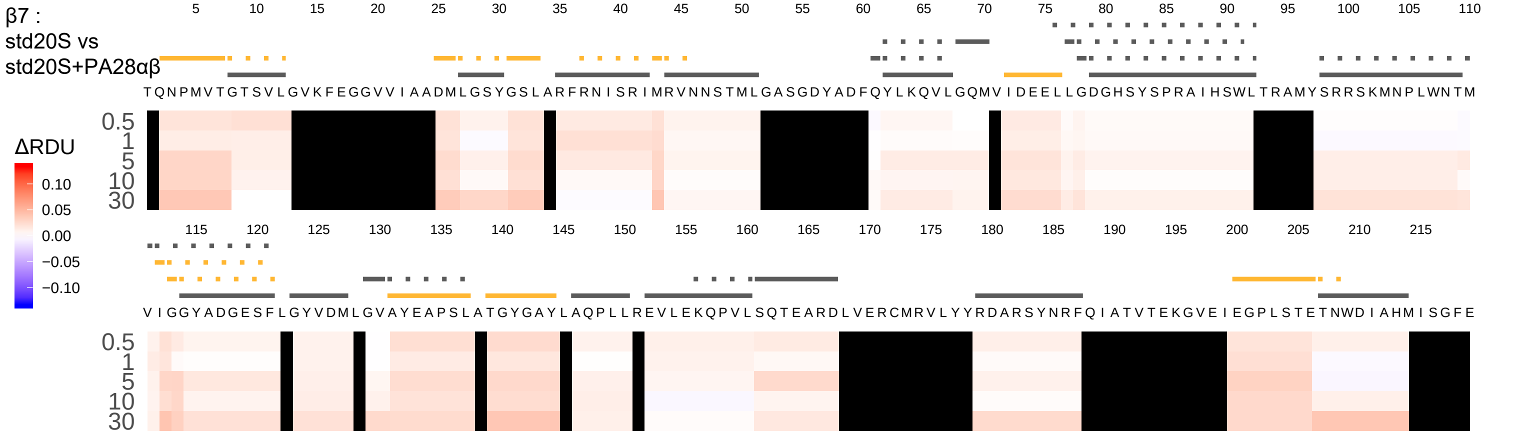
β7 :
std20S vs
std20S+PA28αβ
5 10 15 20 25 30 35 40 45 50 55 60 65 70 75 80 85 90 95 100 105 110
T Q N P M V T G T S V L G V K F E G G V V I A A D M L G S Y G S L A R F R N I S R I M R V N N S T M L G A S G D Y A D F Q Y L K Q V L G Q M V I D E E L L G D G H S Y S P R A I H S W L T R A M Y S R R S K M N P L W N T M
0.5
1
5
10
30
ΔRDU
0.10
0.05
0.00
−0.05
−0.10
115 120 125 130 135 140 145 150 155 160 165 170 175 180 185 190 195 200 205 210 215
V I G G Y A D G E S F L G Y V D M L G V A Y E A P S L A T G Y G A Y L A Q P L L R E V L E K Q P V L S Q T E A R D L V E R C M R V L Y Y R D A R S Y N R F Q I A T V T E K G V E I E G P L S T E T N W D I A H M I S G F E
0.5
1
5
10
30

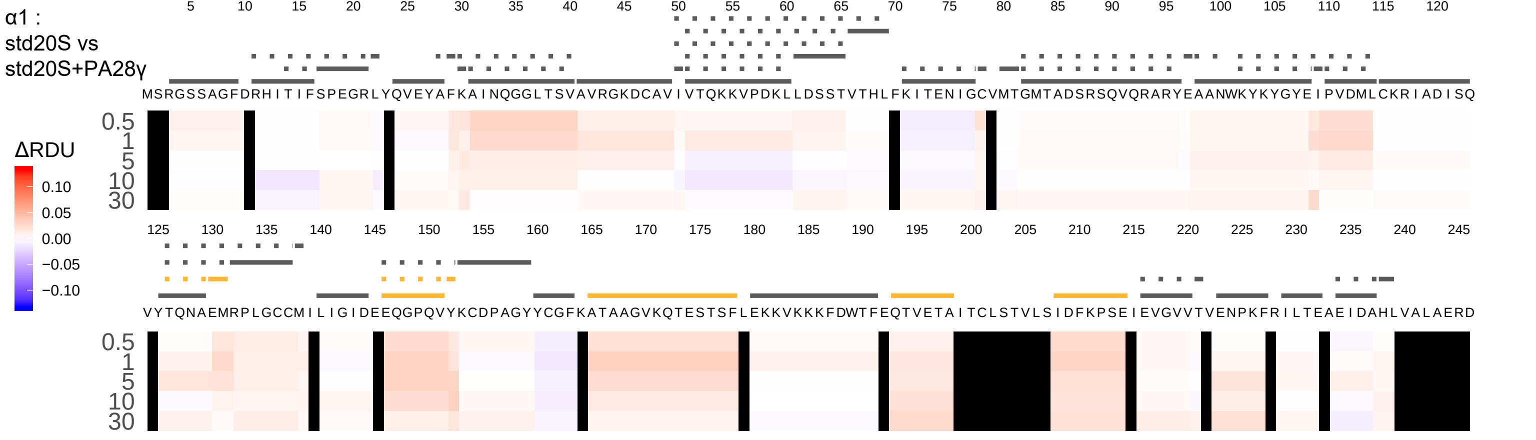

α1 :
std20S vs
std20S+PA28γ
ΔRDU
0.10
0.05
0.00
−0.05
−0.10
0.5
1
5
10
30
MSRGSSAGFDRHITIFSPEGRLYQVEYAFKAINQGGLTSVAVRGKDCAVIVTQKKVPDKLLDSSTVTHLFKITENIGCVMTGMTADSRSQVQRARYEAANWKYKYGYEIPVDMLCKRIADISQ
VYTQNAEMRPLGCCMILIGIDEEQGPQVYKCDPAGYYCGFKATAAGVKQTESTSFLEKKVKKKFDWTFEQTVETAITCLSTVLSIDFKPSEIEVGVVTVENPKFRILTEAEIDAHLVALAERD

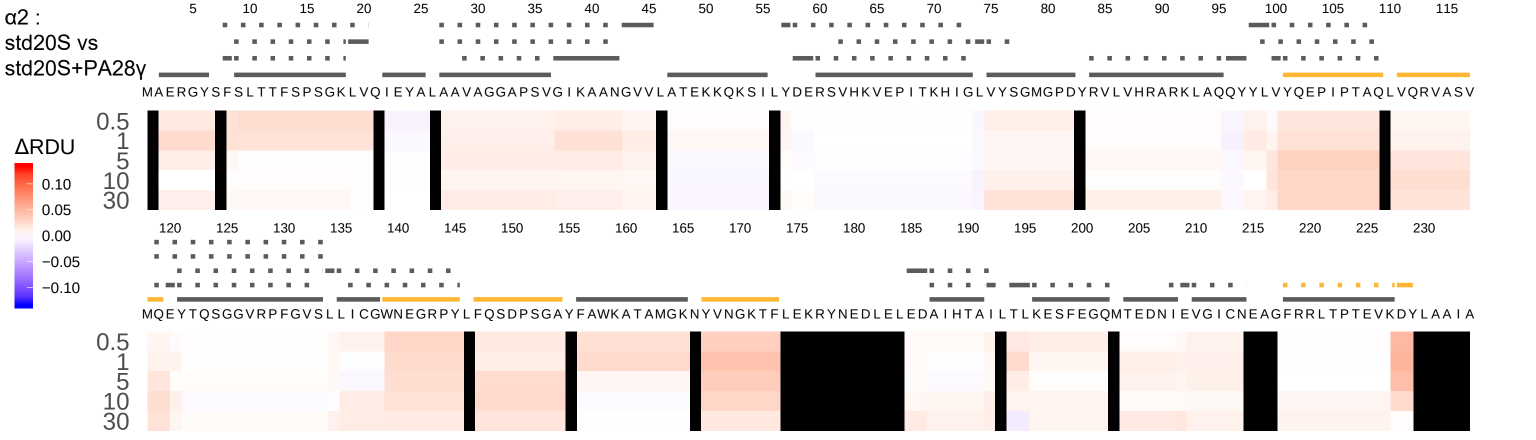
α2 :
std20S vs
std20S+PA28γ
ΔRDU
0.10
0.05
0.00
−0.05
−0.10
0.5
1
5
10
30
MAERGYSFSLTTFSPSGKLVQIEYALAAVAGGAPSVGIKAANGVVLATEKKQKSILYDERSVHKVEPITKHIGLVYSGMGPDYRVLVHRARKLAQQYYLVYQEPIPTAQLVQRVASV
MQEYTQSGGVRPFGVSLLICGWNEGRPYLFQSDPSGAYFAWKATAMGKNYVNGKTFLEKRYNEDLELEDAIHTAILTLKESFEGQMTEDNIEVGICNEAGFRRLTPTEVKDYLAAIA

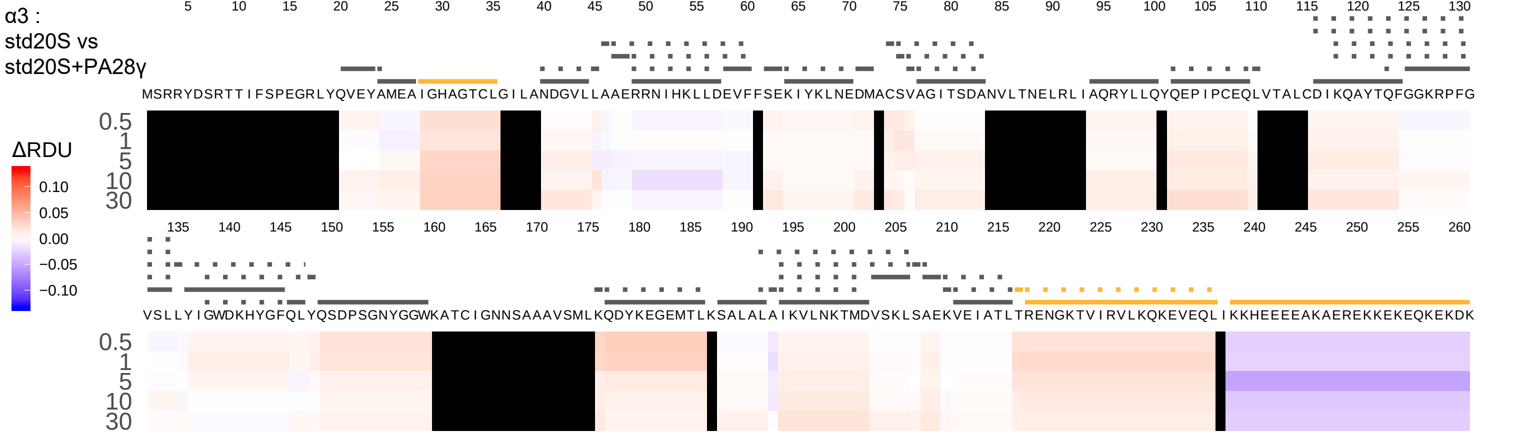

α3 :
std20S vs
std20S+PA28γ
ΔRDU
0.10
0.05
0.00
−0.05
−0.10
0.5
1
5
10
30
MSRRYDSRTTIFSPEGRLYQVEYAMEAIGHAGTCLGILANDGVLLAAERRNIHKLLDEVFFSEKIYKLNEDMACSVAGITSDANVLTNELRLIAQRYLLQYQEPIPCEQLVTALCDIKQAYTQFGGKRPFG
VSLLYIGWDKHYGFQLYQSDPSGNYGGWKATCIGNNSAAAVSMLKQDYKEGEMTLKSALALAIKVLNKTMDVSKLSAEKVEIATLTRENGKTVIRVLKQKEVEQLIKKHEEEEAKAEREKKEKEQKEKDK

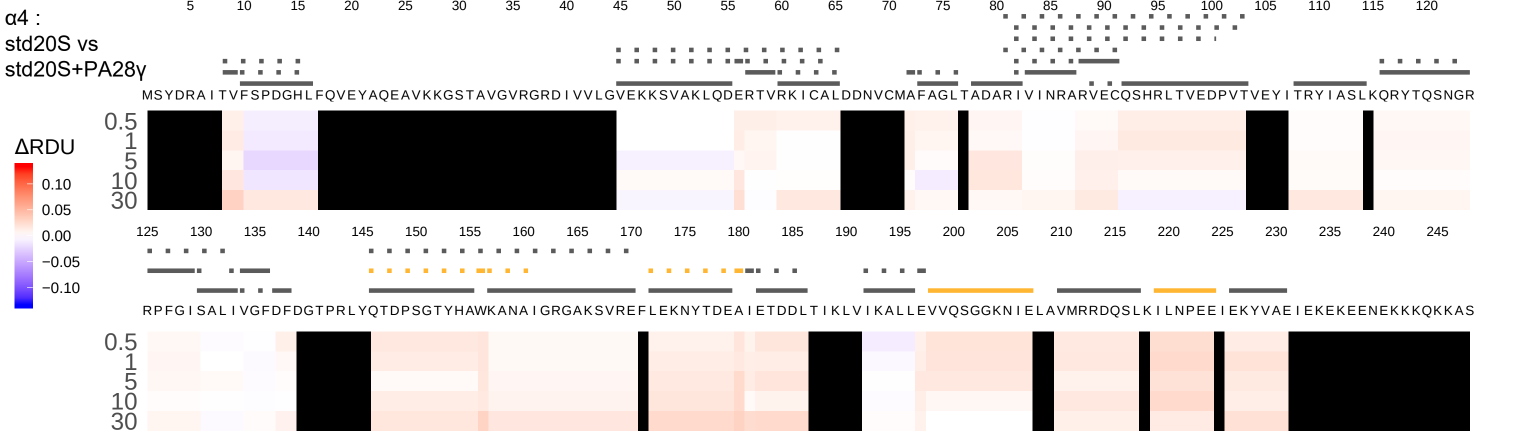
α4 :
std20S vs
std20S+PA28γ
ΔRDU
0.10
0.05
0.00
−0.05
−0.10
0.5
1
5
10
30
MSYDRAITVFSPDGHLFQVEYAQEAVKKGSTAVGVRGRDIVVLGVEKKSVAKLQDERTVRKICALDDNVCMAFAGLTADARIVINRARVECQSHRLTVEDPVTVEYITRYIASLKQRYTQSNGR
RPFGISALIVGFDFDGTPRLYQTDPSGTYHAWKANAIGRGAKSVREFLEKNYTDEAIETDDLTIKLVIKALLEVVQSGGKNIELAVMRRDQSLKILNPEEIEKYVAEIEKEKEENEKKKQKKAS

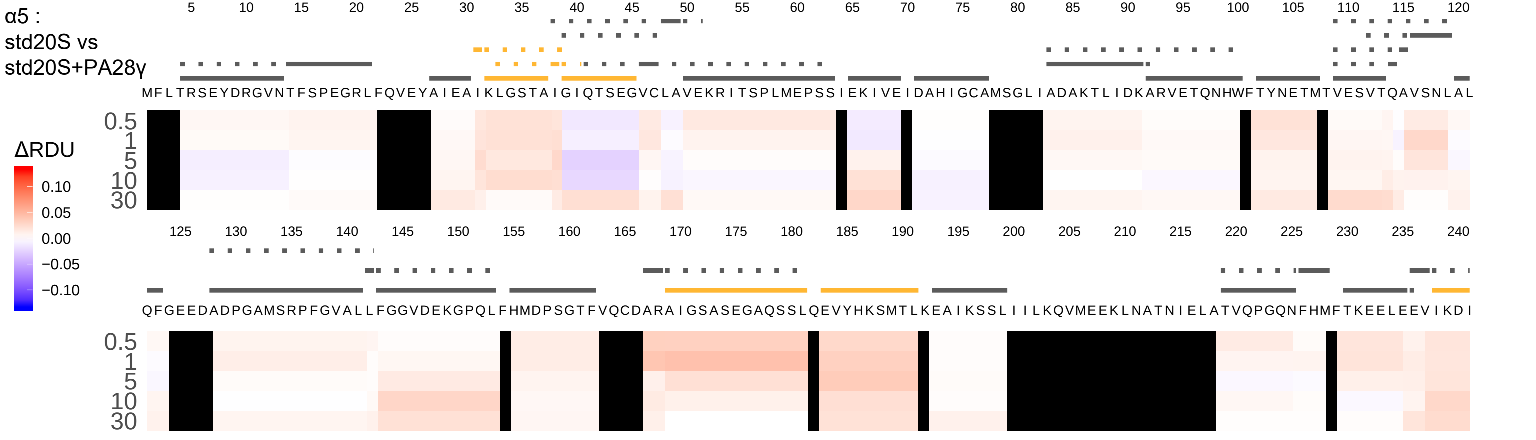
α5 :
std20S vs
std20S+PA28γ
5 10 15 20 25 30 35 40 45 50 55 60 65 70 75 80 85 90 95 100 105 110 115 120
MFLTRSEYDRGVNTFSPEGRLFQVEYAIEAIKLGSTAIGIQTSEGVCLAVEKRITSPLMEPSSIEKIVEIDAHIGCAMSGLIADAKTLIDKARVETQNHWFTYNETMTVESVTQAVSNLAL
0.5
1
5
10
30
ΔRDU
0.10
0.05
0.00
−0.05
−0.10
125 130 135 140 145 150 155 160 165 170 175 180 185 190 195 200 205 210 215 220 225 230 235 240
QFGEEDADPGAMSRPFGVALLFGGVDEKGPQLFHMDPSGTFVQCDARAIGSASEGAQSSLQEVYHKSMTLKEAIKSSLIILKQVMEEKLNATNIELATVQPGQNFHMFTKEELEEVIKDI
0.5
1
5
10
30

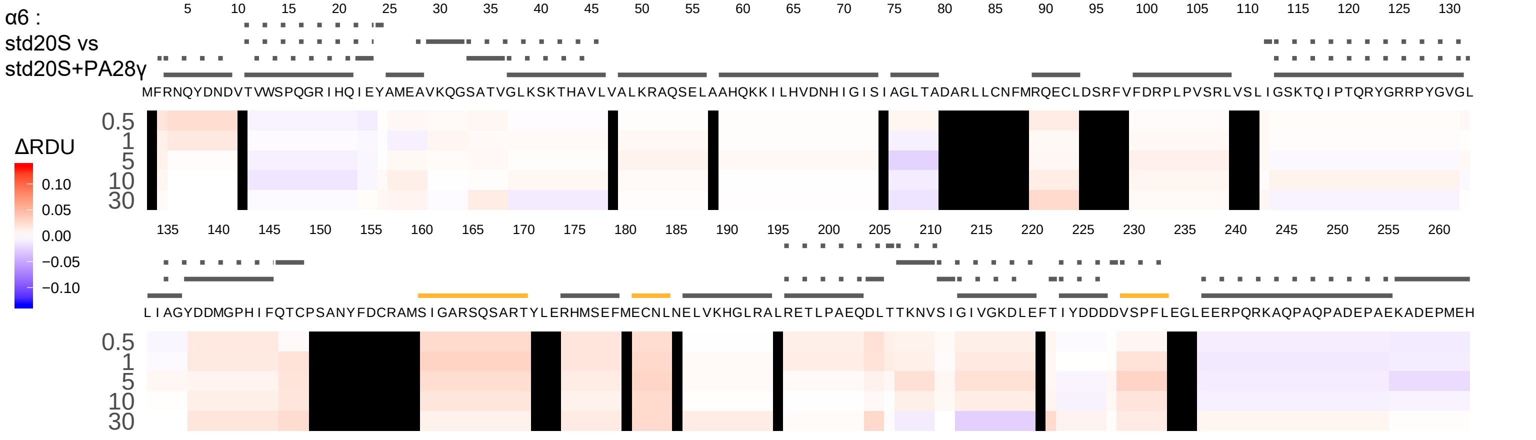

α6 :
std20S vs
std20S+PA28γ
5 10 15 20 25 30 35 40 45 50 55 60 65 70 75 80 85 90 95 100 105 110 115 120 125 130
MFRNQYDNDVTVWSPQGRIHQIEYAMEAVKQGSATVGLKSKTHAVLVALKRAQSELAAHQKKILHVDNHIGISIAGLTADARLLCNFMRQECLDSRFVFDRPLPVSVRLVSLIGSKTQIPTQRYGRRPYGVGL
0.5
1
5
10
30
ΔRDU
0.10
0.05
0.00
−0.05
−0.10
135 140 145 150 155 160 165 170 175 180 185 190 195 200 205 210 215 220 225 230 235 240 245 250 255 260
LIAGYDDMGPHIFQTCPSANYFDCRAMSIGARSQSARTYLERHMSEFMECNLNELVKHGLRALRETLPAEQDLTTKNVSIGIVGKDLEFTIYDDDDVSPFLEGLEERPQRKAQPAQPADEPAEKADEPMEH
0.5
1
5
10
30

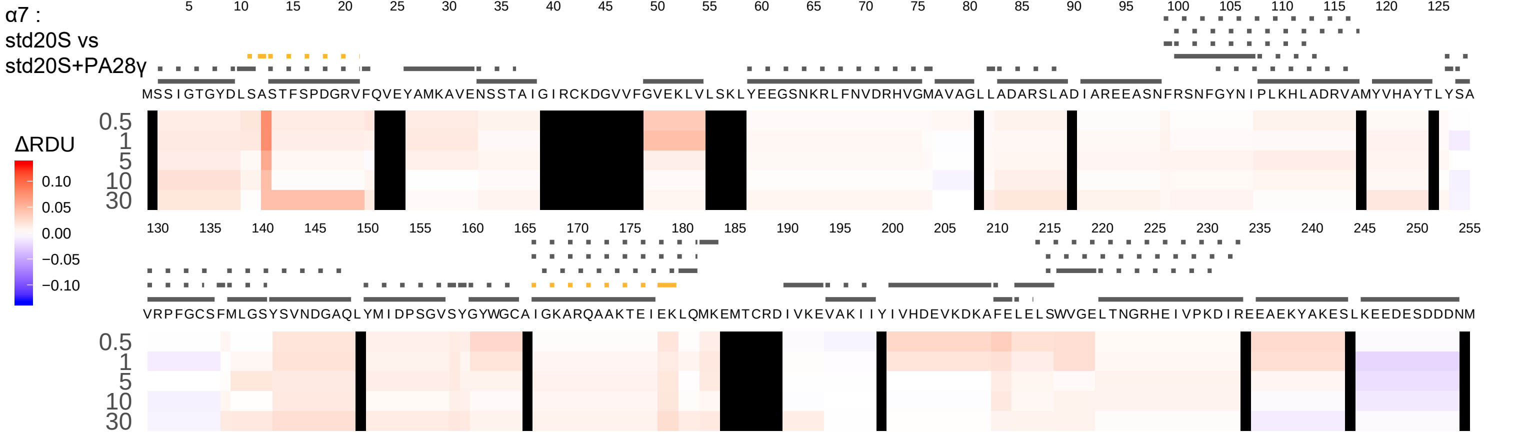

α7 :
std20S vs
std20S+PA28γ
MSSIGTGYDLSASTFSPDGRVFQVEYAMKAVENSSTAIGIRCKDGVVFGVEKLVLSKLYEEGSNKRLFNVDRHVGMAVAGLLADARSLADIAREEASNFRSNFGYNIPLKHLADRVAMYVHAYTLYSA
VRPFGCSFMLGSYSVNDGAQLYMIDPSGVSYGYWGCAIGKARQAAKTEIEKLQMKEMTCRDIVKEVAKIIYIVHDEVKDKAFELELSWVGELTNGRHEIVPKDIREEAEKYAKESLKEEDESDDDNM
ΔRDU
0.10
0.05
0.00
−0.05
−0.10
0.5
1
5
10
30

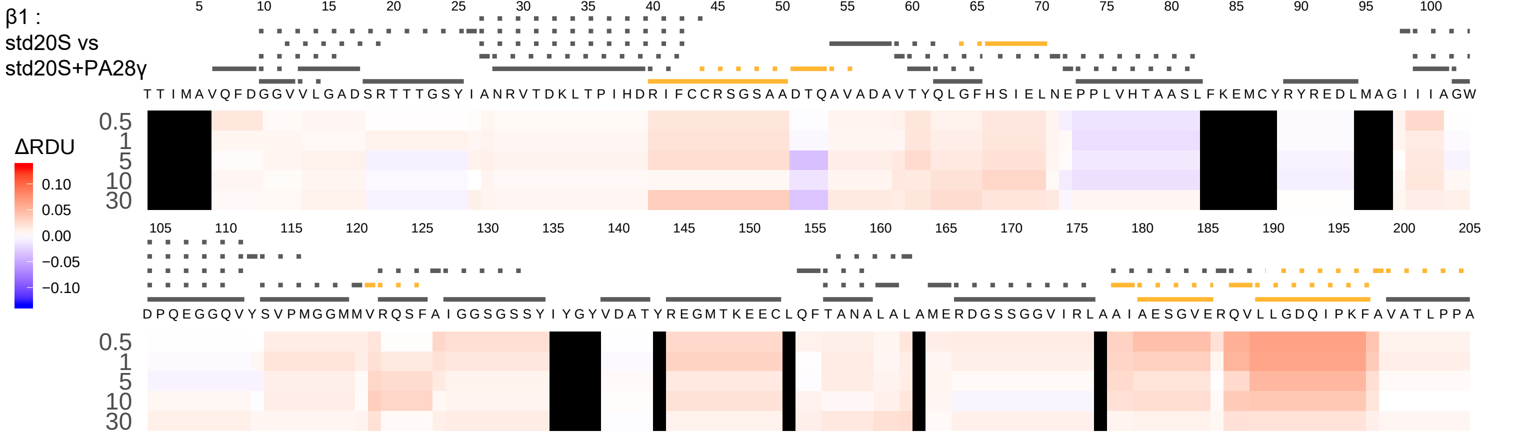

β1 :
std20S vs
std20S+PA28γ
TTIMAVQFDGGVVLGADSRTTTGSYIANRVTDKLTPIHDRIFCCRSGSAADTQAVADAVTYQLGFHSIELNEPPLVHTAASLFKEMCYRYREDLMAGIIIAGW
DPQEGGQVYSVPMGGMMVRQSFAIGGSGSSYIYGYVDATYREGMTKEECLQFTANALALAMERDGSSGGVIRLAAIAESGVERQVLLGDQIPKFAVATLPPA
ΔRDU
0.10
0.05
0.00
−0.05
−0.10
0.5
1
5
10
30

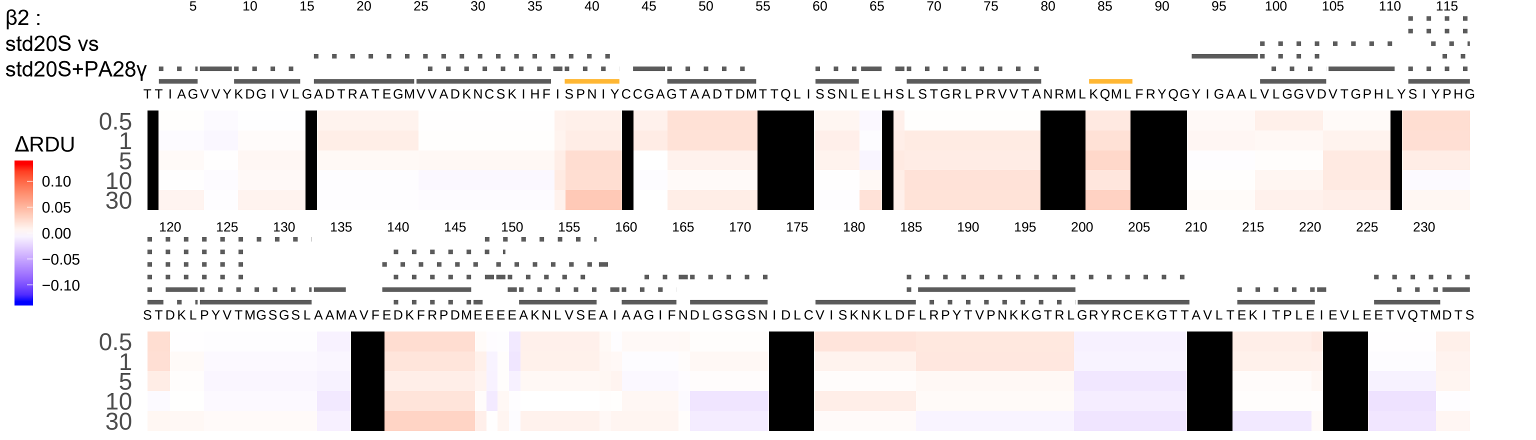
β2 :
std20S vs
std20S+PA28γ
TTIAGVVYKDGIVLGADTRATEGMVVADKNCSKIHFISPNIYCCGAGTAADTDMTTQLISSNLELHSLSTGRLPRVVTANRMLKQMLFRYQGYIGAALVLGGVDVTGPHLYSIYPHG
0.5
1
5
10
30
ΔRDU
0.10
0.05
0.00
−0.05
−0.10
STDKLPYVTMGSGSLAAMAVFEDKFRPDMEEEEAKNLVSEAIAAGIFNDLGSGSNIDLCVISKNKLDFLRPYTVPNKKGTRLGRYRCEKGTTAVLTEKITPLEIEVLEETVQTMDTS

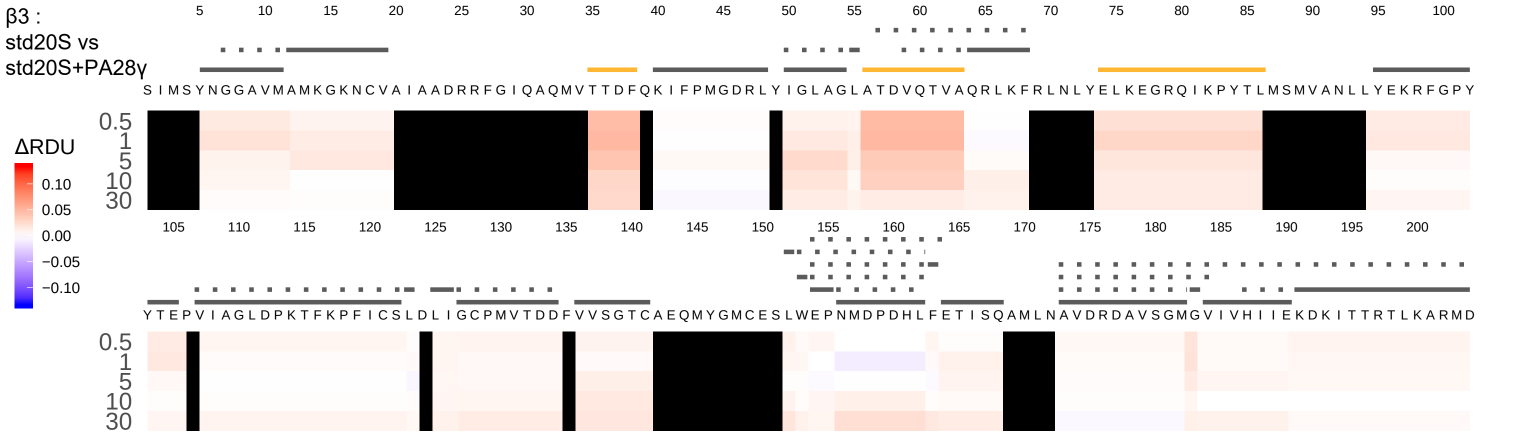
β3 :
std20S vs
std20S+PA28γ
ΔRDU
0.10
0.05
0.00
−0.05
−0.10
0.5
1
5
10
30
S I M S Y N G G A V M A M K G K N C V A I A A D R R F G I Q A Q M V T T D F Q K I F P M G D R L Y I G L A G L A T D V Q T V A Q R L K F R L N L Y E L K E G R Q I K P Y T L M S M V A N L L Y E K R F G P Y
Y T E P V I A G L D P K T F K P F I C S L D L I G C P M V T D D F V V S G T C A E Q M Y G M C E S L W E P N M D P D H L F E T I S Q A M L N A V D R D A V S G M G V I V H I I E K D K I T T R T L K A R M D

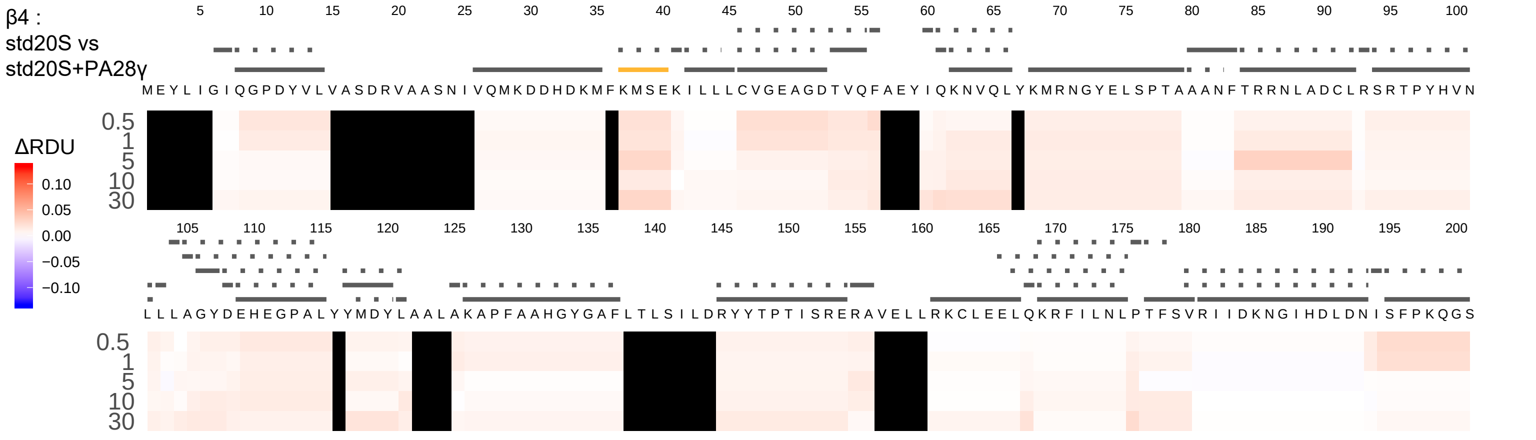

β4 :
std20S vs
std20S+PA28γ
ΔRDU
0.10
0.05
0.00
−0.05
−0.10
0.5
1
5
10
30
MEYLIGIQGPDYVLVASDRVAASNIVQMKDDHDKMFKMSEKILLLCVGEAGDTVQFAEYIQKNVQLYKMRNGYELSPTAAANFTRRNLADCLRSRTPYHVN
LLLAGYDEHEGPALYYMDYLAALAKAPFAAHGYGAFLTLSILDRYYTPTISRERAVELLRKCLEELQKRFILNLPTFSVRIIDKNGIHDLDNISFPKQGS

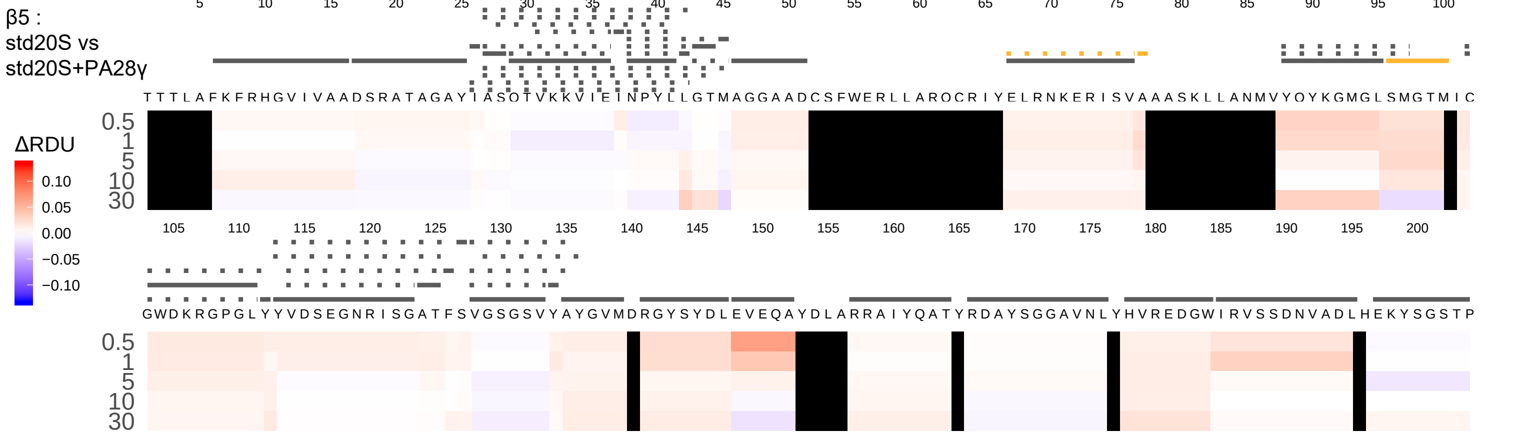
β5 :
std20S vs
std20S+PA28γ
ΔRDU
0.10
0.05
0.00
−0.05
−0.10
0.5
1
5
10
30
TTTLAFKFRHGVIVAADSRATAGAYIASQTVKKVIEINPYLLGTMAGGAADCSFWERLLARQCRIYELRNKERISVAAASKLLANMVYQYKGMGLSMGTMIC
GWDKRGPGLYYVDSEGNRISGATFSVGSGSVYAYGVMDRGYSYDLEVEQAYDLARRAIYQATYRDAYSGGAVNLYHVREDGWIRVSSDNVADLHEKYSGSTP

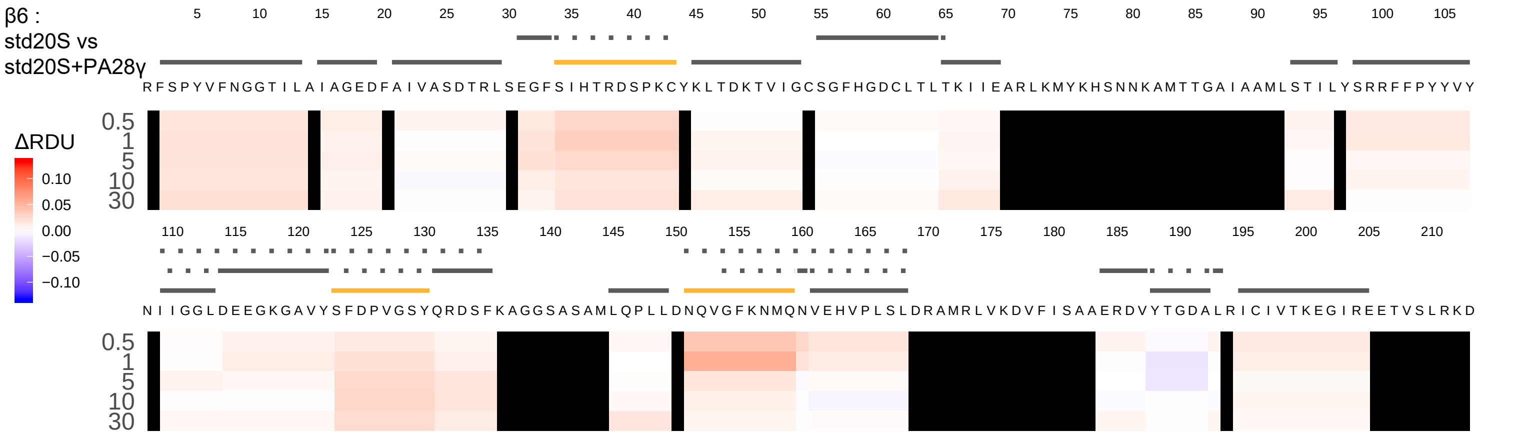
β6 :
std20S vs
std20S+PA28γ
ΔRDU
0.10
0.05
0.00
−0.05
−0.10
0.5
1
5
10
30
RFSPYVFNGGTILAIAGEDFAIVASDTRLSEGFSIHTRDSPKCYKLTDKTVIGCSGFHGDCLTLTKIIEARLKMYKHSNNKAMTTGAIAAMLSTILYSRRFFPYYVY
NIIGGLDEEGKGAVYSFDPVGSYQRDSFKAGGSASAMLQPLLDNQVGFKNMQNVEHVPLSLDRAMRLVKDVFISAAERDVYTGDALRICIVTKEGIREETVSLRKD

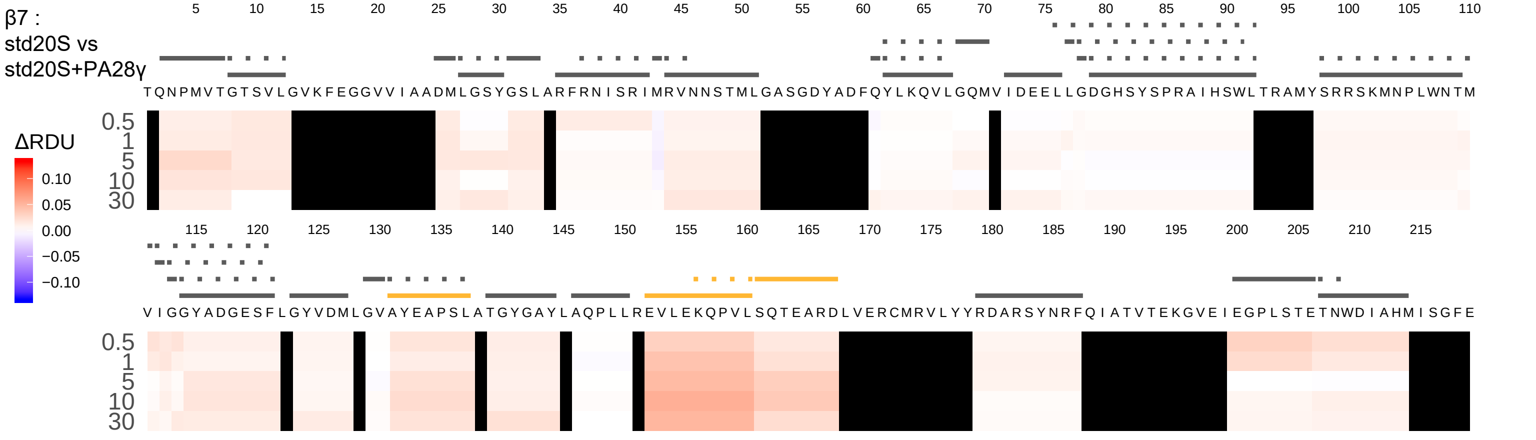
β7 :
std20S vs
std20S+PA28γ
ΔRDU
0.10
0.05
0.00
−0.05
−0.10
0.5
1
5
10
30
TQNPMVTGTSVLGVKFEGGVVIAADMLGSYGSLARFRNISRIMRVNNSTMLGASGDYADFQYLKQVLGQMVIDEELLGDGHSYSPRAIHSWLTRAMYSRRSKMNPLWNTM
VIGGYADGESFLGYVDMLGVAYEAPSLATGYGAYLAQPLLREVLEKQPVLSQTEARDLVERCMRVLYYRDARSYNRFQIATVTEKGVEIEGPLSTETNWDIAHMISGFE

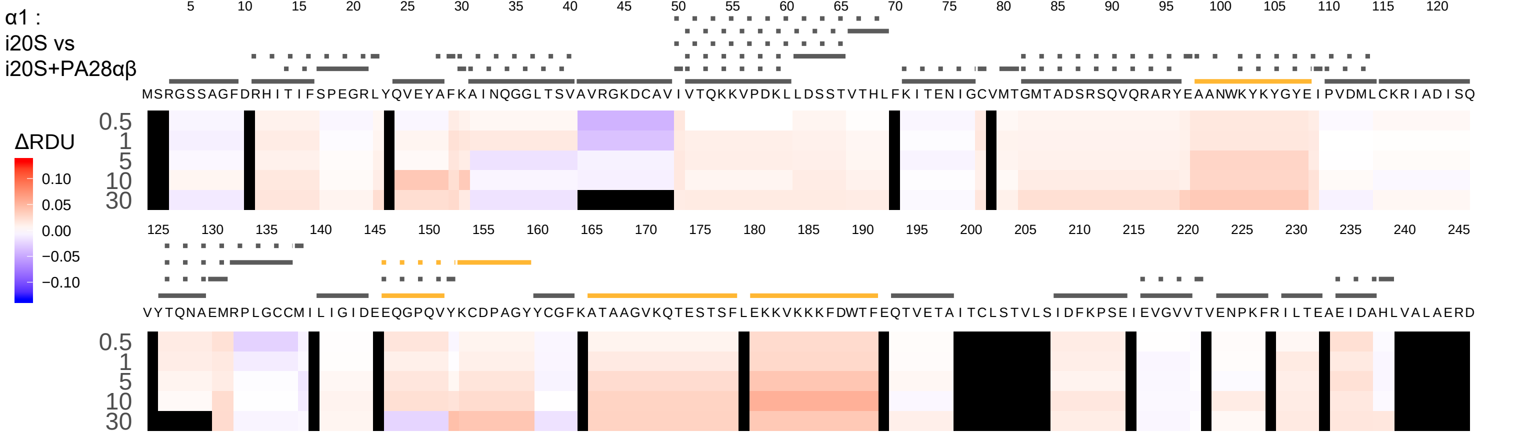
α1 :
i20S vs
i20S+PA28αβ
ΔRDU
0.10
0.05
0.00
−0.05
−0.10
0.5
1
5
10
30
MSRGSSAGFDRHITIFSPEGRLYQVEYAFKAINQGGLTSVAVRGKDCAVIVTQKKVPDKLLDSSTVTHLFKITENIGCVMTGMTADSRSQVQRARYEAANWKYKYGYEIPVDMLCKRIADISQ
VYTQNAEMRPLGCCMILIGIDEEQGPQVYKCDPAGYYCGFKATAAGVKQTESTSFLEKKVKKKFDWTFEQTVETAITCLSTVLSIDFKPSEIEVGVVTVENPKFRILTEAEIDAHLVALAERD

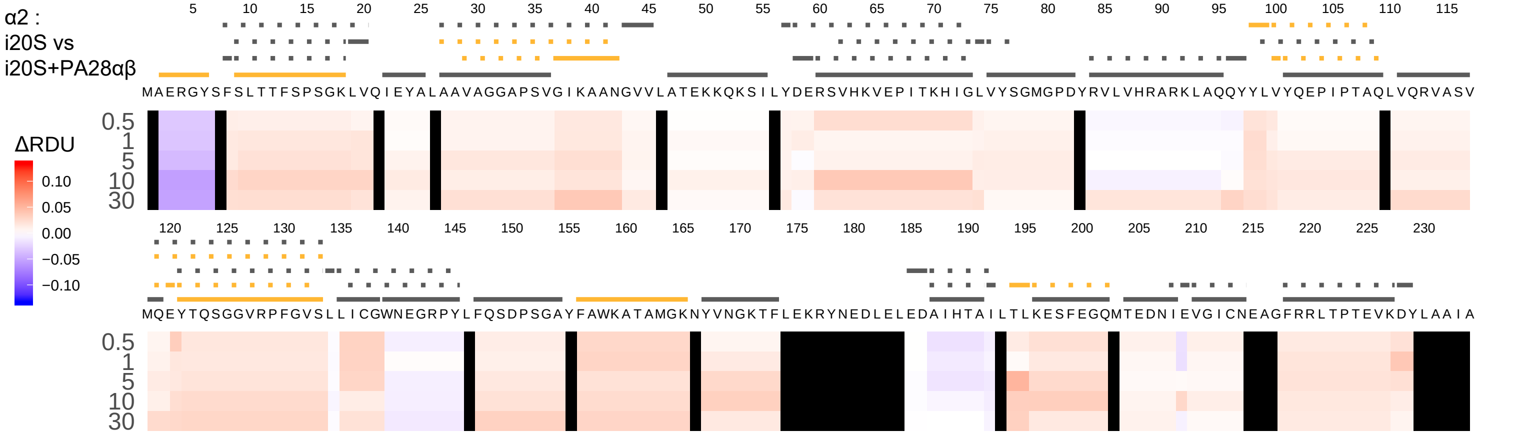
α2 :
i20S vs
i20S+PA28αβ
ΔRDU
0.10
0.05
0.00
−0.05
−0.10
0.5
1
5
10
30
MAERGYSFSLTTFSPSGKLVQIEYALAAVAGGAPSVGIKAANGVVLATEKKQKSILYDERSVHKVEPITKHIGLVYSGMGPDYRVLVHRARKLAQQYYLVYQEPIPTAQLVQRVASV
MQEYTQSGGVRPFGVSLLICGWNEGRPYLFQSDPSGAYFAWKATAMGKNYVNGKTFLEKRYNEDLELEDAIHTAILTLKESFEGQMTEDNIEVGICNEAGFRRLTPTEVKDYLAAIA

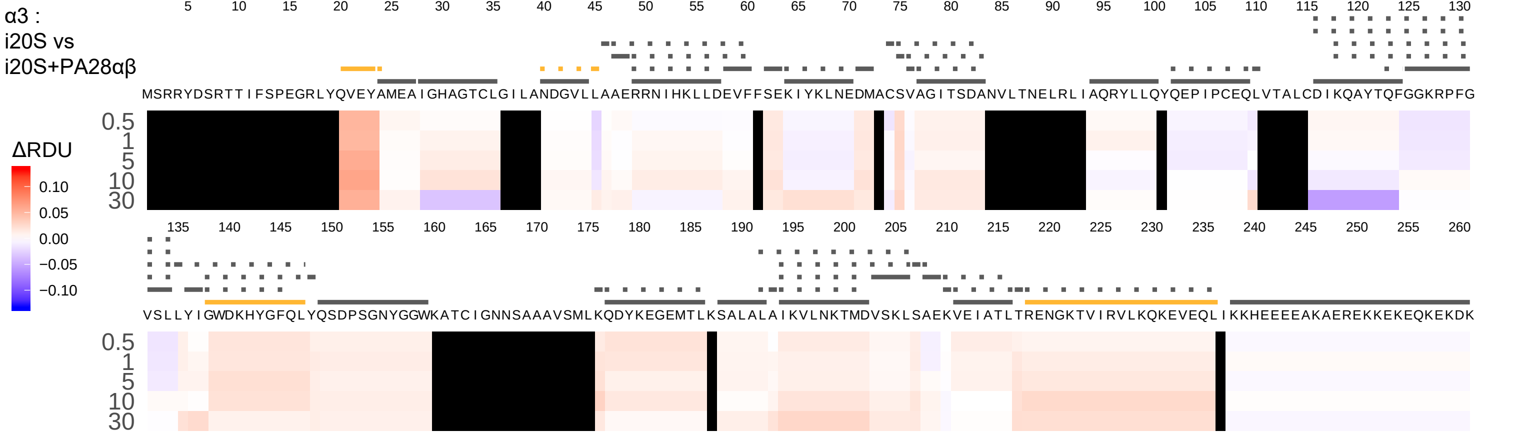
α3 :
i20S vs
i20S+PA28αβ
5 10 15 20 25 30 35 40 45 50 55 60 65 70 75 80 85 90 95 100 105 110 115 120 125 130
MSRRYDSRTTIFSPEGRLYQVEYAMEAIGHAGTCLGILANDGVLLAAERRNIHKLLDEVFFSEKIYKLNEDMACSVAGITSDANVLTNELRLIAQRYLLQYQEPIPCEQLVTALCDIKQAYTQFGGKRPFG
0.5
1
5
10
30
ΔRDU
0.10
0.05
0.00
−0.05
−0.10
135 140 145 150 155 160 165 170 175 180 185 190 195 200 205 210 215 220 225 230 235 240 245 250 255 260
VSLLYIGWDKHYGFQLYQSDPSGNYGGWKATCIGNNSAAAVSMLKQDYKEGEMTLKSALALAIKVLNKTMDVSKLSAEKVEIATLTRENGKTVIRVLKQKEVEQLIKKHEEEEAKAEREKKEKEQKEKDK
0.5
1
5
10
30

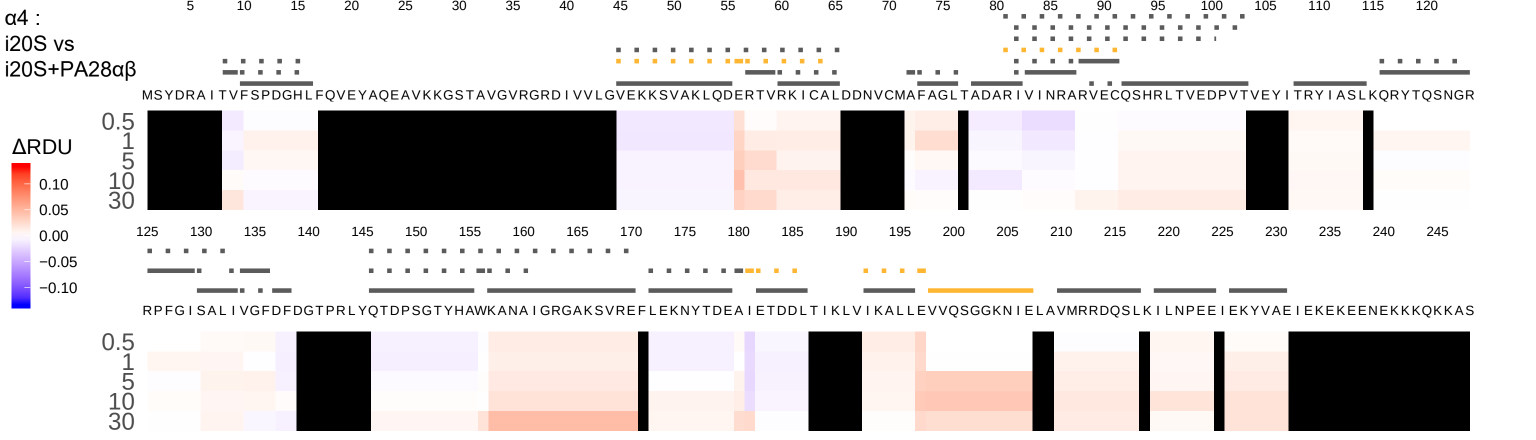
α4 :
i20S vs
i20S+PA28αβ
ΔRDU
0.10
0.05
0.00
−0.05
−0.10
0.5
1
5
10
30
MSYDRAITVFSPDGHLFQVEYAQEAVKKGSTAVGVRGRDIVVLGVEKKSVAKLQDERTVRKICALDDNVCMAFAGLTADARIVINRARVECQSHRLTVEDPVTVEYITRYIASLKQRYTQSNGR
RPFGISALIVGFDFDGTPRLYQTDPSGTYHAWKANAIGRGAKSVREFLEKNYTDEAIETDDLTIKLVIKALLEVVQSGGKNIELAVMRRDQSLKILNPEEIEKYVAEIEKEKEENEKKKQKKAS

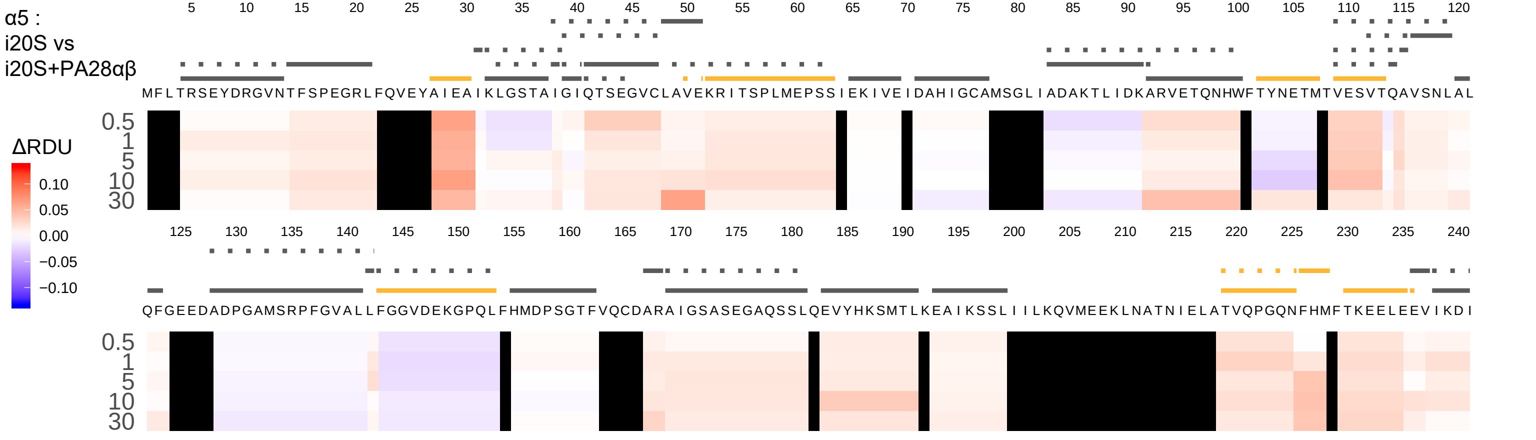
α5 :
i20S vs
i20S+PA28αβ
5 10 15 20 25 30 35 40 45 50 55 60 65 70 75 80 85 90 95 100 105 110 115 120
MFLTRSEYDRGVNTFSPEGRLFQVEYAIEAIKLGSTAIGIQTSEGVCLAVEKRITSPLMEPSSIEKIVEIDAHIGCAMSGLIADAKTLIDKARVETQNHWFTYNETMTVESVTQAVSNLAL
0.5
1
5
10
30
ΔRDU
0.10
0.05
0.00
−0.05
−0.10
125 130 135 140 145 150 155 160 165 170 175 180 185 190 195 200 205 210 215 220 225 230 235 240
QFGEEDADPGAMSRPFGVALLFGGVDEKGPQLFHMDPSGTFVQCDARAIGSASEGAQSSLQEVYHKSMTLKEAIKSSLIIILKQVMEEKLNATNIELATVQPGQNFHMFTKEELEEVIKDI
0.5
1
5
10
30

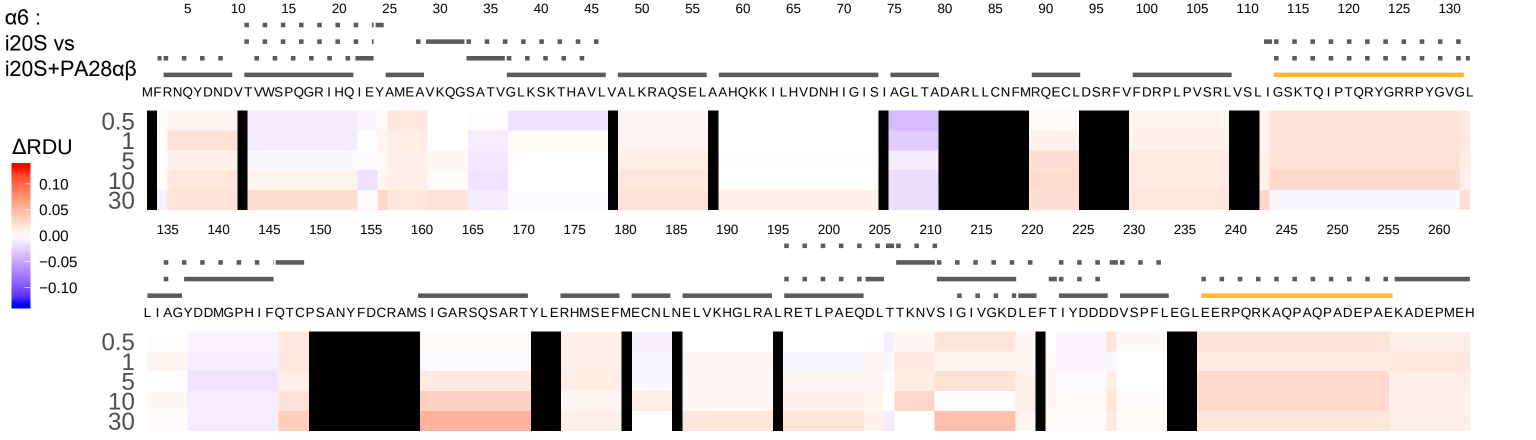

α6 :
i20S vs
i20S+PA28αβ
ΔRDU
0.10
0.05
0.00
−0.05
−0.10
0.5
1
5
10
30
MFRNQYDNDVTVWSPQGRIHQIEYAMEAVKQGSATVGLKSKTHAVLVALKRAQSELAAHQKKILHVDNHIGISIAGLTADARLLCNFMRQECLDSRFVFDRPLPVSRLVSLIGSKTQIPTQRYGRRPYGVGL
LIAGYDDMGPHIFQTCPSANYFDCRAMSIGARSQSARTYLERHMSEFMECNLNELVKHGLRALRETLPAEQDLTTKNVSIGIVGKDLEFTIYDDDDVSPFLEGLEERPQRKAQPAQPADEPAEKADEPMEH

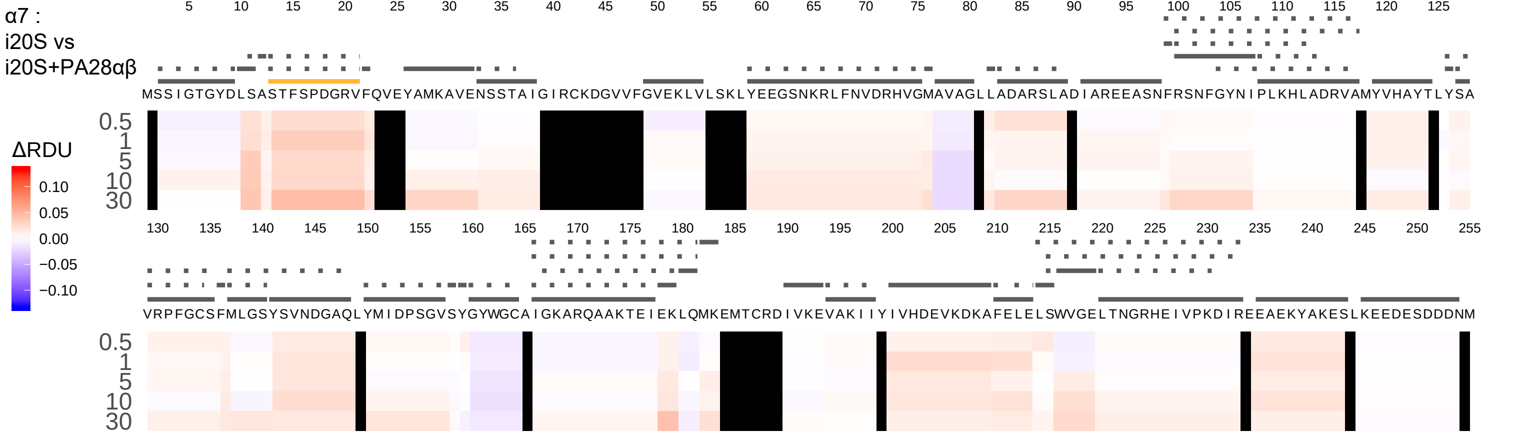
α7 :
i20S vs
i20S+PA28αβ
ΔRDU
0.10
0.05
0.00
−0.05
−0.10
0.5
1
5
10
30
5 10 15 20 25 30 35 40 45 50 55 60 65 70 75 80 85 90 95 100 105 110 115 120 125
MSSIGTGYDLSASTFSPDGRVFQVEYAMKAVENSSTAIGIRCKDGVVFGVEKLVLSKLYEEGSNKRLFNVDRHVGMAVAGLLADARSLADIAREEASNFRSNFGYNIPLKHLADRVAMYVHAYTLYSA
130 135 140 145 150 155 160 165 170 175 180 185 190 195 200 205 210 215 220 225 230 235 240 245 250 255
VRPFGCSFMLGSYSVNDGAQLYMIDPSGVSYGYWGCAIGKARQAAKTEIEKLQMKEMTCRDIVKEVAKIIYIVHDEVKDKAFELELSWVGELTNGRHEIVPKDIREEAEKYAKESLKEEDESDDDNM

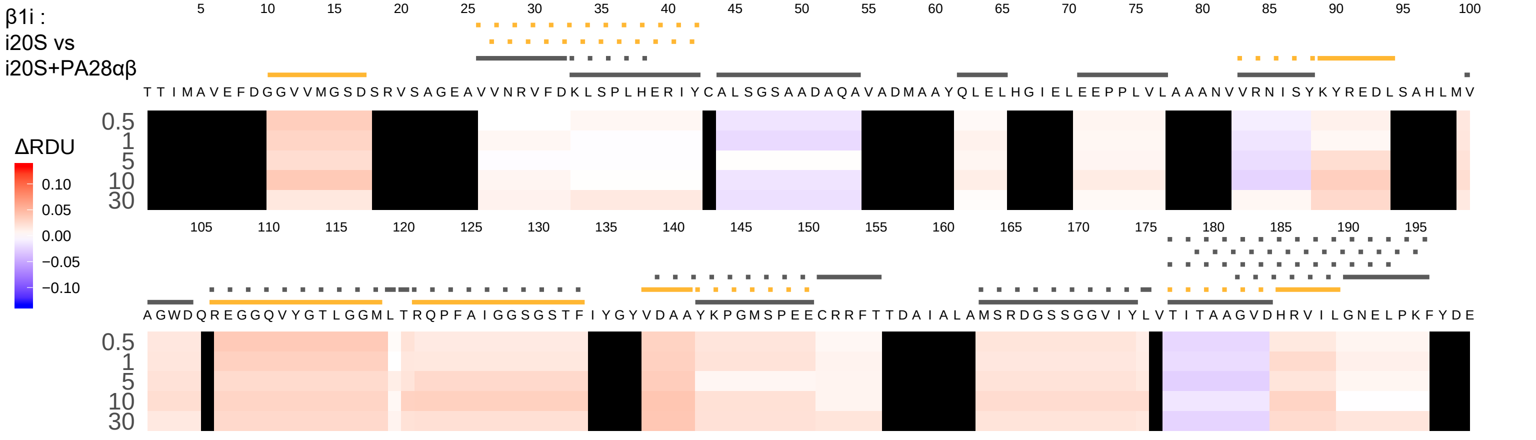
β1i :
i20S vs
i20S+PA28αβ
ΔRDU
0.10
0.05
0.00
−0.05
−0.10
0.5
1
5
10
30
5 10 15 20 25 30 35 40 45 50 55 60 65 70 75 80 85 90 95 100
T T I M A V E F D G G V V M G S D S R V S A G E A V V N R V F D K L S P L H E R I Y C A L S G S A A D A Q A V A D M A A Y Q L E L H G I E L E E P P L V L A A A N V V R N I S Y K Y R E D L S A H L M V
105 110 115 120 125 130 135 140 145 150 155 160 165 170 175 180 185 190 195
A G W D Q R E G G Q V Y G T L G G M L T R Q P F A I G G S G S T F I Y G Y V D A A Y K P G M S P E E C R R F T T D A I A L A M S R D G S S G G V I Y L V T I T A A G V D H R V I L G N E L P K F Y D E

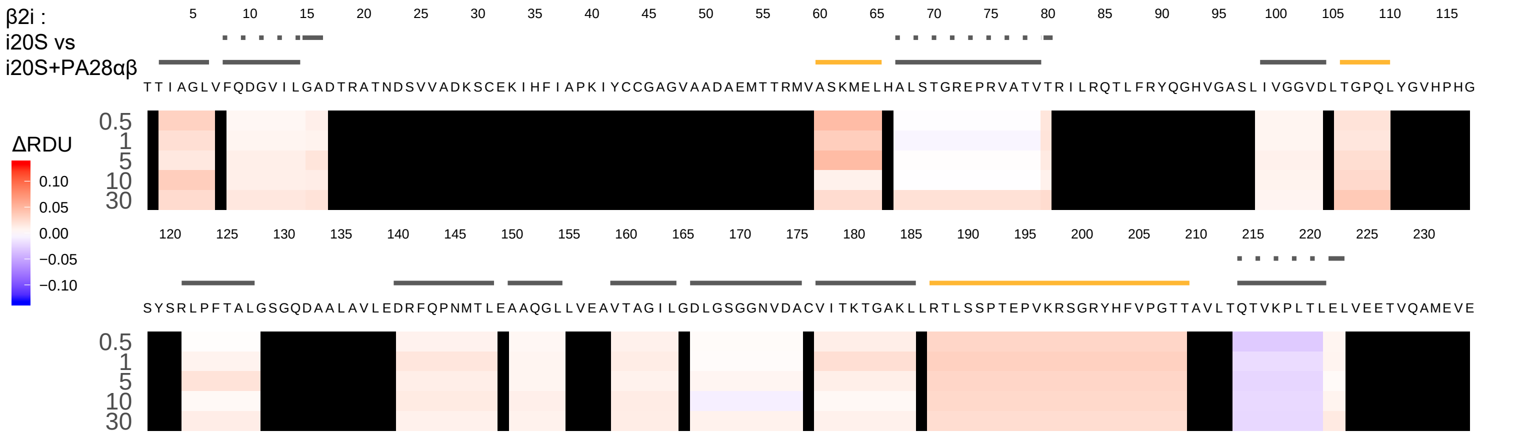
β2i :
i20S vs
i20S+PA28αβ
ΔRDU
0.10
0.05
0.00
−0.05
−0.10
0.5
1
5
10
30
TTIAGLVFQDGVILGADTRATNDSVVADKSCEKIHFIAPKIYCCGAGVAADAEMTTRMVASKMELHALSTGREPRVATVTRILRQTLFRYQGHVGASLIVGGVDLTGPQLYGVHPHG
SYSRLPFTALGSGQDAALAVLEDRFQPNMTLEAAQGLLVEAVTAGILGDLGSGGNVDACVITKTGAKLLRTLSSPTEPVKRSGRYHFVPGTTAVLTQTVKPLTLELVEETVQAMEVE

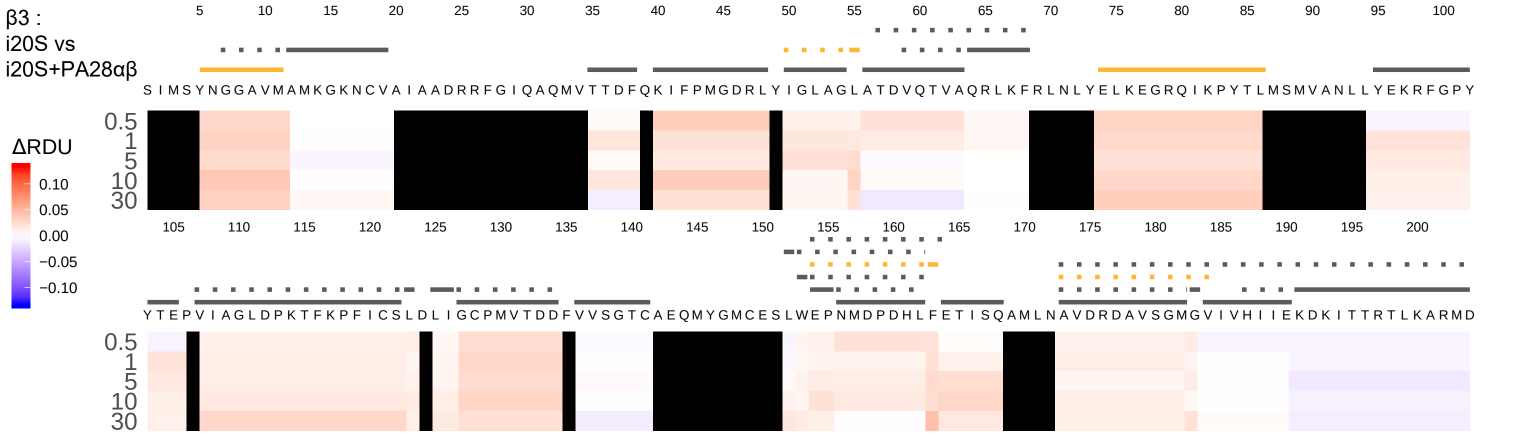

β3 :
i20S vs
i20S+PA28αβ
ΔRDU
0.10
0.05
0.00
−0.05
−0.10
0.5
1
5
10
30

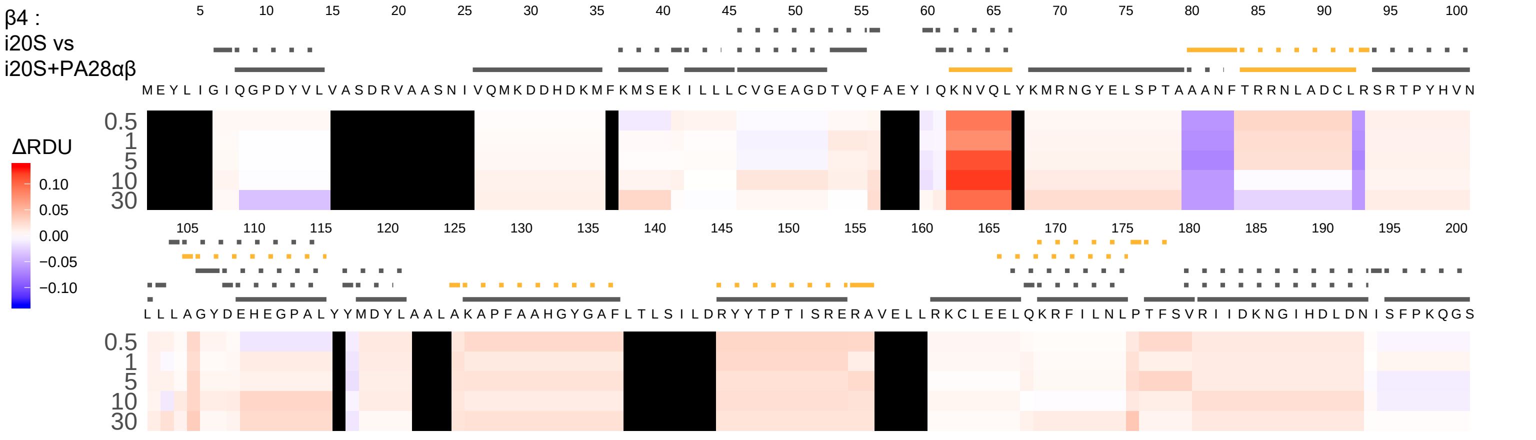
β4 :
i20S vs
i20S+PA28αβ
5 10 15 20 25 30 35 40 45 50 55 60 65 70 75 80 85 90 95 100
MEYLIGIQGPDYVLVASDRVAASNIVQMKDDHDKMFKMSEKILLLCVGEAGDTVQFAEYIQKNVQLYKMRNGYELSPTAAANFTRRNLADCLRSRTPYHVN
ΔRDU
0.10
0.05
0.00
−0.05
−0.10
0.5
1
5
10
30
105 110 115 120 125 130 135 140 145 150 155 160 165 170 175 180 185 190 195 200
LLLAGYDEHEGPALYYMDYLAALAKAPFAAHGYGAFLTLSILDRYYTPTISRERAVELLRKCLEELQKRFILNLPTFSVRIIDKNGIHDLDNISFPKQGS
0.5
1
5
10
30

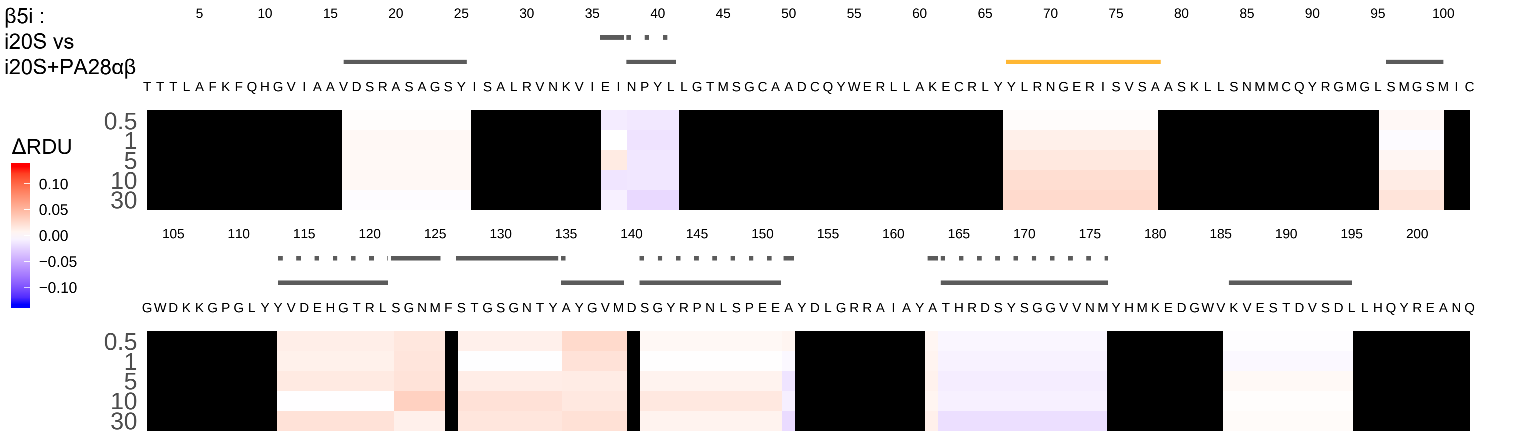

β5i :
i20S vs
i20S+PA28αβ
ΔRDU
0.10
0.05
0.00
−0.05
−0.10
0.5
1
5
10
30
TTTLAFKFQHGVIAAVDSRASAGSYISALRVNKVIEINPYLLGTMSGCAADCQYWERLLAKECRLYYLRNGERISVSAASKLLSNMMCQYRGMGLSMGSMIC
GWDKKGPGLYYVDEHGTRLSGNMFSTGSGNTYAYGVMDSGYRPNLSPEEAYDLGRRAIAYATHRDSYSGGVVNMYHMKEDGWVKVESTDVSDLLHQYREANQ

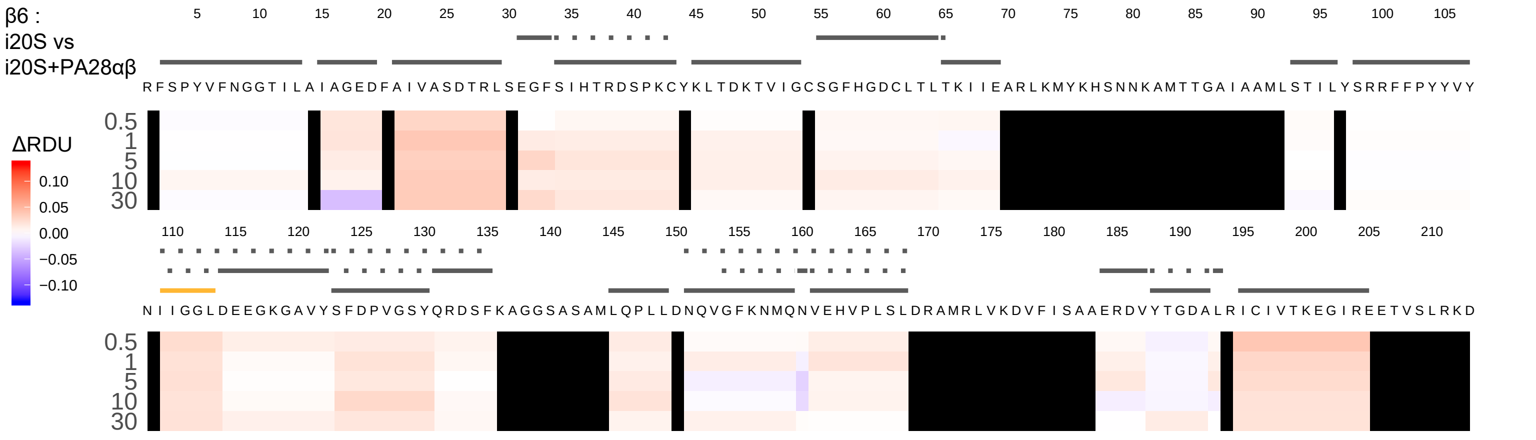

β6 :
i20S vs
i20S+PA28αβ
ΔRDU
0.10
0.05
0.00
−0.05
−0.10
0.5
1
5
10
30
RFSPYVFNGGTILAIAGEDFAIVASDTRLSEGFSIHTRDSPKCYKLTDKTVIGCSGFHGDCLTLTKIIEARLKMYKHSNNKAMTTGAIAAMLSTILYSRRFFPYYVY
NIIGGLDEEGKGAVYSFDPVGSYQRDSFKAGGSASAMLQPLLDNQVGFKNMQNVEHVPLSLDRAMRLVKDVFISAAERDVYTGDALRICIVTKEGIREETVSLRKD

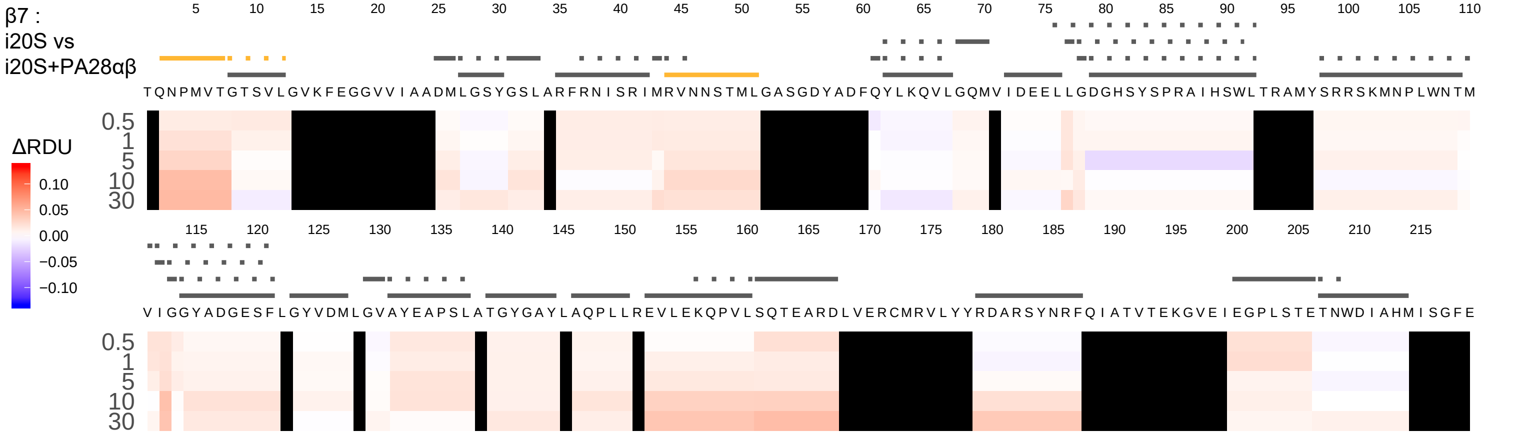
β7 :
i20S vs
i20S+PA28αβ
5 10 15 20 25 30 35 40 45 50 55 60 65 70 75 80 85 90 95 100 105 110
TQNPMVTGTSVLGVKFEGGVVIAADMLGSYGSLARFRNISRIMRVNNSTMLGASGDYADFQYLKQVLGQMVIDEELLGDGHSYSPRAIHSWLTRAMYSRRSKMNPLWNTM
0.5
1
5
10
30
ΔRDU
0.10
0.05
0.00
−0.05
−0.10
115 120 125 130 135 140 145 150 155 160 165 170 175 180 185 190 195 200 205 210 215
VIGGYADGESFLGYVDMLGVAYEAPSLATGYGAYLAQPLLREVLEKQPVLSQTEARDLVERCMRVLYYRDARSYNRFQIATVTEKGVEIEGPLSTETNWDIAHMISGFE

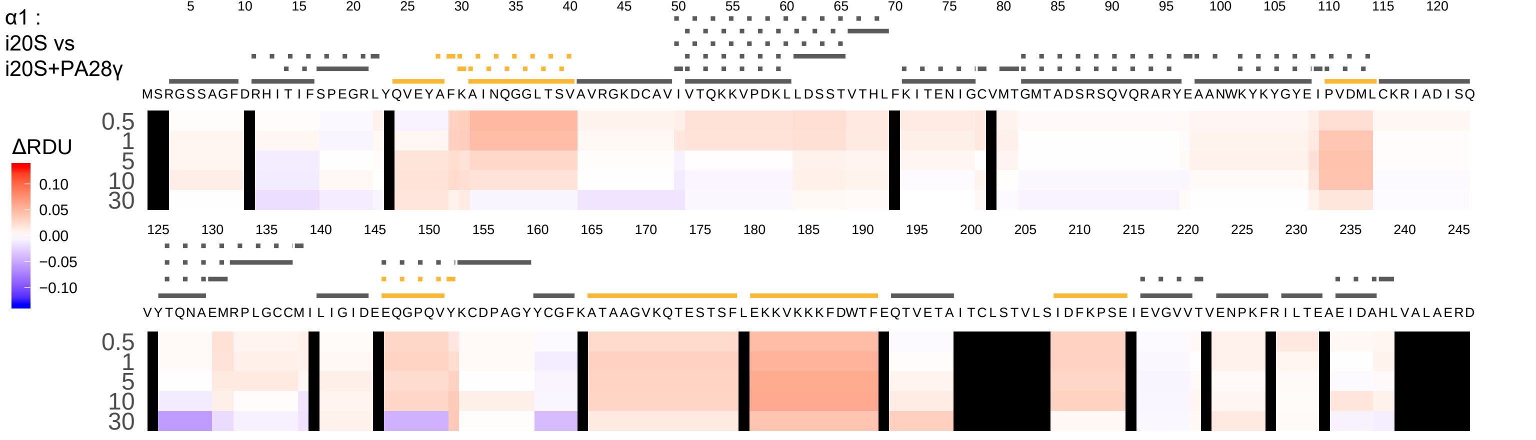
α1 :
i20S vs
i20S+PA28γ
MSRGSSAGFDRHITIFSPEGRLYQVEYAFKAINQGGLTSVAVRGKDCAVIVTQKKVPDKLLDSSTVTHLFKITENIGCVMTGMTADSRSQVQRARYEAANWKYKYGYEIPVDMLCKRIADISQ
VYTQNAEMRPLGCCMILIGIDEEQGPQVYKCDPAGYYCGFKATAAGVKQTESTSFLEKKVKKKFDWTFEQTVETAITCLSTVLSIDFKPSEIEVGVVTVENPKFRILTEAEIDAHLVALAERD
ΔRDU
0.10
0.05
0.00
−0.05
−0.10
0.5
1
5
10
30

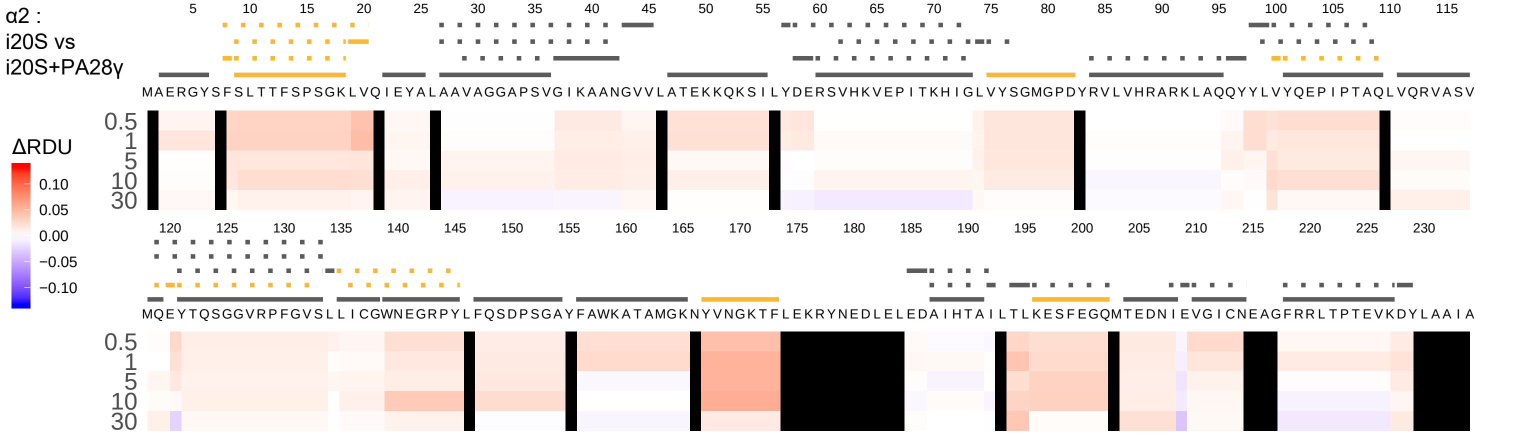
α2 :
i20S vs
i20S+PA28γ
MAERGYSFSLTTFSPSGKLVQIEYALAAVAGGAPSVGIKAANGVVLATEKKQKSILYDERSVHKVEPITKHIGLVVYSGMGPDYRVLVHRARKLAQQYYLVYQEPIPTAQLVQRVASV
MQEYTQSGGVRPFGVSLLICGWNEGRPYLFQSDPSGAYFAWKATAMGKNYVNGKTFLEKRYNEDLELEDAIHTAILTLKESFEGQMTEDNIEVGICNEAGFRRLTPTEVKDYLAAIA
ΔRDU
0.10
0.05
0.00
−0.05
−0.10
0.5
1
5
10
30

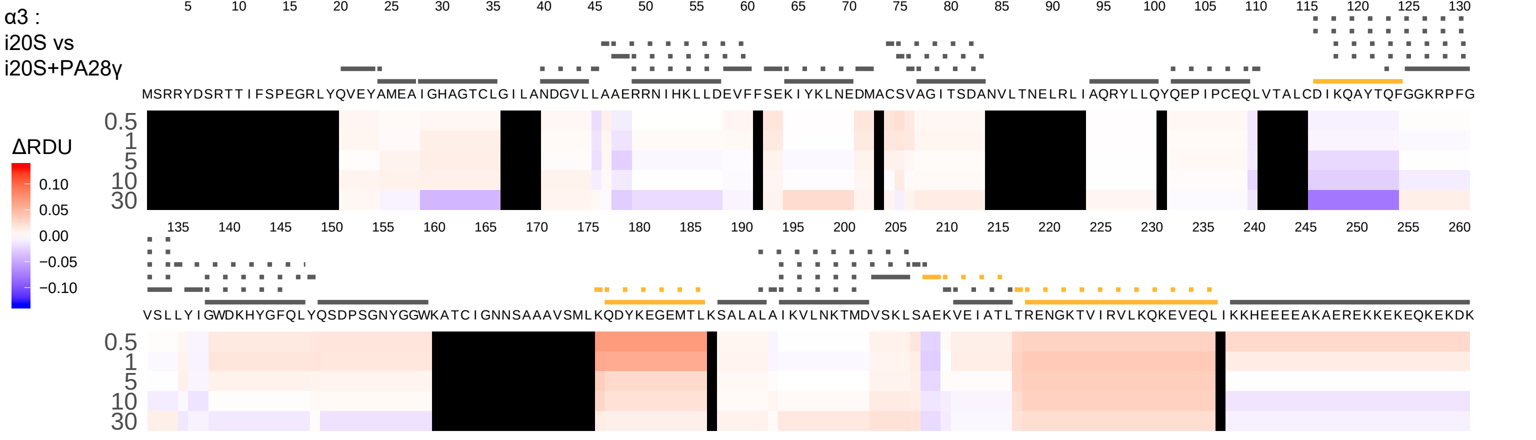
α3 :
i20S vs
i20S+PA28γ
ΔRDU
0.10
0.05
0.00
−0.05
−0.10
0.5
1
5
10
30
MSRRYDSRTTIFSPEGRLYQVEYAMEAIGHAGTCLGILANDGVLLAAERRNIHKLLDEVFFSEKIYKLNEDMACSVAGITSDANVLTNELRLIAQRYLLQYQEPIPCEQLVTALCDIKQAYTQFGGKRPFG
VSLLYIGWDKHYGFQLYQSDPSGNYGGWKATCIGNNSAAAVSMLKQDYKEGEMTLKSALALAIKVLNKTMDVSKLSAEKVEIATLTRENGKTVIRVLKQKEVEQLIKKHEEEEAKAEREKKEKEQKEKDK

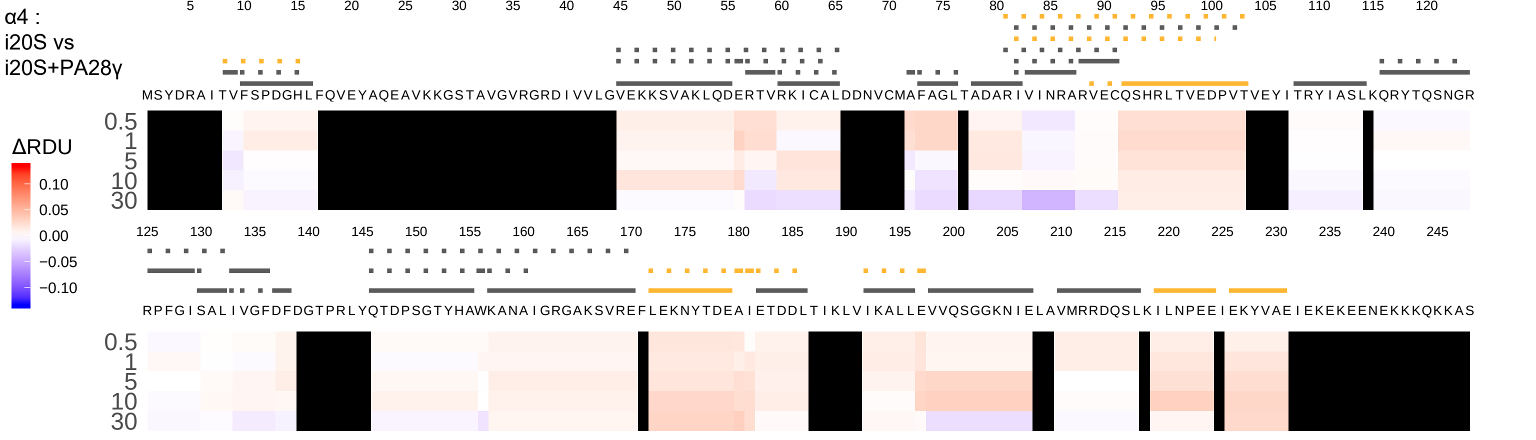
α4 :
i20S vs
i20S+PA28γ
ΔRDU
0.10
0.05
0.00
−0.05
−0.10
0.5
1
5
10
30
MSYDRAITVFSPDGHLFQVEYAQEAVKKGSTAVGVRGRDIVVLGVEKKSVAKLQDERTVRKICALDDNVCMAFAGLTADARIVINRARVECQSHRLTVEDPVTVEYITRYIASLKQRYTQSNGR
RPFGISALIVGFDFDGTPRLYQTDPSGTYHAWKANAIGRGAKSVREFLEKNYTDEAIETDDLTIKLVIKALLEVVQSGGKNIELAVMRRDQSLKILNPEEIEKYVAEIEKEKEENEKKKQKKAS

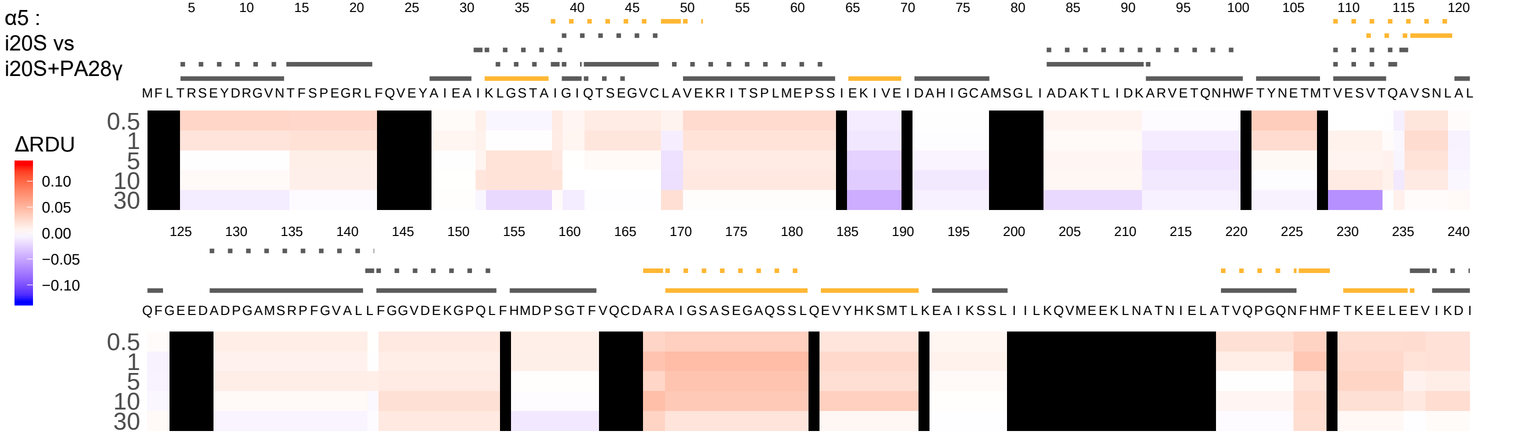
α5 :
i20S vs
i20S+PA28γ
5 10 15 20 25 30 35 40 45 50 55 60 65 70 75 80 85 90 95 100 105 110 115 120
MFLTRSEYDRGVNTFSPEGRLFQVEYAIEAIKLGSTAIGIQTSEGVCLAVEKRITSPLMEPSSIEKIVEIDAHIGCAMSGLIADAKTLIDKARVETQNHWFTYNETMTVESVTQAVSNLAL
0.5
1
5
10
30
ΔRDU
0.10
0.05
0.00
−0.05
−0.10
125 130 135 140 145 150 155 160 165 170 175 180 185 190 195 200 205 210 215 220 225 230 235 240
QFGEEDADPGAMSRPFGVALLFGGVDEKGPQLFHMDPSGTFVQCDARAIGSASEGAQSSLQEVYHKSMTLKEAIKSSLIIILKQVMEEKLNATNIELATVQPGQNFHMFTKEELEEVIKDI
0.5
1
5
10
30

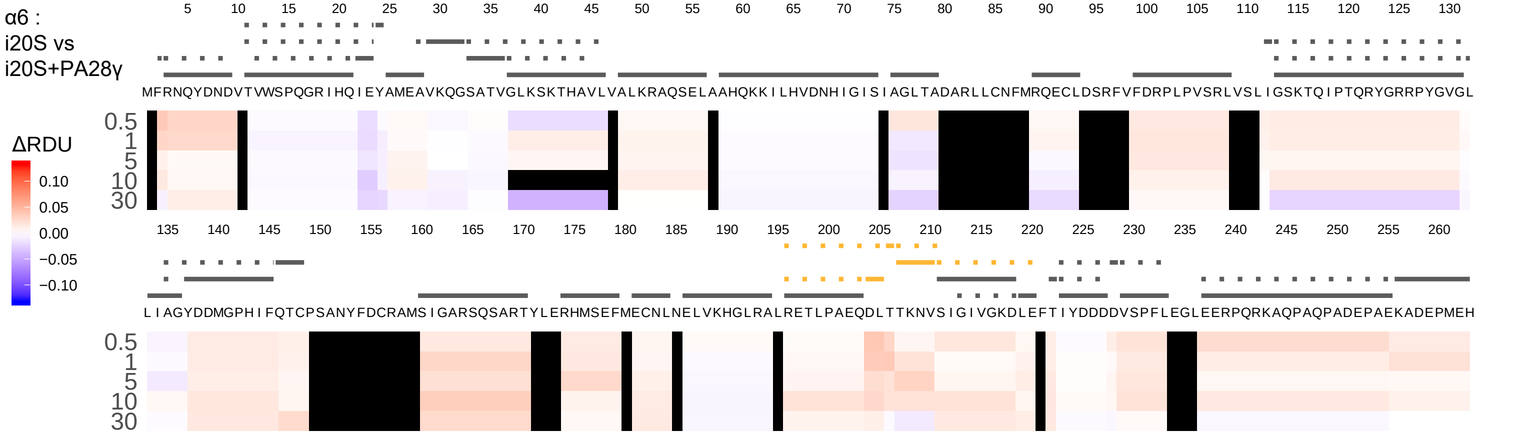

α6 :
i20S vs
i20S+PA28γ
ΔRDU
0.10
0.05
0.00
−0.05
−0.10
0.5
1
5
10
30
MFRNQYDNDVTVWSPQGRIHQIEYAMEAVKQGSATVGLKSKTHAVLVALKRAQSELAAHQKKILHVDNHIGISIAGLTADARLLCNFMRQECLDSRFVFDRPLPVSRLVSLIGSKTQIPTQRYGRRPYGVGL
LIAGYDDMGPHIFQTCPSANYFDCRAMSIGARSQSARTYLERHMSEFMECNLNELVKHGLRALRETLPAEQDLTTKNVSIGIVGKDLEFTIYDDDDVSPFLEGLEERPQRKAQPAQPADEPAEKADEPMEH

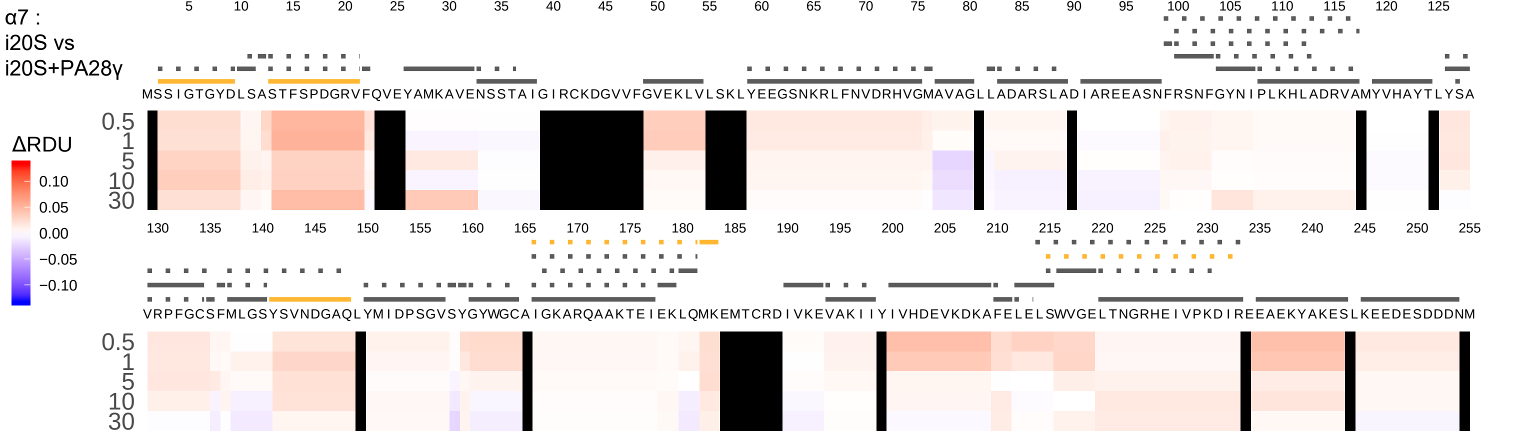

α7 :
i20S vs
i20S+PA28γ
ΔRDU
0.10
0.05
0.00
−0.05
−0.10
MSSIGTGYDLSASTFSPDGRVFQVEYAMKAVENSSTAIGIRCKDGVVFGVEKLVLSKLYEEGSNKRLFNVDRHVGMAVAGLLADARSLADIAREEASNFRSNFGYNIPLKHLADRVAMYVHAYTLYSA
VRPFGCSFMLGSYSVNDGAQLYMIDPSGVSYGYWGCAIGKARQAAKTEIEKLQMKEMTCRDIVKEVAKIIYIVHDEVKDKAFELELSWVGELTNGRHEIVPKDIREEAEKYAKESLKEEDESDDDNM
0.5
1
5
10
30

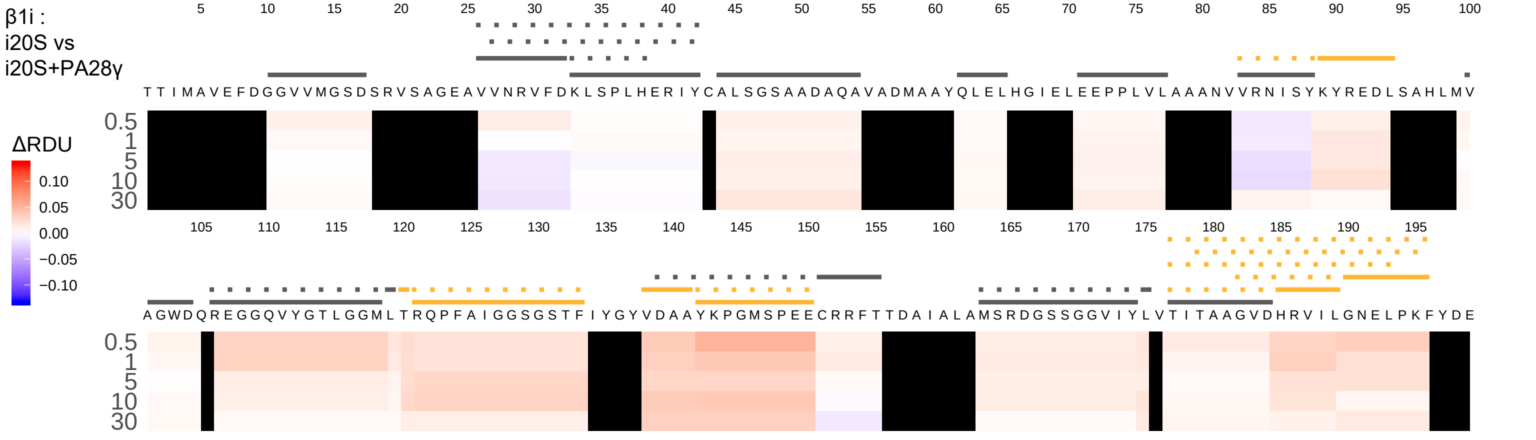
β1i :
i20S vs
i20S+PA28γ
ΔRDU
0.10
0.05
0.00
−0.05
−0.10
T T I M A V E F D G G V V M G S D S R V S A G E A V V N R V F D K L S P L H E R I Y C A L S G S A A D A Q A V A D M A A Y Q L E L H G I E L E E P P L V L A A A N V V R N I S Y K Y R E D L S A H L M V
A G W D Q R E G G Q V Y G T L G G M L T R Q P F A I G G S G S T F I Y G Y V D A A Y K P G M S P E E C R R F T T D A I A L A M S R D G S S G G V I Y L V T I T A A G V D H R V I L G N E L P K F Y D E
0.5
1
5
10
30

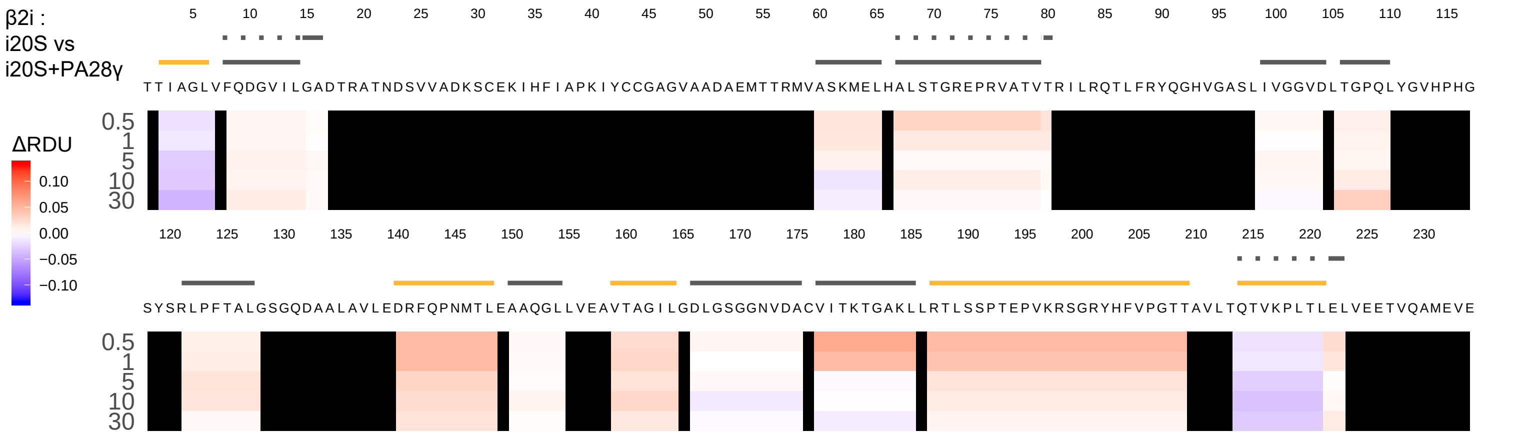
β2i :
i20S vs
i20S+PA28γ
ΔRDU
0.10
0.05
0.00
−0.05
−0.10
TTIAGLVFQDGVILGADTRATNDSVVADKSCEKIHFIAPKIYCCGAGVAADAEMTTRMVASKMELHALSTGREPRVATVTRILRQTLFRYQGHVGASLIVGGVDLTGPQLYGVHPHG
SYSRLPFTALGSGQDAALAVLEDRFQPNMTLEAAQGLLVEAVTAGILGDLGSGGNVDACVITKTGAKLLRTLSSPTEPVKRSGRYHFVPGTTAVLTQTVKPLTLELVEETVQAMEVE
0.5
1
5
10
30

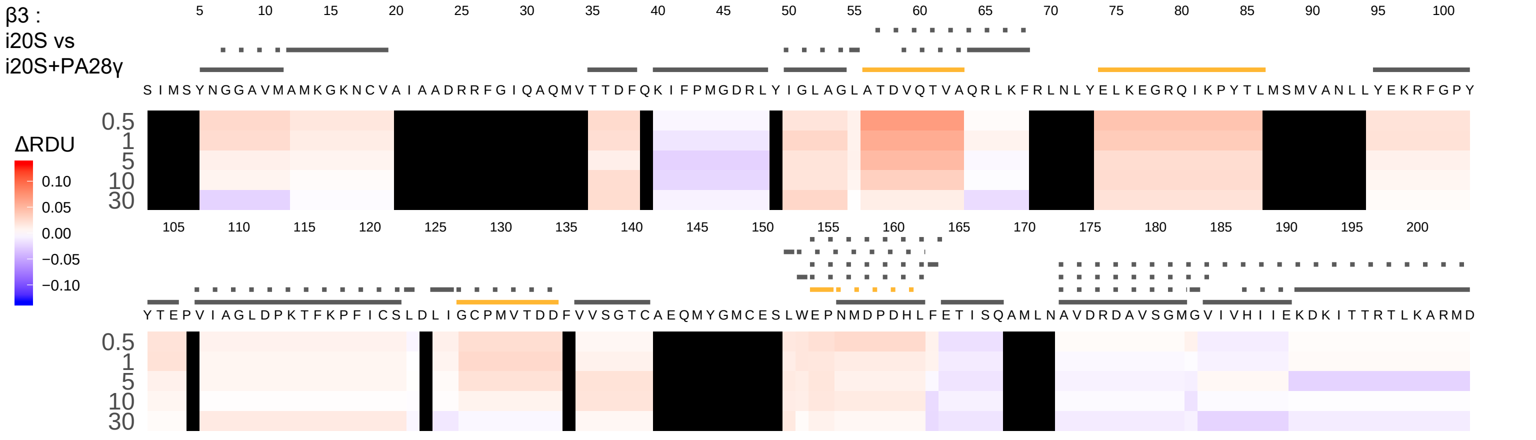
β3 :
i20S vs
i20S+PA28γ
ΔRDU
0.10
0.05
0.00
−0.05
−0.10
0.5
1
5
10
30
SIMSYNGGAVMAMKGKNCVAIAADRRFGIQAQMVTTDFQKIFPMGDRLYIGLAGLATDVQTVAQRLKFRLNLYELKEGRQIKPYTLMSMVANLLYEKRFGPY
YTEPVIAGLDPKTFKPFICSLDLIGCPMVTDDFVVSGTCAEQMYGMCESLWEPNMDPDHLFETISQAMLNAVDRDAVSGMGVIVHIIEKDKITTRTLKARMD

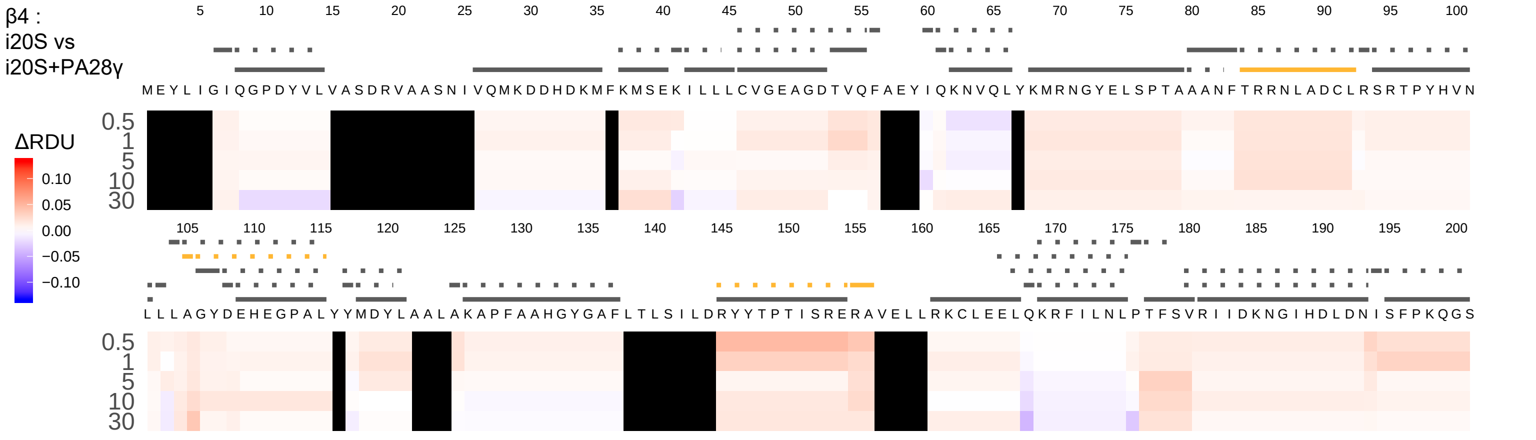
β4 :
i20S vs
i20S+PA28γ
ΔRDU
0.10
0.05
0.00
−0.05
−0.10
5 10 15 20 25 30 35 40 45 50 55 60 65 70 75 80 85 90 95 100
MEYLIGIQGPDYVLVASDRVAASNIVQMKDDHDKMFKMSEKILLLCVGEAGDTVQFAEYIQKNVQLYKMRNGYELSPTAAANFTRRNLADCLRSRTPYHVN
0.5
1
5
10
30
105 110 115 120 125 130 135 140 145 150 155 160 165 170 175 180 185 190 195 200
LLLAGYDEHEGPALYYMDYLAALAKAPFAAHGYGAFLTLSILDRYYTPTISRERAVELLRKCLEELQKRFILNLPTFSVRIIDKNGIHDLDNISFPKQGS
0.5
1
5
10
30

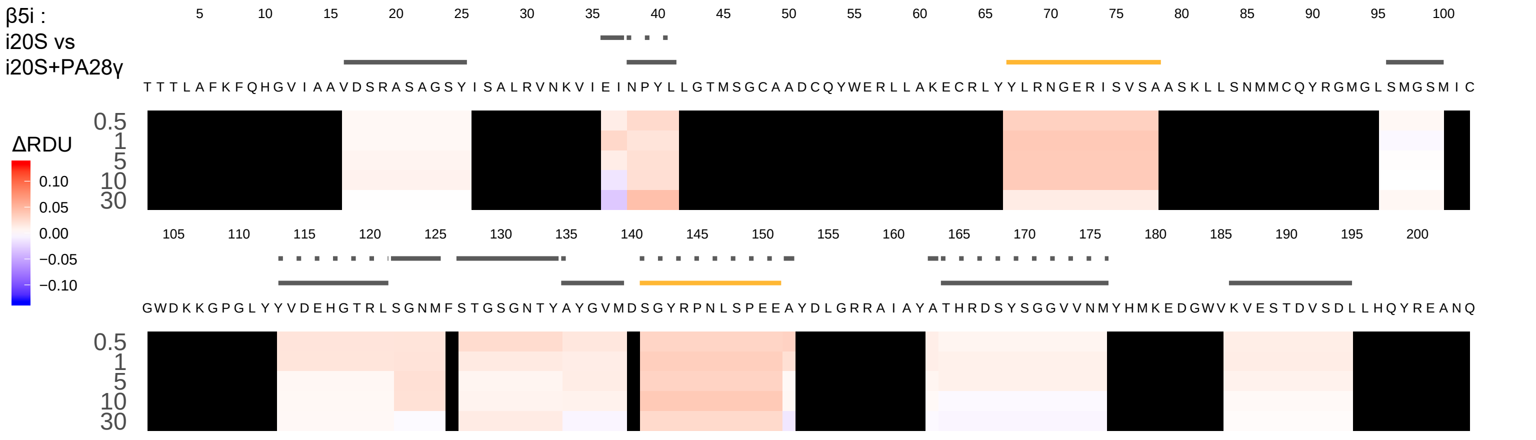
β5i :
i20S vs
i20S+PA28γ
ΔRDU
0.10
0.05
0.00
−0.05
−0.10
0.5
1
5
10
30
5 10 15 20 25 30 35 40 45 50 55 60 65 70 75 80 85 90 95 100
T T T L A F K F Q H G V I A A V D S R A S A G S Y I S A L R V N K V I E I N P Y L L G T M S G C A A D C Q Y W E R L L A K E C R L Y Y L R N G E R I S V S A A S K L L S N M M C Q Y R G M G L S M G S M I C
105 110 115 120 125 130 135 140 145 150 155 160 165 170 175 180 185 190 195 200
G W D K K G P G L Y Y V D E H G T R L S G N M F S T G S G N T Y A Y G V M D S G Y R P N L S P E E A Y D L G R R A I A Y A T H R D S Y S G G V V N M Y H M K E D G W V K V E S T D V S D L L H Q Y R E A N Q

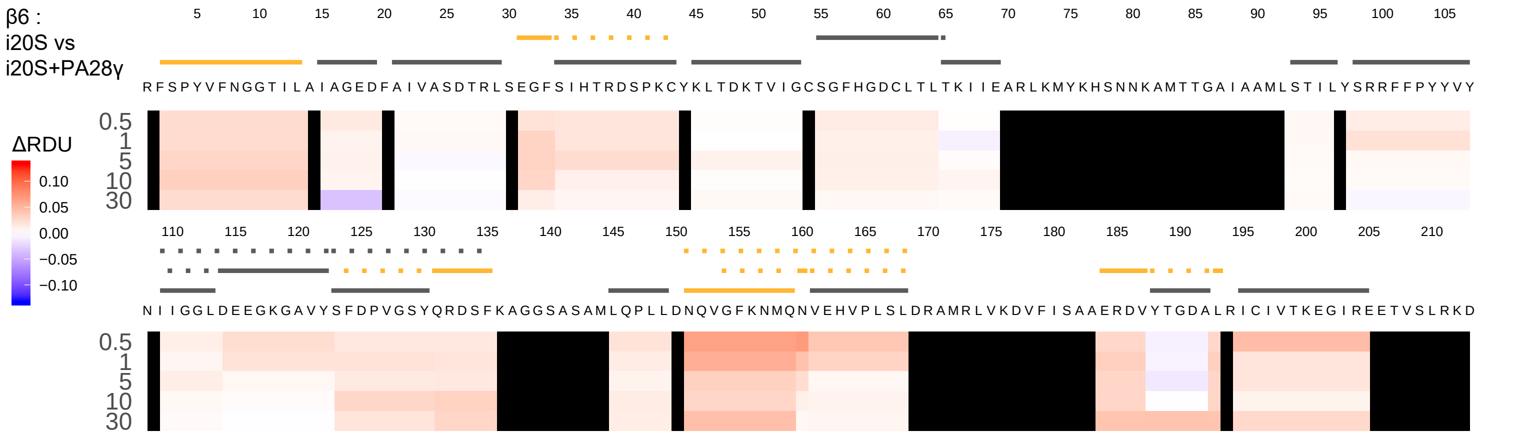

β6 :
i20S vs
i20S+PA28γ
ΔRDU
0.10
0.05
0.00
−0.05
−0.10
0.5
1
5
10
30
RFSPYVFNGGTILAIAGEDFAIVASDTRLSEGFSIHTRDSPKCYKLTDKTVIGCSGFHGDCLTLTKIIEARLKMYKHSNNKAMTTGAIAAMLSTILYSRRFFPYYVY
NIIGGLDEEGKGAVYSFDPVGSYQRDSFKAGGSASAMLQPLLDNQVGFKNMQNVEHVPLSLDRAMRLVKDVFISAAERDVYTGDALRICIVTKEGIREETVSLRKD

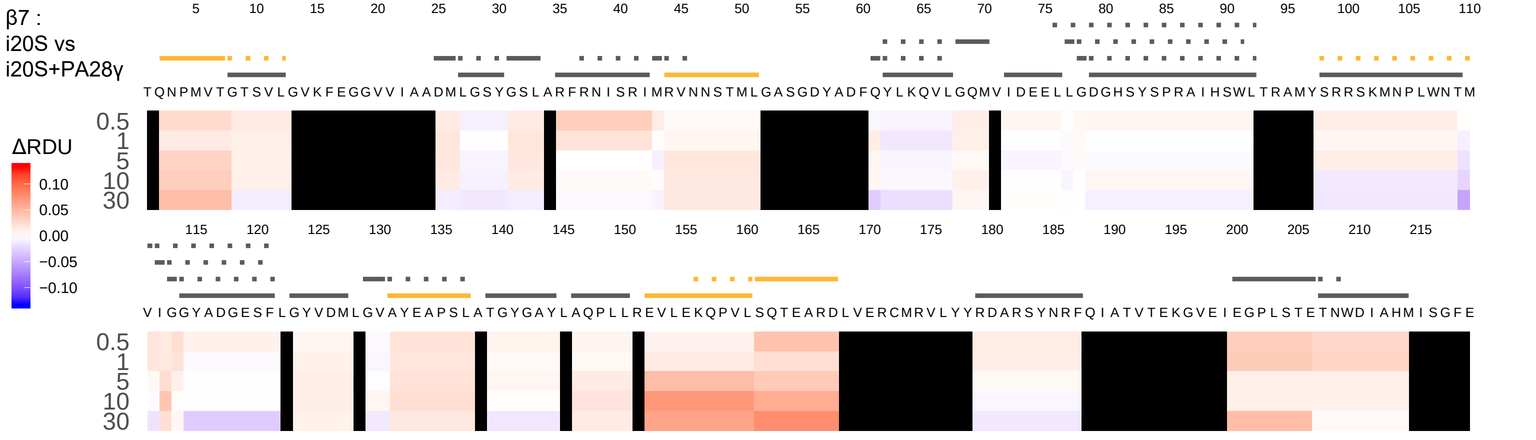
β7 :
i20S vs
i20S+PA28γ
ΔRDU
0.10
0.05
0.00
−0.05
−0.10
0.5
1
5
10
30
TQNPMVTGTSVLGVKFEGGVVIAADMLGSYGSLARFRNISRIMRVNNSTMLGASGDYADFQYLKQVLGQMVIDEELLGDGHSYSPRAIHSWLTRAMYSRRSKMNPLWNTM
VIGGYADGESFLGYVDMLGVAYEAPSLATGYGAYLAQPLLREVLEKQPVLSQTEARDLVERCMRVLYYRDARSYNRFQIATVTEKGVEIEGPLSTETNWDIAHMISGFE
