# Supplementary material for: Conformational maps of human 20S proteasomes reveal PA28- and immuno-dependent inter-ring crosstalks
Source: Nat Commun. 2020 Dec 1;11:6140. doi: 10.1038/s41467-020-19934-z (PMC7708635; doi:10.1038/s41467-020-19934-z)
Supplement: Supplementary file 8 — Dataset 6 [file 41467_2020_19934_MOESM8_ESM.pdf]

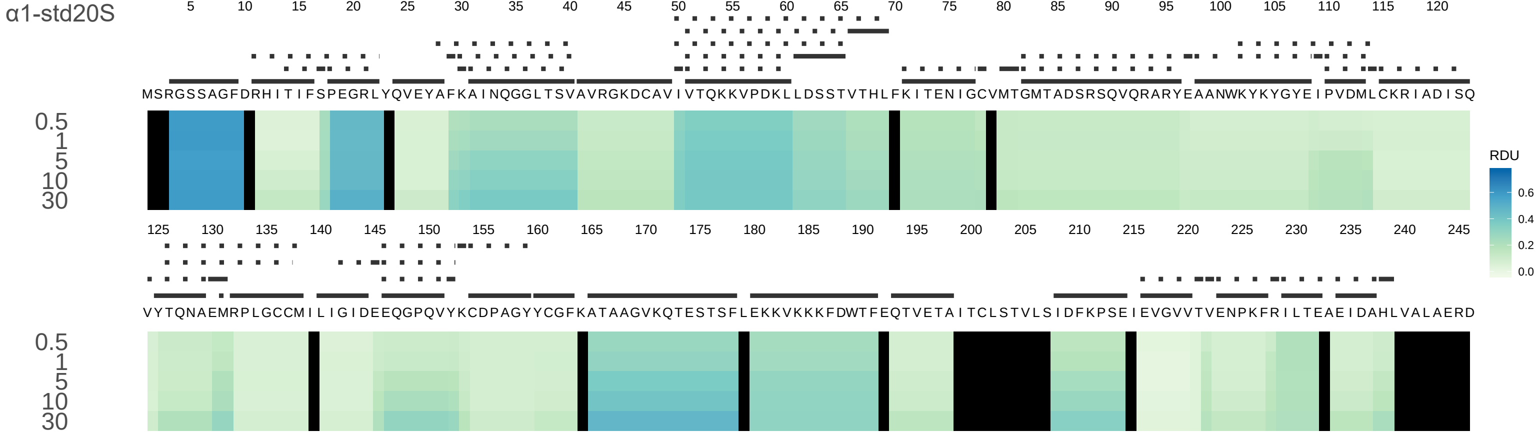
α1-std20S
MSRGSSAGFDRHITIFSPEGRLYQVEYAFKAINQGGLTSVAVRGKDCAVIVTQKKVPDKLLDSSTVTHLFKITENIGCVMTGMTADSRSQVQRARYEAANWKYKYGYEIPVDMLCKRIADISQ
VYTQNAEMRPLGCCMILIGIDEEQGPQVYKCDPAGYYCGFKATAAGVKQTESTSFLEKKVKKKFDWTFEQTVETAITCLSTVLSIDFKPSEIEVGVVTVENPKFRILTEAEIDAHLVALAERD
0.5
1
5
10
30
RDU
0.6
0.4
0.2
0.0

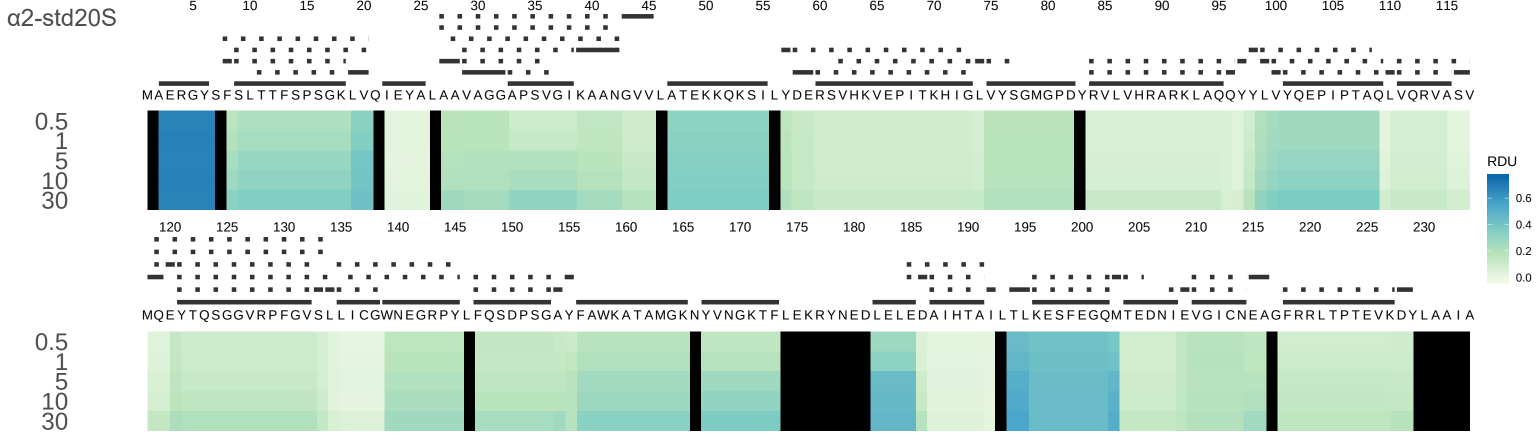

α2-std20S
MAERGYSFSLTTFSPSGKLVQIEYALAAVAGGAPSVGIKAANGVVLATEKKQKSILYDERSVHKVEPITKHIGLVYSGMGPDYRVLVHRARKLAQQYYLVYQEPIPTAQLVQRVASV
MQEYTQSGGVRPFGVSLLICGWNEGRPYLFQSDPSGAYFAWKATAMGKNYVNGKTFLEKRYNEDLELEDAIHTAILTLKESFEGQMTEDNIEVGICNEAGFRRLTPTEVKDYLAAIA
0.5
1
5
10
30
RDU
0.6
0.4
0.2
0.0

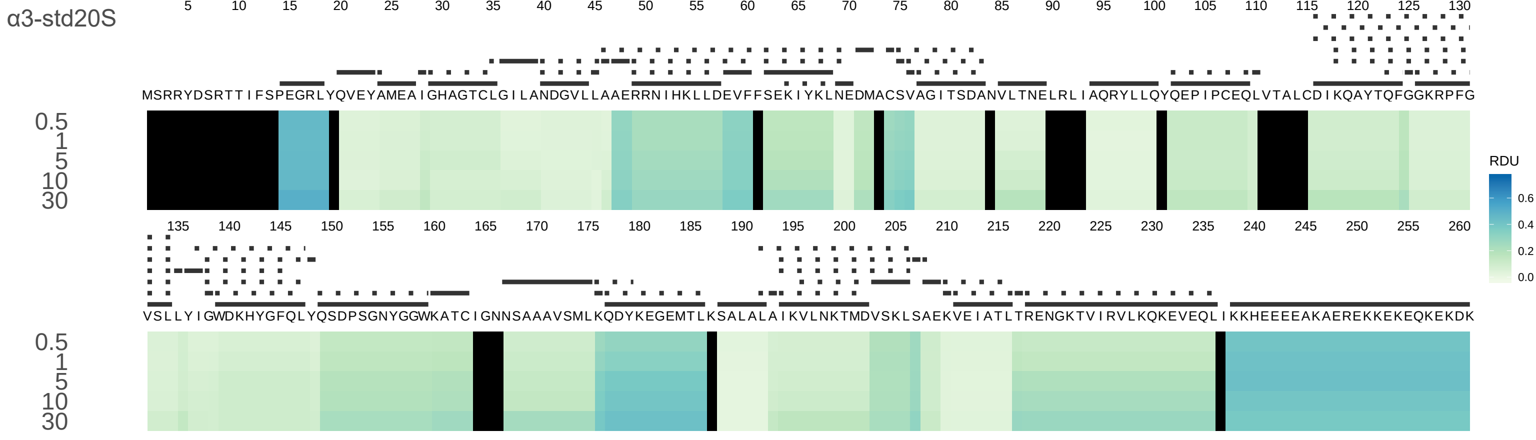

α3-std20S
5 10 15 20 25 30 35 40 45 50 55 60 65 70 75 80 85 90 95 100 105 110 115 120 125 130
MSRRYDSRTTIFSPEGRLYQVEYAMEAIGHAGTCLGILANDGVLLAAERRNIHKLLDEVFFSEKIYKLNEDMACSVAGITSDANVLTNELRLIAQRYLLQYQEPIPCEQLVTALCDIKQAYTQFGGKRPFG
0.5
1
5
10
30
135 140 145 150 155 160 165 170 175 180 185 190 195 200 205 210 215 220 225 230 235 240 245 250 255 260
VSLLYIGWDKHYGFQLYQSDPSGNYGGWKATCIGNNSAAAVSMLKQDYKEGEMTLKSALALAIKVLNKTMDVSKLSAEKVEIATLTRENGKTVIRVLKQKEVEQLIKKHEEEEAKAEREKKEKEQKEKDK
0.5
1
5
10
30
RDU
0.6
0.4
0.2
0.0

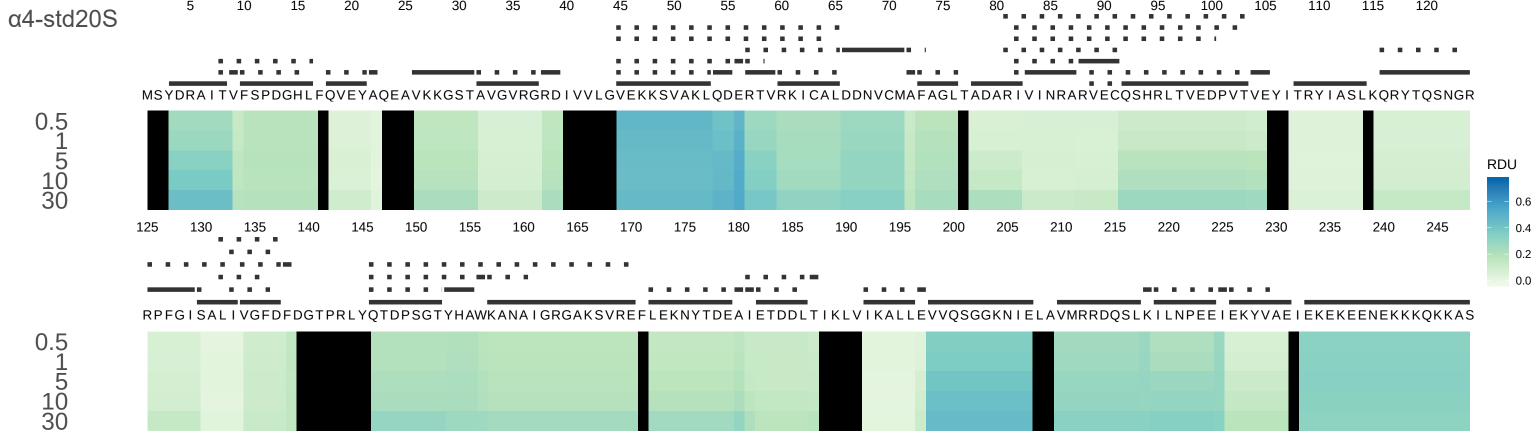
α4-std20S
MSYDRAITVFSPDGHLFQVEYAQEAVKKGSTAVGVRGRDIVVLGVEKKSVAKLQDERTVRKICALDDNVCMAFAGLTADARIVINRARVECQSHRLTVEDPVTVEYITRYIASLKQRYTQSNGR
RPFGISALIVGFDFDGTPRLYQTDPSGTYHAWKANAIGRGAKSVREFLEKNYTDEAIETDDLTIKLVIKALLEVVQSGGKNIELAVMRRDQSLKILNPEEIEKYVAEIEKEKEENEKKKQKKAS
0.5
1
5
10
30
RDU
0.6
0.4
0.2
0.0

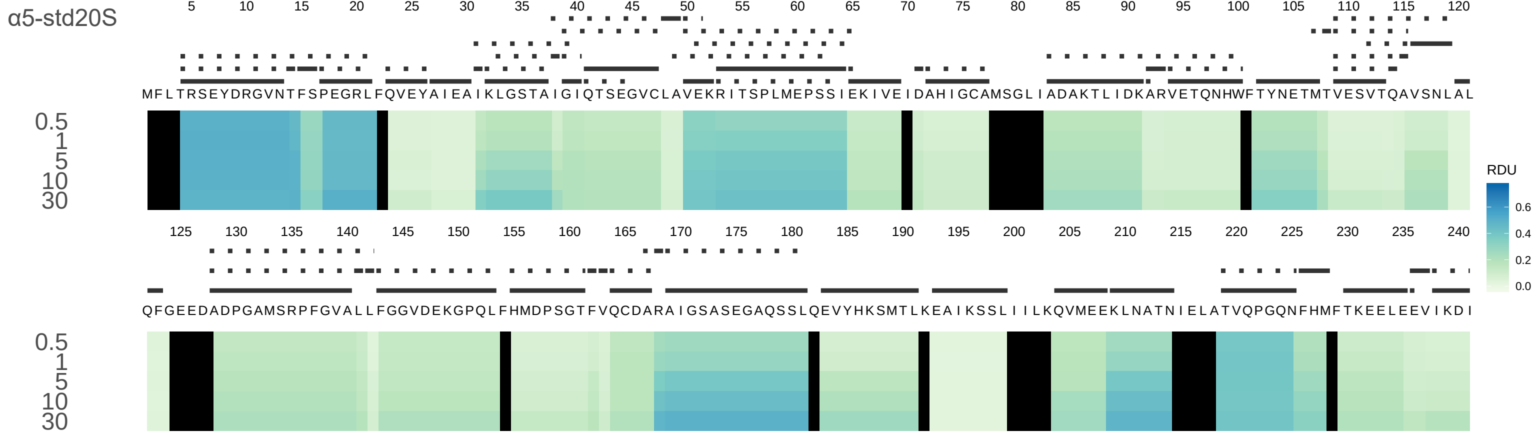
α5-std20S
MFLTRSEYDRGVNTFSPEGRLFQVEYAIEAIKLGSTAIGIQTSEGVCLAVEKRITSPLMEPSSIEKIVEIDAHIGCAMSGLIADAKTLIDKARVETQNHWFTYNETMTVESVTQAVSNLAL
QFGEEDADPGAMSRPFGVALLFGGVDEKGPQLFHMDPSGTFVQCDARAIGSASEGAQSSLQEVYHKSMTLKEAIKSSLIIILKQVMEEKLNATNIELATVQPGQNFHMFTKEELEEVIKDI
0.5
1
5
10
30
RDU
0.6
0.4
0.2
0.0

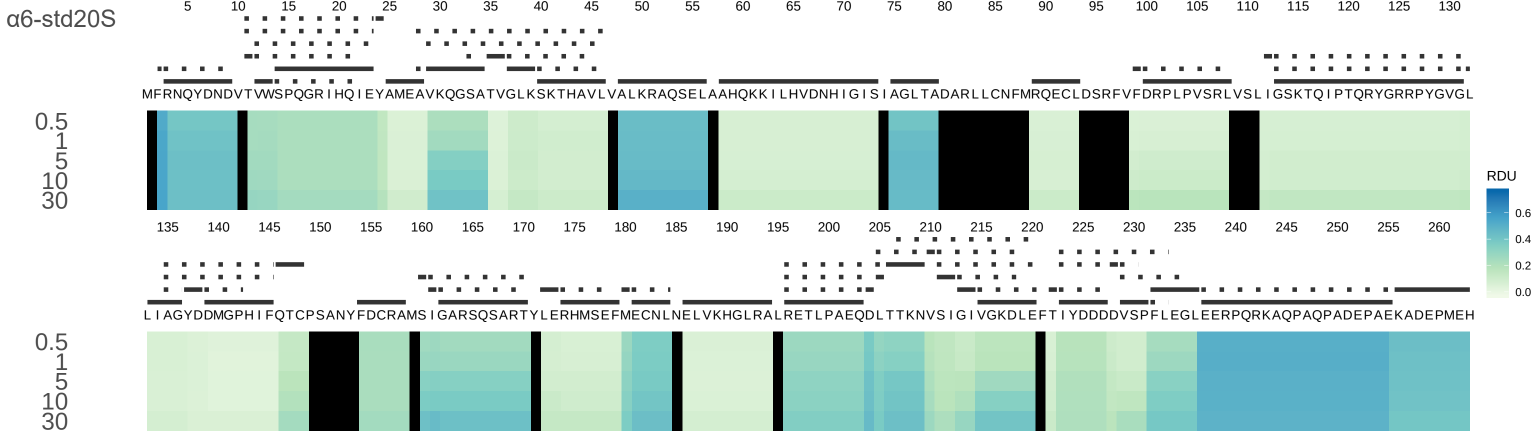
α6-std20S
5 10 15 20 25 30 35 40 45 50 55 60 65 70 75 80 85 90 95 100 105 110 115 120 125 130
MFRNQYDNDVTVWSPQGRIHQIEYAMEAVKQGSATVGLKSKTHAVLVALKRAQSELAAHQKKILHVDNHIGISIAGLTADARLLCNFMRQECLDSRFVFDRPLPVSRLVSLIGSKTQIPTQRYGRRPYGVGL
0.5
1
5
10
30
135 140 145 150 155 160 165 170 175 180 185 190 195 200 205 210 215 220 225 230 235 240 245 250 255 260
LIAGYDDMGPHIFQTCPSANYFDCRAMSIGARSQSARTYLERHMSEFMECNLNELVKHGLRALRETLPAEQDLTTKNVSIGIVGKDLEFTIYDDDDVSPFLEGLEERPQRKAQPAQPADEPAEKADEPMEH
0.5
1
5
10
30
RDU
0.6
0.4
0.2
0.0

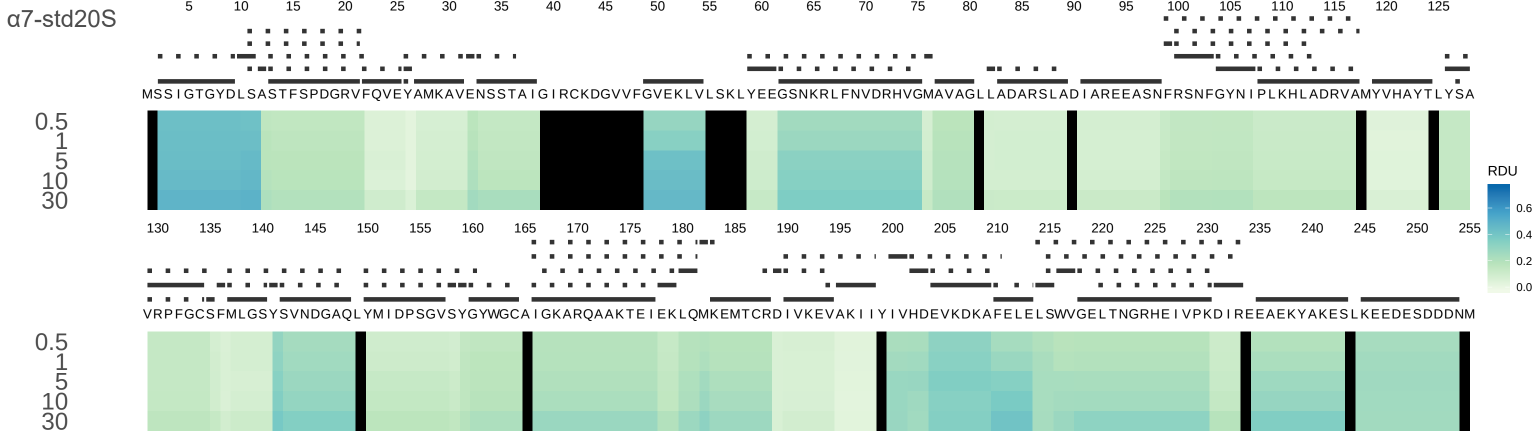
α7-std20S
MSSIGTGYDLSASTFSPDGRVFQVEYAMKAVENSSTAIGIRCKDGVVFGVEKLVLSKLYEEGSNKRLFNVDRHVGMAVAGLLADARSLADIAREEASNFRSNFGYNIPLKHLADRVAMYVHAYTLYSA
VRPFGCSFMLGSYSVNDGAQLYMIDPSGVSYGYWGCAIGKARQAAKTEIEKLQMKEMTCRDIVKEVAKIIYIVHDEVKDKAFELELSWVGELTNGRHEIVPKDIREEAEKYAKESLKEEDESDDDNM
0.5
1
5
10
30
RDU

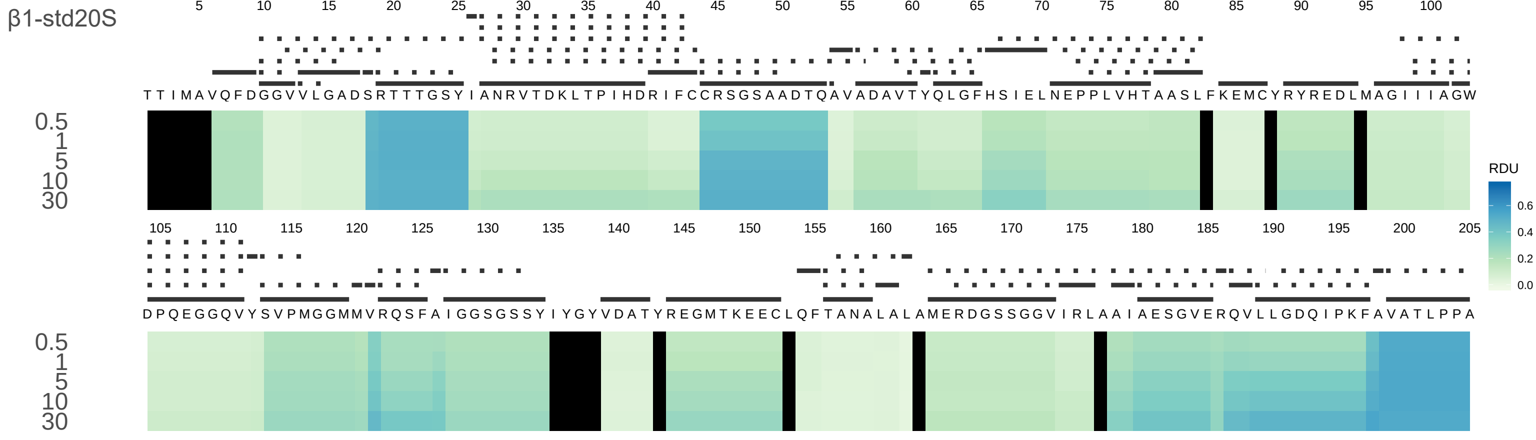
β1-std20S
TTIMAVQFDGGVVLGADSRTTTGSYIANRVTDKLTPIHDRIFCCRSGSAADTQAVADAVTYQLGFHSIELNEPPLVHTAASLFKEMCYRYREDLMAGIIIAGW
DPQEGGQVYSVPMGGMMVRQSFAIGGSGSSYIYGYVDATYREGMTKEECLQFTANALALAMERDGSSGGVIRLAAIAESGVERQVLLGDQIPKFAVATLPPA
0.5
1
5
10
30
RDU
0.6
0.4
0.2
0.0

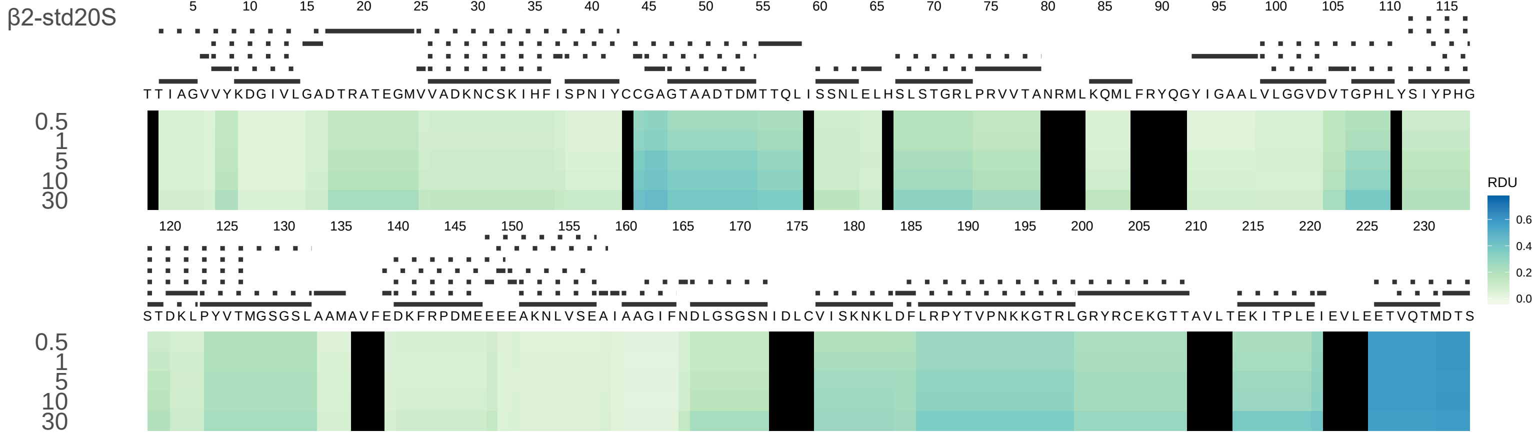

β2-std20S
5 10 15 20 25 30 35 40 45 50 55 60 65 70 75 80 85 90 95 100 105 110 115
T T I A G V V Y K D G I V L G A D T R A T E G M V V A D K N C S K I H F I S P N I Y C C G A G T A A D T D M T T Q L I S S N L E L H S L S T G R L P R V V T A N R M L K Q M L F R Y Q G Y I G A A L V L G G V D V T G P H L Y S I Y P H G
0.5
1
5
10
30
120 125 130 135 140 145 150 155 160 165 170 175 180 185 190 195 200 205 210 215 220 225 230
S T D K L P Y V T M G S G S L A A M A V F E D K F R P D M E E E E A K N L V S E A I A A G I F N D L G S G S N I D L C V I S K N K L D F L R P Y T V P N K K G T R L G R Y R C E K G T T A V L T E K I T P L E I E V L E E T V Q T M D T S
0.5
1
5
10
30
RDU
0.6
0.4
0.2
0.0

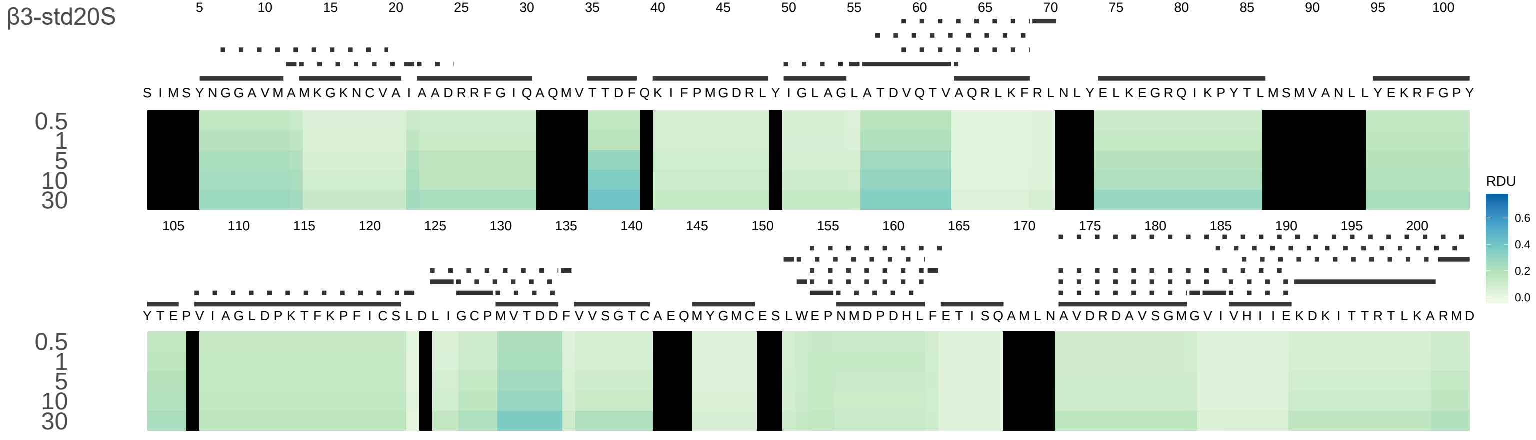
β3-std20S
5 10 15 20 25 30 35 40 45 50 55 60 65 70 75 80 85 90 95 100
S I M S Y N G G A V M A M K G K N C V A I A A D R R F G I Q A Q M V T T D F Q K I F P M G D R L Y I G L A G L A T D V Q T T V A Q R L K F R L N L Y E L K E G R Q I K P Y T L M S M V A N L L Y E K R F G P Y
0.5
1
5
10
30
105 110 115 120 125 130 135 140 145 150 155 160 165 170 175 180 185 190 195 200
Y T E P V I A G L D P K T F K P F I C S L D L I G C P M V T D D F V V S G T C A E Q M Y G M C E S L W E P N M D P D H L F E T I S Q A M L N A V D R D A V S G M G V I V H I I E K D K I T T R T L K A R M D
0.5
1
5
10
30
RDU
0.6
0.4
0.2
0.0

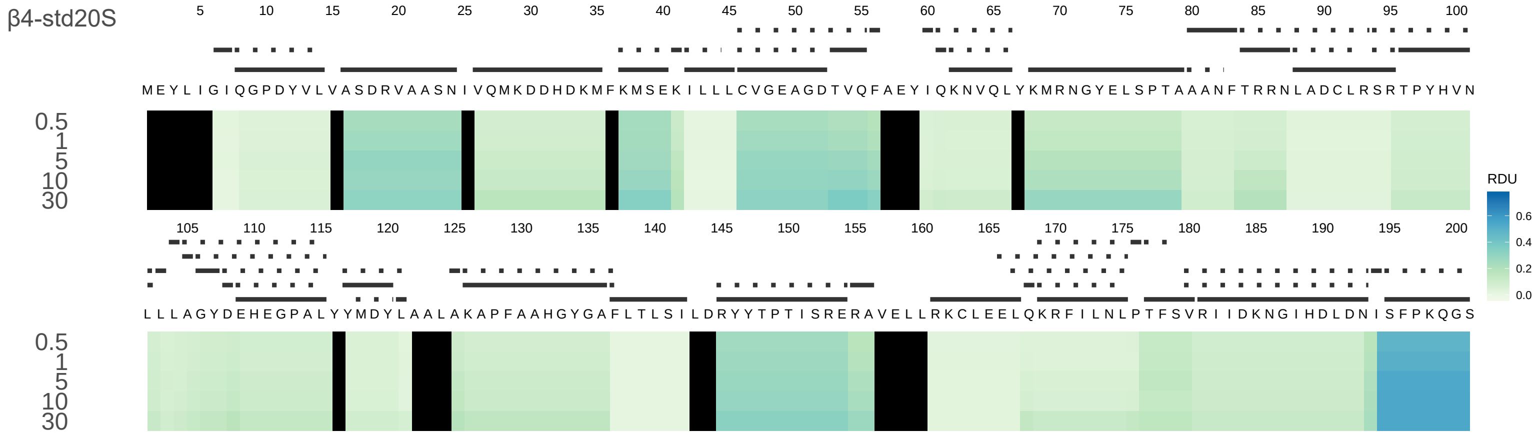
β4-std20S
MEYLIGIQGPDYVLVASDRVAASNIVQMKDDHDKMFKMSEKILLLCVGEAGDTVQFAEYIQKNVQLYKMRNGYELSPTAAANFTRRNLADCLRSRTPYHVN
LLLAGYDEHEGPALYYMDYLAALAKAPFAAHGYGAFLTLSILDRYYTPTISRERAVELLRKCLEELQKRFILNLPTFSVRIIDKNGIHDLDNISFPKQGS
0.5
1
5
10
30
RDU
0.6
0.4
0.2
0.0

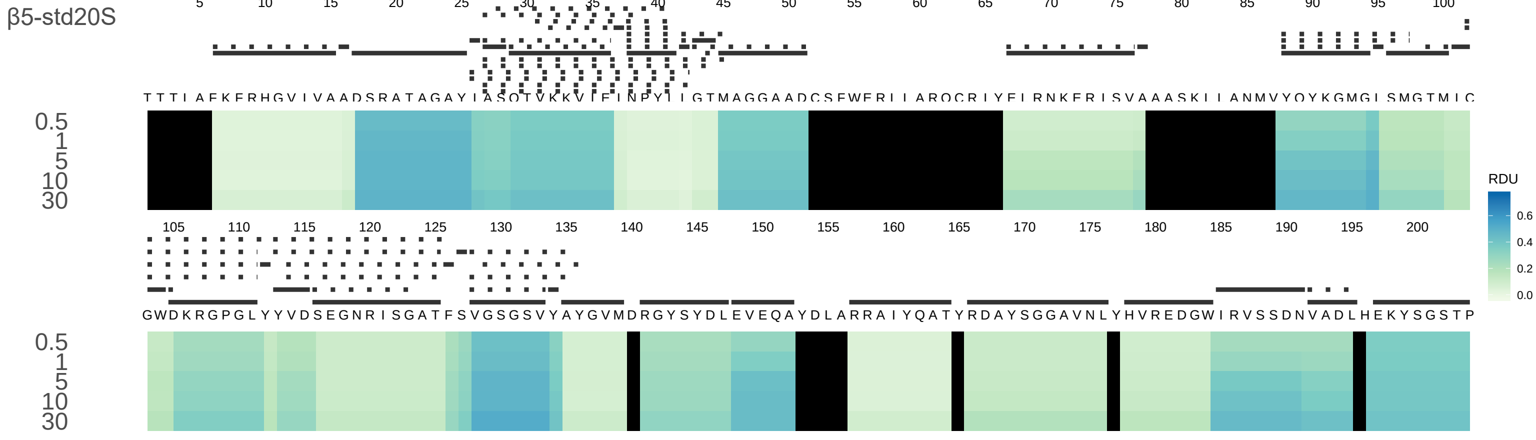
β5-std20S
0.5
1
5
10
30
105 110 115 120 125 130 135 140 145 150 155 160 165 170 175 180 185 190 195 200
GWDKRGPGLYYVDSEGNRISGATFSVGSGSVYAYGVMDRGYSYDLEVEQAYDLARRAIYQATYRDAYSGGAVNLYHVREDGWIRVSSDNVADLHEKYSGSTP
0.5
1
5
10
30
RDU
0.6
0.4
0.2
0.0

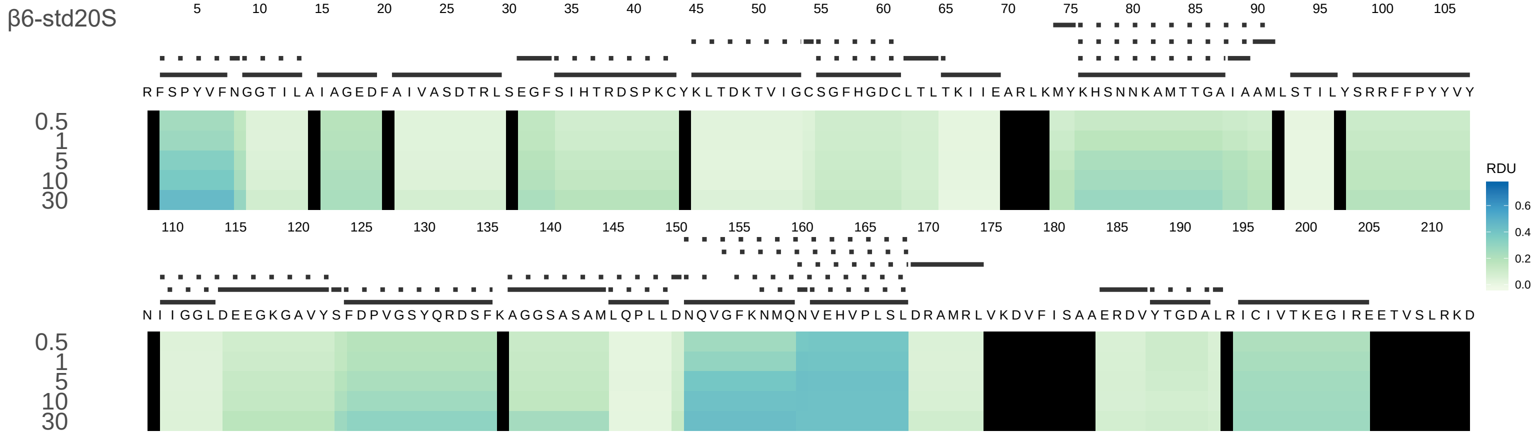
β6-std20S
RDU
0.5
1
5
10
30

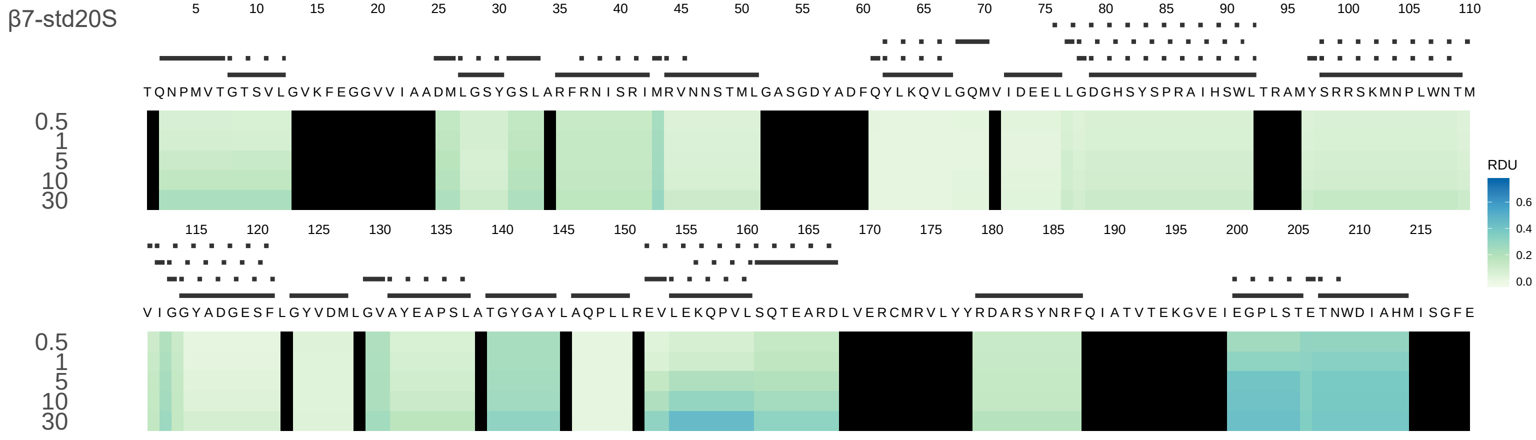
β7-std20S
TQNPMVTGTSVLGVKFEGGVVIAADMLGSYGSLARFRNISRIMRVNNSTMLGASGDYADFQYLKQVLGQMVIDEELLGDGHSYSPRAIHSWLTRAMYSRRSKMNPLWNTM
VIGGYADGESFLGYVDMLGVAYEAPSLATGYGAYLAQPLLREVLEKQPVLSQTEARDLVERCMRVLYYRDARSYNRFQIATVTEKGVEIEGPLSTETNWDIAHMISGFE
0.5
1
5
10
30
RDU
0.6
0.4
0.2
0.0

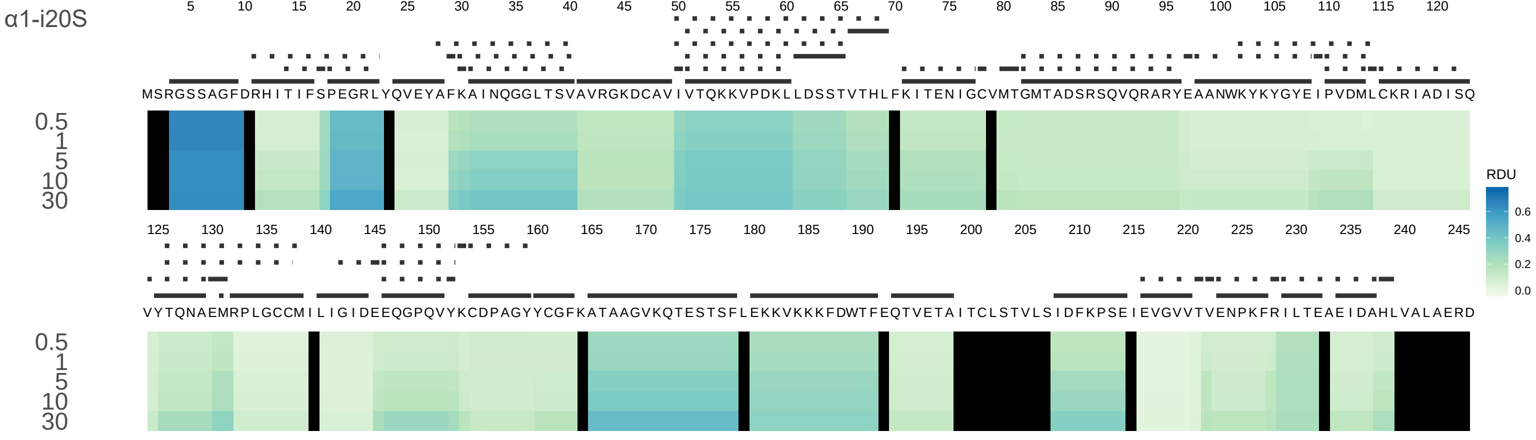
α1-i20S
MSRGSSAGFDRHITIFSPEGRLYQVEYAFKAINQGGLTSVAVRGKDCAVIVTQKKVPDKLLDSSTVTHLFKITENIGCVMTGMTADSRSQVQRARYEAANWKYKYGYEIPVDMLCKRIADISQ
VYTQNAEMRPLGCCMILIGIDEEQGPQVYKCDPAGYYCGFKATAAGVKQTESTSFLEKKVKKKFDWTFEQTVETAITCLSTVLSIDFKPSEIEVGVVTVENPKFRILTEAEIDAHLVALAERD
0.5
1
5
10
30
RDU
0.6
0.4
0.2
0.0

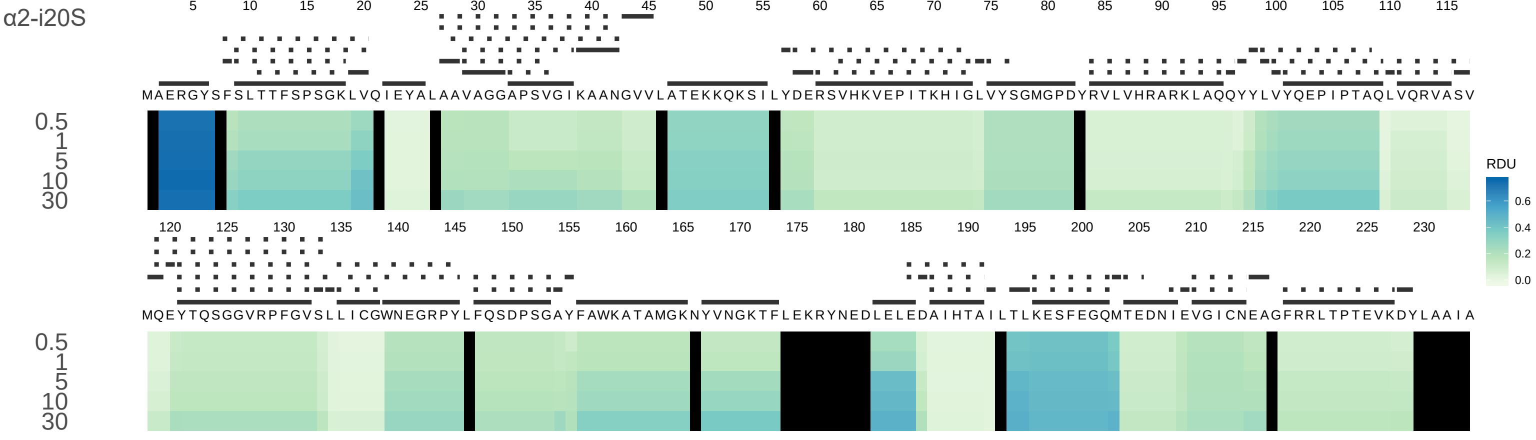
α2-i20S
5 10 15 20 25 30 35 40 45 50 55 60 65 70 75 80 85 90 95 100 105 110 115
M A E R G Y S F S L T T F S P S G K L V Q I E Y A L A A V A G G A P S V G I K A A N G V V L A T E K K Q K S I L Y D E R S V H K V E P I T K H I G L V Y S G M G P D Y R V L V H R A R K L A Q Q Y Y L V Y Q E P I P T A Q L V Q R V A S V
0.5
1
5
10
30
120 125 130 135 140 145 150 155 160 165 170 175 180 185 190 195 200 205 210 215 220 225 230
M Q E Y T Q S G G V R P F G V S L L I C G W N E G R P Y L F Q S D P S G A Y F A W K A T A M G K N Y V N G K T F L E K R Y N E D L E L E D A I H T A I L T L K E S F E G Q M T E D N I E V G I C N E A G F R R L T P T E V K D Y L A A I A
0.5
1
5
10
30
RDU
0.6
0.4
0.2
0.0

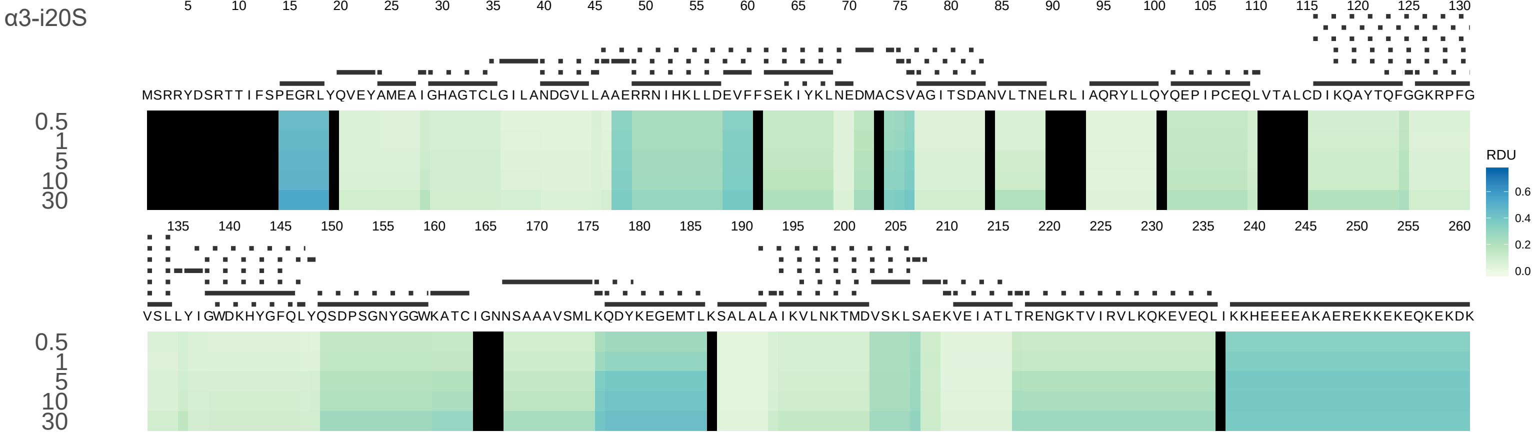
α3-i20S
MSRRYDSRTTIFSPEGRLYQVEYAMEAIGHAGTCLGILANDGVLLAAERRNIHKLLDEVFFSEKIYKLNEDMACSVAGITSDANVLTNELRLIAQRYLLQYQEPIPCEQLVTALCDIKQAYTQFGGKRPFG
VSLLYIGWDKHYGFQLYQSDPSGNYGGWKATCIGNNSAAAVSMLKQDYKEGEMTLKSALALAIKVLNKTMDVSKLSAEKVEIATLTRENGKTVIRVLKQKEVEQLIKKHEEEEAKAEREKKEKEQKEKDK
0.5
1
5
10
30
RDU

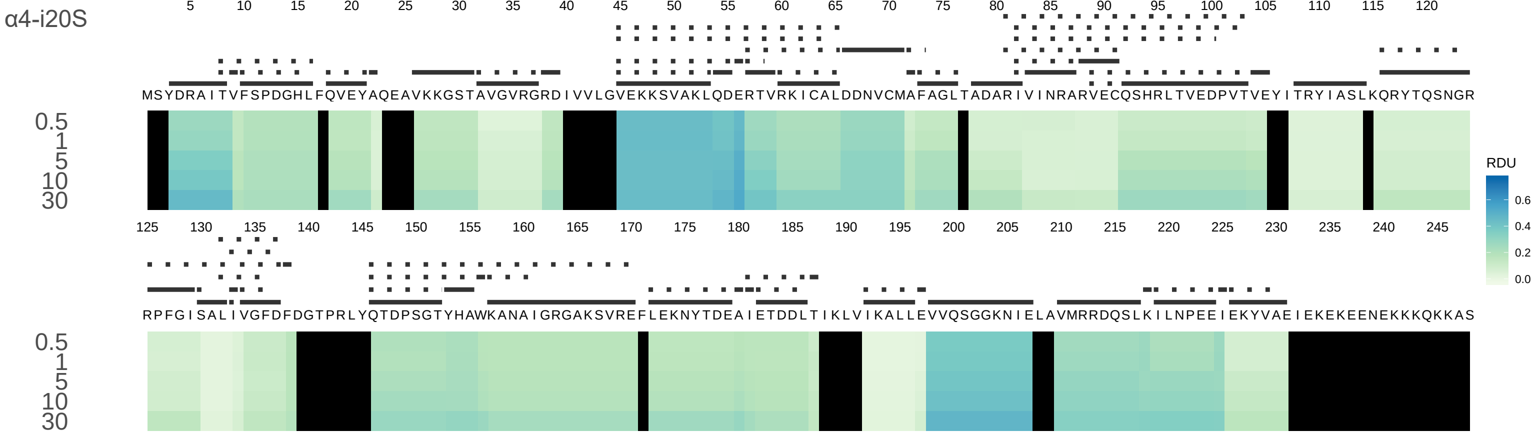
α4-i20S
5 10 15 20 25 30 35 40 45 50 55 60 65 70 75 80 85 90 95 100 105 110 115 120
MSYDRAITVFSPDGHLFQVEYAQEAVKKGSTAVGVRGRDIVVLGVEKKSVAKLQDERTVRKICALDDNVCMAFAGLTADARIVINRARVECQSHRLTVEDPVTVEYITRYIASLKQRYTQSNGR
0.5
1
5
10
30
125 130 135 140 145 150 155 160 165 170 175 180 185 190 195 200 205 210 215 220 225 230 235 240 245
RPFGISALIVGFDFDGTPRLYQTDPSGTYHAWKANAIGRGAKSVREFLEKNYTDEAIETDDLTIKLVIKALLEVVQSGGKNIELAVMRRDQSLKILNPEEIEKYVAEIEKEKEENEKKKQKKAS
0.5
1
5
10
30
RDU
0.6
0.4
0.2
0.0

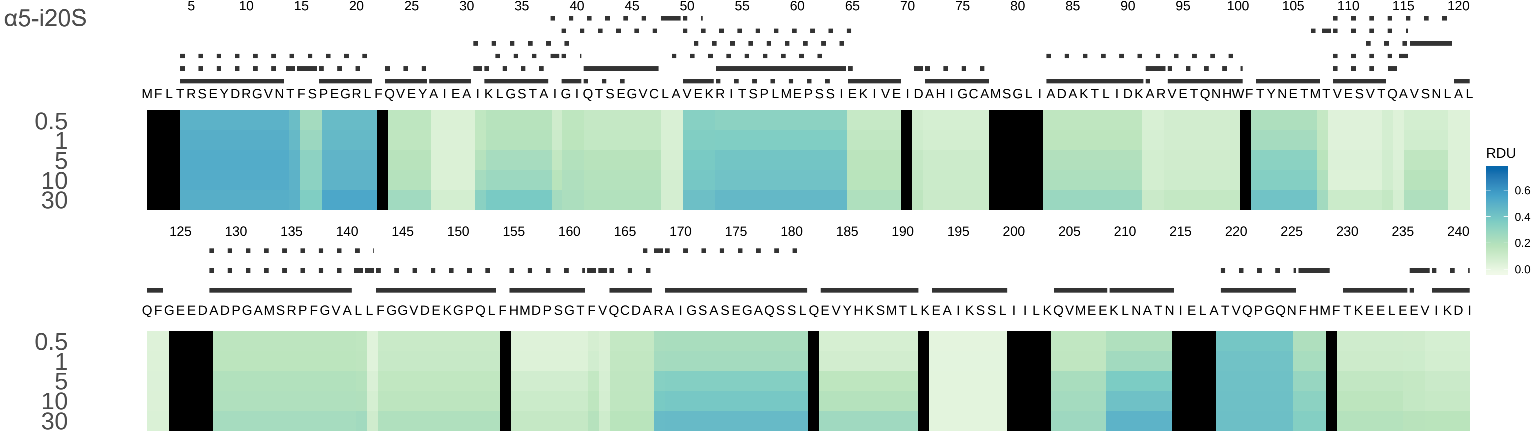
α5-i20S
5 10 15 20 25 30 35 40 45 50 55 60 65 70 75 80 85 90 95 100 105 110 115 120
MFLTRSEYDRGVNTFSPEGRLFQVEYAIEAIKLGSTAIGIQTSEGVCLAVEKRITSPLMEPSSIEKIVEIDAHIGCAMSGLIADAKTLIDKARVETQNHWFTYNETMTVESVTQAVSNLAL
0.5
1
5
10
30
125 130 135 140 145 150 155 160 165 170 175 180 185 190 195 200 205 210 215 220 225 230 235 240
QFGEEDADPGAMSRPFGVALLFGGVDEKGPQLFHMDPSGTFVQCDARAIGSASEGAQSSLQEVYHKSMTLKEAIKSSLIIILKQVMEEKLNATNIELATVQPGQNFHMFTKEELEEVIKDI
0.5
1
5
10
30
RDU
0.6
0.4
0.2
0.0

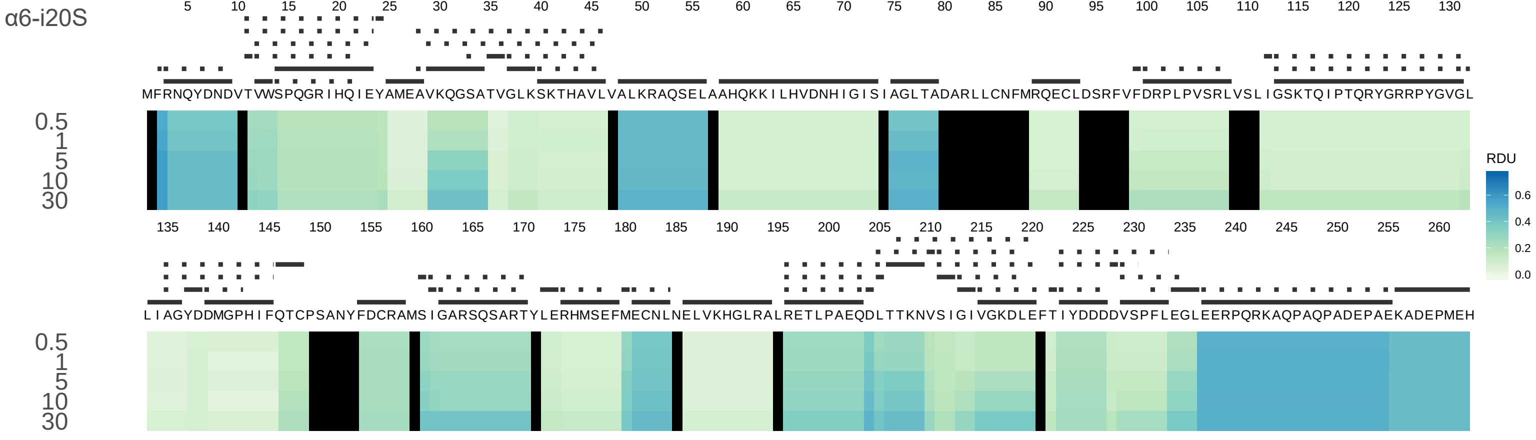
α6-i20S
MFRNQYDNDVTVWSPQGRIHQIEYAMEAVKQGSATVGLKSKTHAVLVALKRAQSELAAHQKKILHVDNHIGISIAGLTADARLLCNFMRQECLDSRFVFDRPLPVSRLVSLIGSKTQIPTQRYGRRPYGVGL
LIAGYDDMGPHIFQTCPSANYFDCRAMSIGARSQSARTYLERHMSEFMECNLNELVKHGLRALRETLPAEQDLTTKNVSIGIVGKDLEFTIYDDDDVSPFLEGLEERPQRKAQPAQPADEPAEKADEPMEH
0.5
1
5
10
30
RDU
0.6
0.4
0.2
0.0

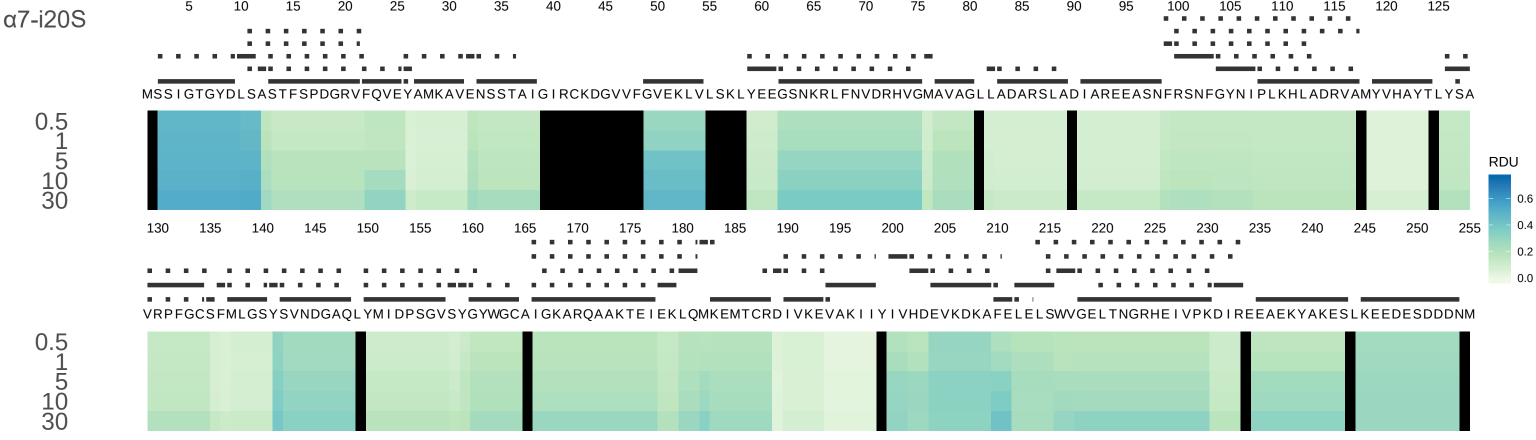
α7-i20S
MSSIGTGYDLSASTFSPDGRVFQVEYAMKAVENSSTAIGIRCKDGVVFGVEKLVLSKLYEEGSNKRLFNVDRHVGMAVAGLLADARSLADIAREEASNFRSNFGYNIPLKHLADRVAMYVHAYTLYSA
VRPFGCSFMLGSYSVNDGAQLYMIDPSGVSYGYWGCAIGKARQAAKTEIEKLQMKEMTCRDIVKEVAKIIYIVHDEVKDKAFELELSWVGELTNGRHEIVPKDIREEAEKYAKESLKEEDESDDDNM
0.5
1
5
10
30
RDU

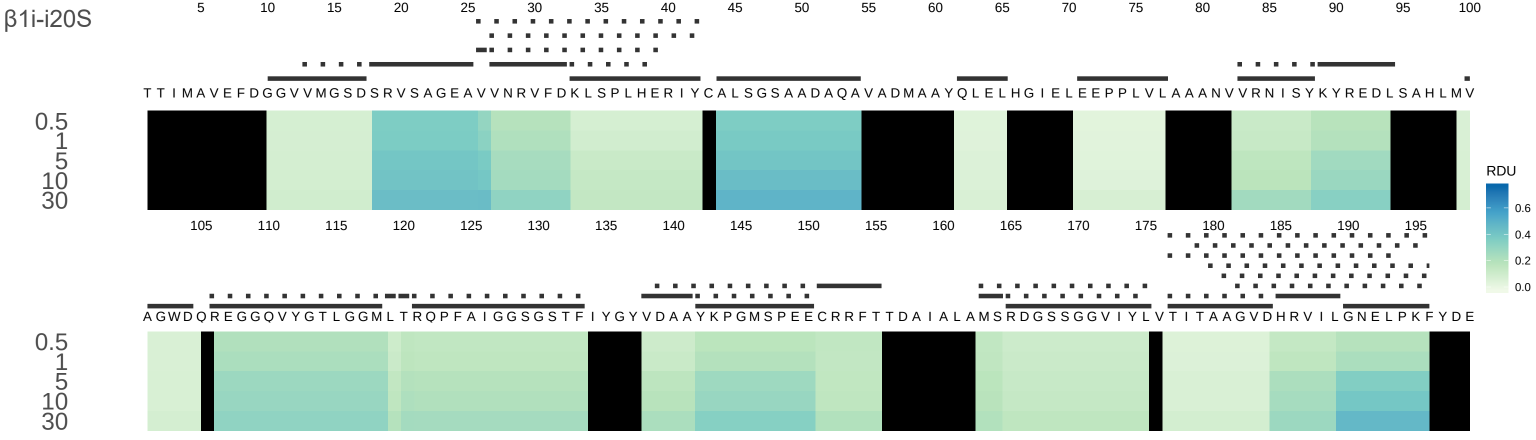
β1i-i20S
TTIMAVEFDGGVVMGSDSRVSAGEAVVNRVFDKLSPLHERIYCALSGSAADAQAVADMAAYQLELHGIELEEPPLVLAAANVVRNISYKYREDLSAHLMV
AGWDQREGGQVYGTLGGMLTRQPFAIGGSGSTFIYGYVDAAYKPGMSPEECRRFTTDAIALAMSRDGSSGGVIYLVTITAAGVDHRVILGNELPKFYDE
0.5
1
5
10
30
RDU
0.6
0.4
0.2
0.0

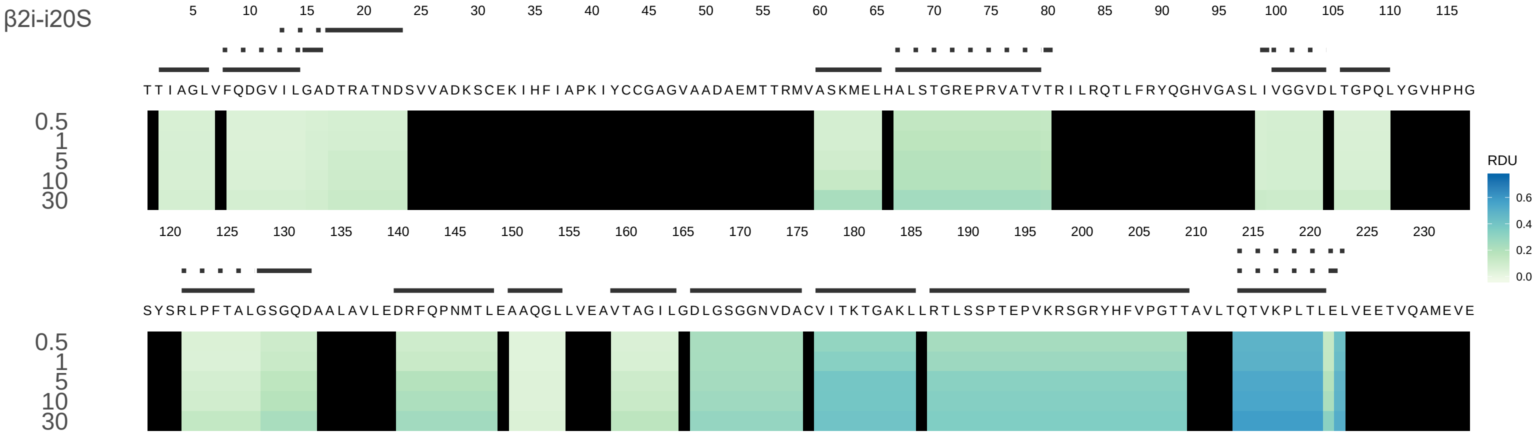
β2i-i20S
5 10 15 20 25 30 35 40 45 50 55 60 65 70 75 80 85 90 95 100 105 110 115
TTIAGLVFQDGVILGADTRATNDSVVADKSCEKIHFIAPKIYCCGAGVAADAEMTTRMVASKMELHALSTGREPRVATVTRILRQTLFRYQGHVGASLIVGGVDLTGPQLYGVHPHG
0.5
1
5
10
30
120 125 130 135 140 145 150 155 160 165 170 175 180 185 190 195 200 205 210 215 220 225 230
SYSRLPFTALGSGQDAALAVLEDRFQPNMTLEAAQGLLVEAVTAGILGDLGSGGNVDACVITKTGAKLLRTLSSPTEPVKRSGRYHFVPGTTAVLTQTVKPLTLELVEETVQAMEVE
0.5
1
5
10
30
RDU
0.6
0.4
0.2
0.0

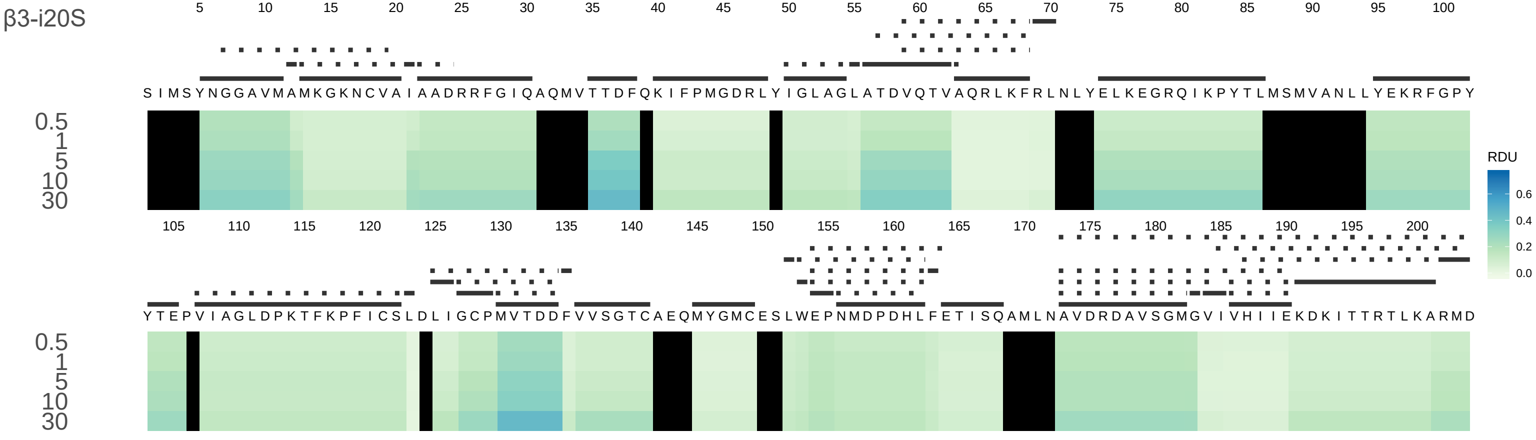
β3-i20S
5 10 15 20 25 30 35 40 45 50 55 60 65 70 75 80 85 90 95 100
S I M S Y N G G A V M A M K G K N C V A I A A D R R F G I Q A Q M V T T D F Q K I F P M G D R L Y I G L A G L A T D V Q T V A Q R L K F R L N L Y E L K E G R Q I K P Y T L M S M V A N L L Y E K R F G P Y
0.5
1
5
10
30
105 110 115 120 125 130 135 140 145 150 155 160 165 170 175 180 185 190 195 200
Y T E P V I A G L D P K T F K P F I C S L D L I G C P M V T D D F V V S G T C A E Q M Y G M C E S L W E P N M D P D H L F E T I S Q A M L N A V D R D A V S G M G V I V H I I E K D K I T T R T L K A R M D
0.5
1
5
10
30
RDU
0.6
0.4
0.2
0.0

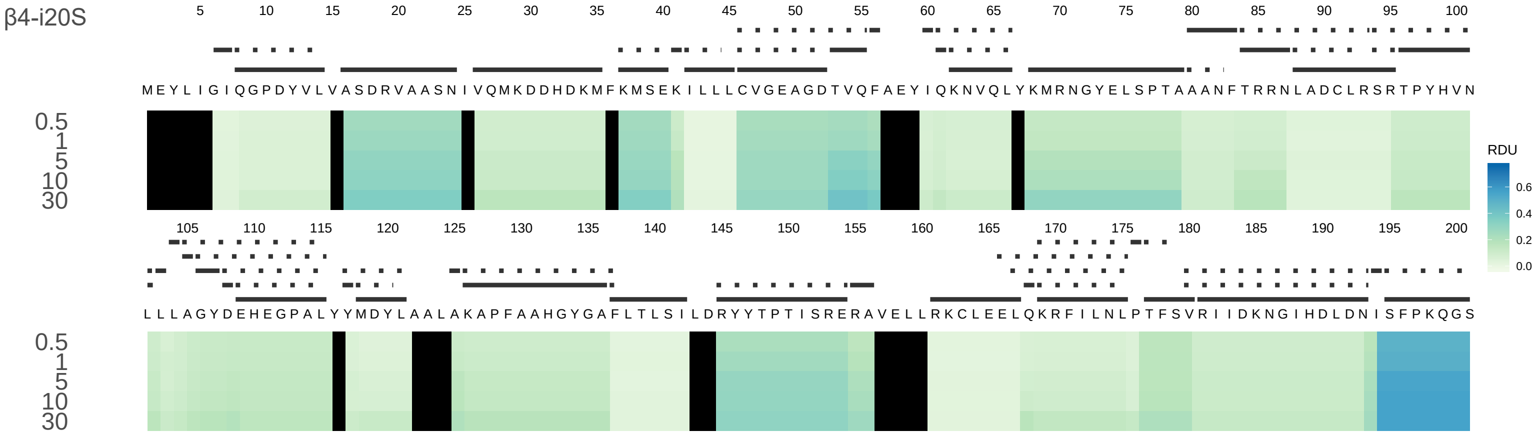

β4-i20S
5 10 15 20 25 30 35 40 45 50 55 60 65 70 75 80 85 90 95 100
M E Y L I G I Q G P D Y V L V A S D R V A A S N I V Q M K D D H D K M F K M S E K I L L L C V G E A G D T V Q F A E Y I Q K N V Q L Y K M R N G Y E L S P T A A A N F T R R N L A D C L R S R T P Y H V N
0.5
1
5
10
30
105 110 115 120 125 130 135 140 145 150 155 160 165 170 175 180 185 190 195 200
L L L A G Y D E H E G P A L Y Y M D Y L A A L A K A P F A A H G Y G A F L T L S I L D R Y Y T P T I S R E R A V E L L R K C L E E L Q K R F I L N L P T F S V R I I D K N G I H D L D N I S F P K Q G S
0.5
1
5
10
30
RDU
0.6
0.4
0.2
0.0

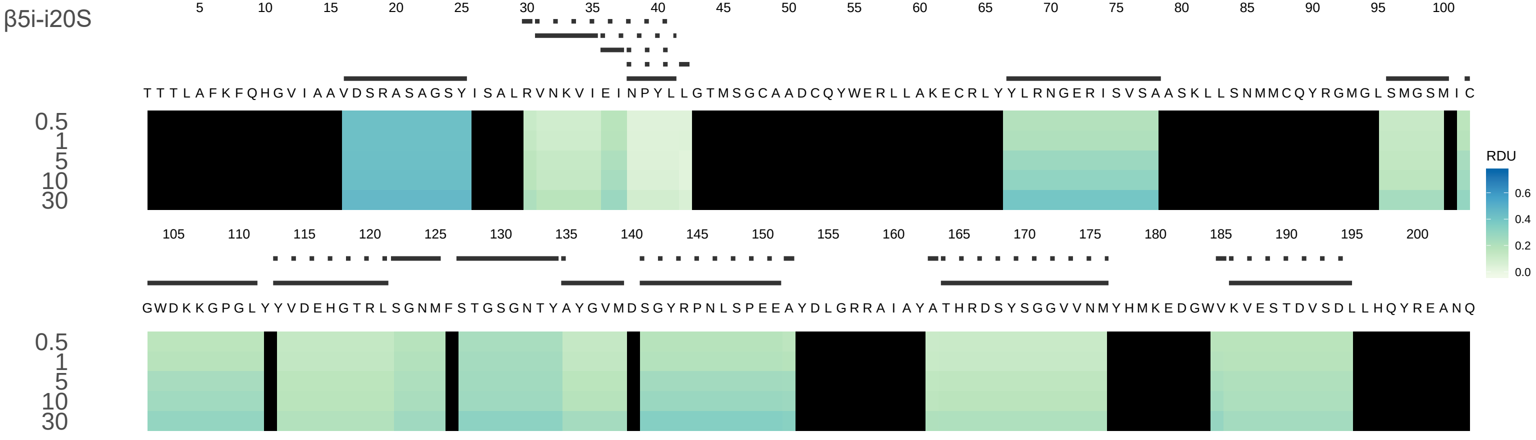
β5i-i20S
TTTLAFKFQHGVIAAVDSRASAGSYISALRVNKVIEINPYLLGTMSGCAADCQYWERLLAKECRLYYLRNGERISVSAASKLLSNMMCQYRGMGLSMGSMIC
GWDKKGPGLYYVDEHGTRLSGNMFSTGSGNTYAYGVMDSGYRPNLSPEEAYDLGRRAIAYATHRDSYSGGVVNMYHMKEDGWVKVESTDVSDLLHQYREANQ
0.5
1
5
10
30
RDU
0.6
0.4
0.2
0.0

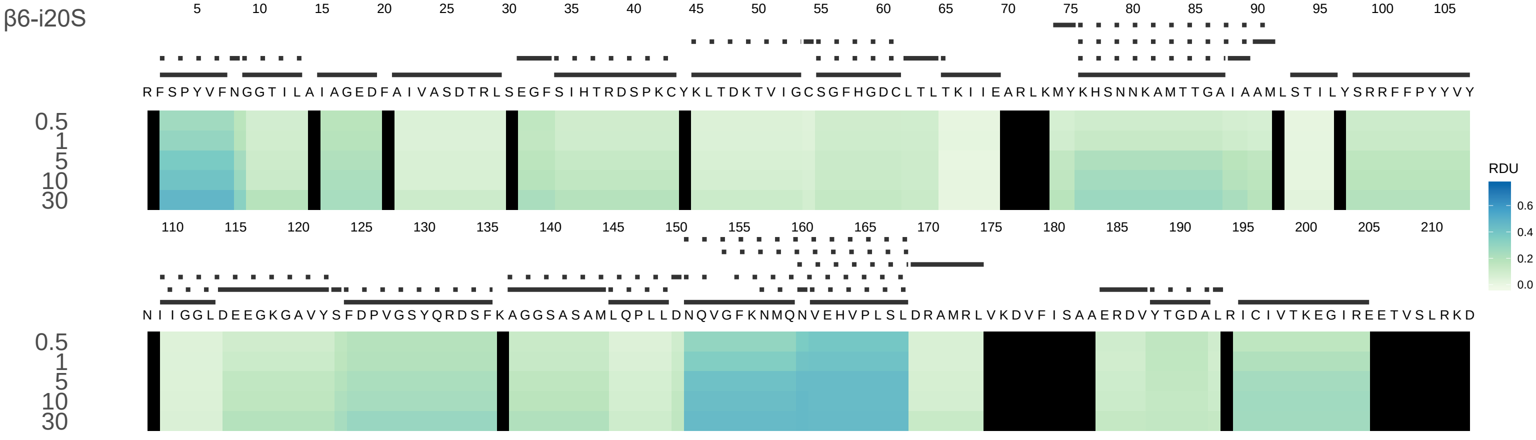

β6-i20S
RFSPYVFNGGTILAIAGEDFAIVASDTRLSEGFSIHTRDSPKCYKLTDKTVIGCSGFHGDCLTLTKIIEARLKMYKHSNNKAMTTGAIAAMLSTILYSRRFFPYYVY
NIIGGLDEEGKGAVYSFDPVGSYQRDSFKAGGSASAMLQPLLDNQVGFKNMQNVEHVPLSLDRAMRLVKDVFISAAERDVYTGDALRICIVTKEGIREETVSLRKD
0.5
1
5
10
30
RDU
0.6
0.4
0.2
0.0

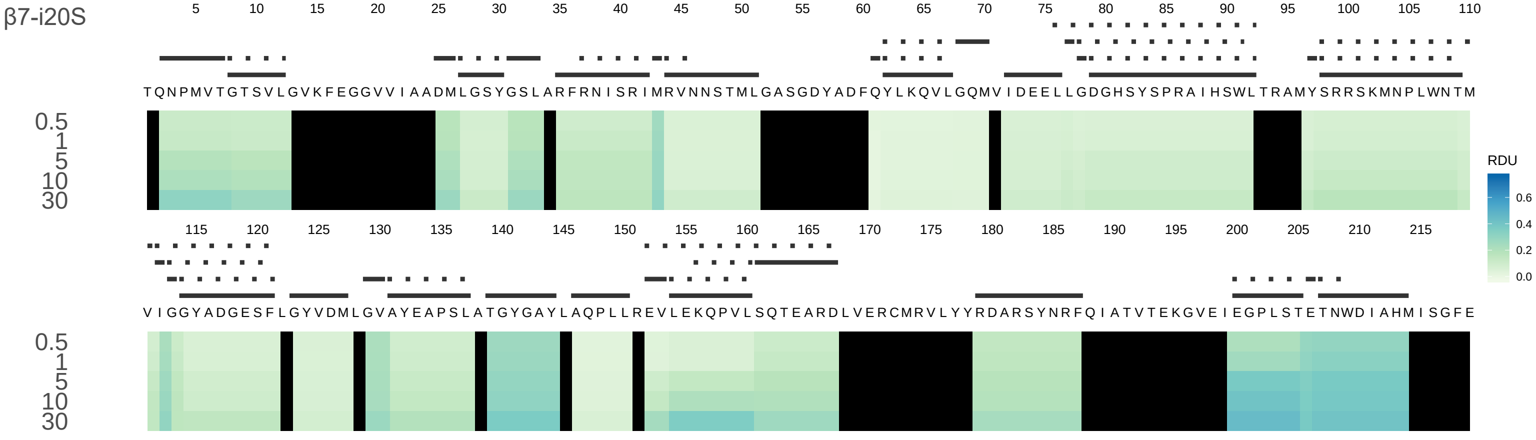

β7-i20S
5 10 15 20 25 30 35 40 45 50 55 60 65 70 75 80 85 90 95 100 105 110
TQNPMVTGTSVLGVKFEGGVVIAADMLGSYGSLARFRNISRIMRVNNSTMLGASGDYADFQYLKQVLGQMVIDEELLGDGHSYSPRAIHSWLTRAMYSRRSKMNPLWNTM
0.5
1
5
10
30
115 120 125 130 135 140 145 150 155 160 165 170 175 180 185 190 195 200 205 210 215
VIGGYADGESFLGYVDMLGVAYEAPSLATGYGAYLAQPLLREVLEKQPVLSQTEARDLVERCMRVLYYRDARSYNRFQIATVTEKGVEIEGPLSTETNWDIAHMISGFE
0.5
1
5
10
30
RDU
0.6
0.4
0.2
0.0
