# Supplementary material for: Conformational maps of human 20S proteasomes reveal PA28- and immuno-dependent inter-ring crosstalks
Source: Nat Commun. 2020 Dec 1;11:6140. doi: 10.1038/s41467-020-19934-z (PMC7708635; doi:10.1038/s41467-020-19934-z)
Supplement: Supplementary file 13 — Dataset 11 [file 41467_2020_19934_MOESM13_ESM.pdf]

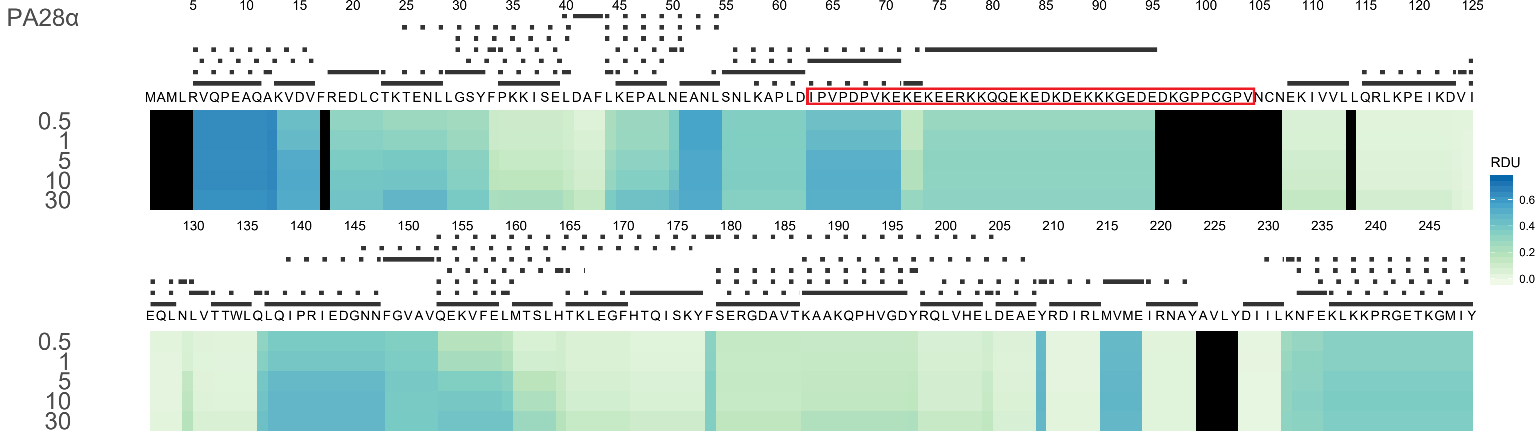

PA28α
MAMLRVQPEAQAKVDVFREDLCTKTENLLGSYFPKKISELDAFLKEPALNEANLSNLKAPLDIPVPDPVKEKEKEERKKQQEKEDKDEKKKGEDEDKGPPCGPVNCNEKIVVLLQRLKPEIKDVI
EQLNLVTTWLQLQIPRIEDGNNFGVAVQEKVFELMTSLHTKLEGFHTQISKYFSERGDAVTKAAKQPHVGDYRQLVHELDEAEYRDIRLMVMEIRNAYAVLYDIILKNFEKLKKPRGETKGMIY
0.5
1
5
10
30
RDU
0.6
0.4
0.2
0.0

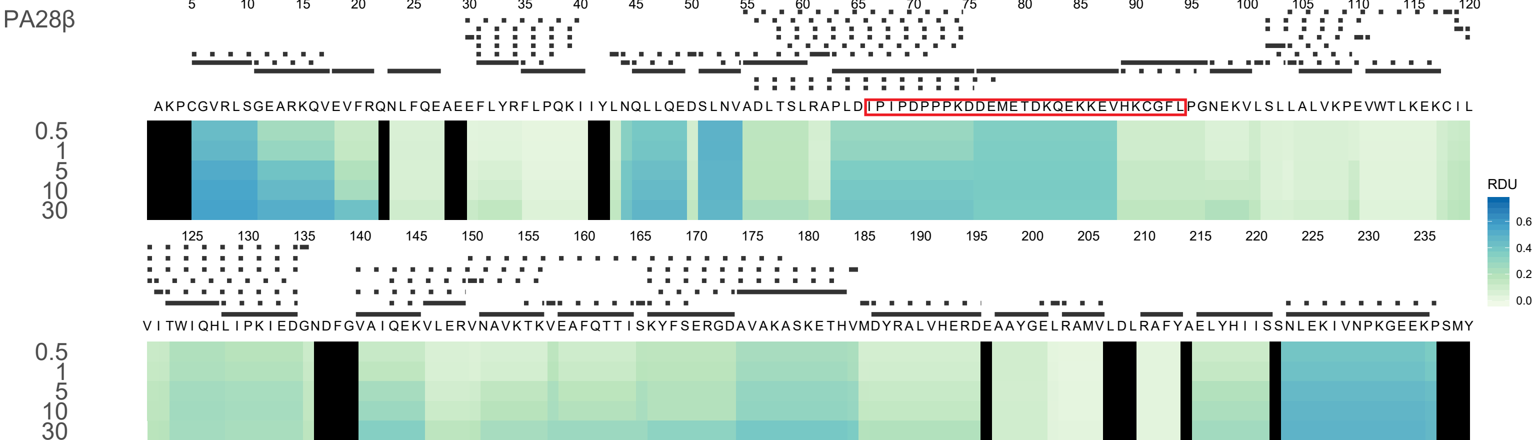
PA28β
AKPCGVRLSGEARKQVEVFRQNLFQEAEEFLYRFLPQKIIYLNQLLQEDSLNVADLTSLRAPLDIPIPDPPPKDDEMETDKQEKKEVHKCGFLPGNEKVLSLLALVKPEVWTLKEKCIL
VITWIQHLIPKIEDGNDFGVAIQEKVLERVNAVKTKVEAFQTTISKYFSERGDAVAKASKETHVMDYRALVHERDEAAYGELRAMVLDLRAFYAELYHIISSNLEKIVNPKGEEKPSMY
0.5
1
5
10
30
RDU
0.6
0.4
0.2
0.0

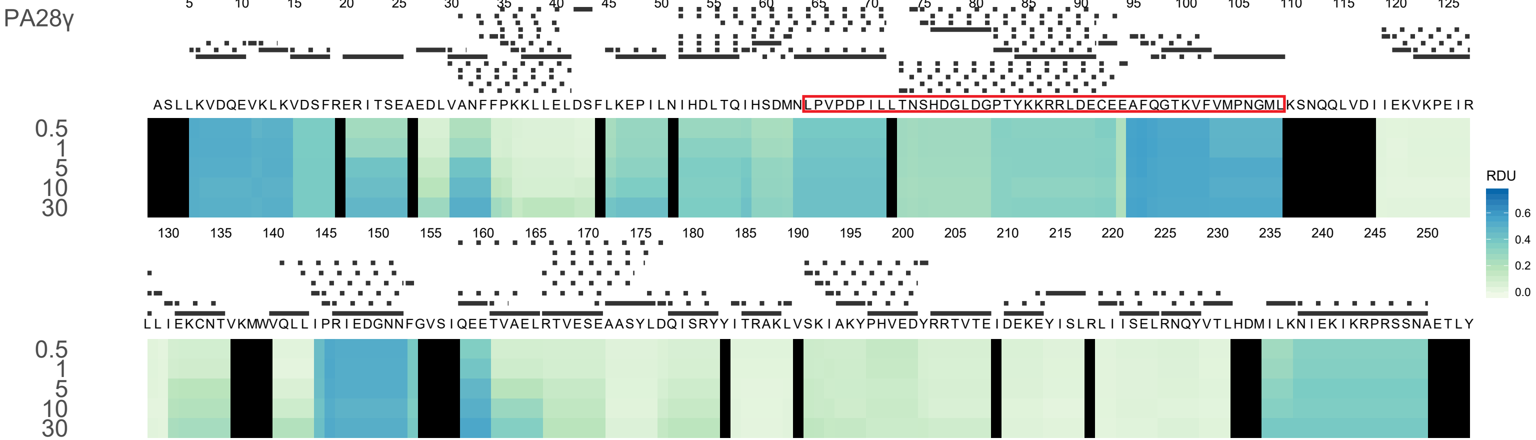

PA28γ
5 10 15 20 25 30 35 40 45 50 55 60 65 70 75 80 85 90 95 100 105 110 115 120 125
ASLLKVDQEVKLKVDSFRERITSEAEDLVANFFPKKLLELDSFLKEPILNIHDLTQIHSDMNLPVPDPILLTNSHDGLDGPTYKKRRLDECEEAFQGTKVFVMPNGMLKSNQQLVDIIEKVKPEIR
0.5
1
5
10
30
130 135 140 145 150 155 160 165 170 175 180 185 190 195 200 205 210 215 220 225 230 235 240 245 250
LLIEKCNTVKMWVQLLIPRIEDGNNFGVSIQEETVAELRTVESEAASYLDQISRYYITRAKLVSKIAKYPHVEDYRRTVTEIDEKEYISLRLIISELRNQYVTLHDMILKNIEKIKRPRSSNAETLY
0.5
1
5
10
30
RDU
0.6
0.4
0.2
0.0
