# Supplementary material for: Conformational maps of human 20S proteasomes reveal PA28- and immuno-dependent inter-ring crosstalks
Source: Nat Commun. 2020 Dec 1;11:6140. doi: 10.1038/s41467-020-19934-z (PMC7708635; doi:10.1038/s41467-020-19934-z)
Supplement: Supplementary file 15 — Dataset 13 [file 41467_2020_19934_MOESM15_ESM.pdf]

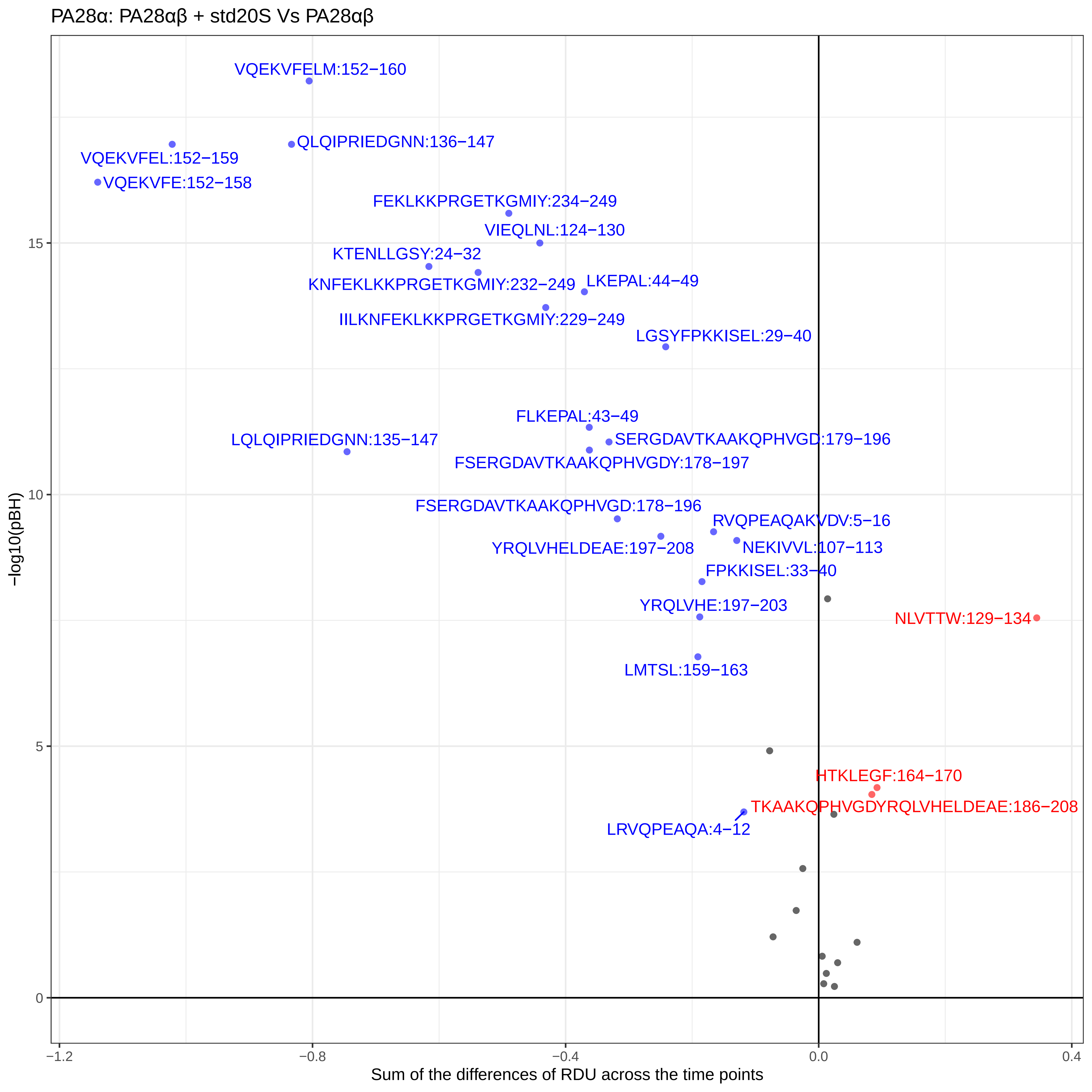

PA28α: PA28αβ + std20S Vs PA28αβ
VQEKVFELM:152−160
QLQIPRIEDGNN:136−147
VQEKVFEL:152−159
VQEKVFE:152−158
FEKLKKPRGETKGMIY:234−249
VIEQLNL:124−130
KTENLLGSY:24−32
KNFEKLKKPRGETKGMIY:232−249
LKEPAL:44−49
IILKNFEKLKKPRGETKGMIY:229−249
LGSYFPKKISEL:29−40
FLKEPAL:43−49
LQLQIPRIEDGNN:135−147
SERGDAVTKAAKQPHVGD:179−196
FSERGDAVTKAAKQPHVGDY:178−197
FSERGDAVTKAAKQPHVGD:178−196
RVQPEAQAKVDV:5−16
YRQLVHELDEAE:197−208
NEKIVVL:107−113
FPKKISEL:33−40
YRQLVHE:197−203
NLVTTW:129−134
LMTSL:159−163
HTKLEGF:164−170
TKAAKQPHVGDYRQLVHELDEAE:186−208
LRVQPEAQA:4−12
−log10(pBH)
15
10
5
0
−1.2
−0.8
−0.4
0.0
0.4
Sum of the differences of RDU across the time points

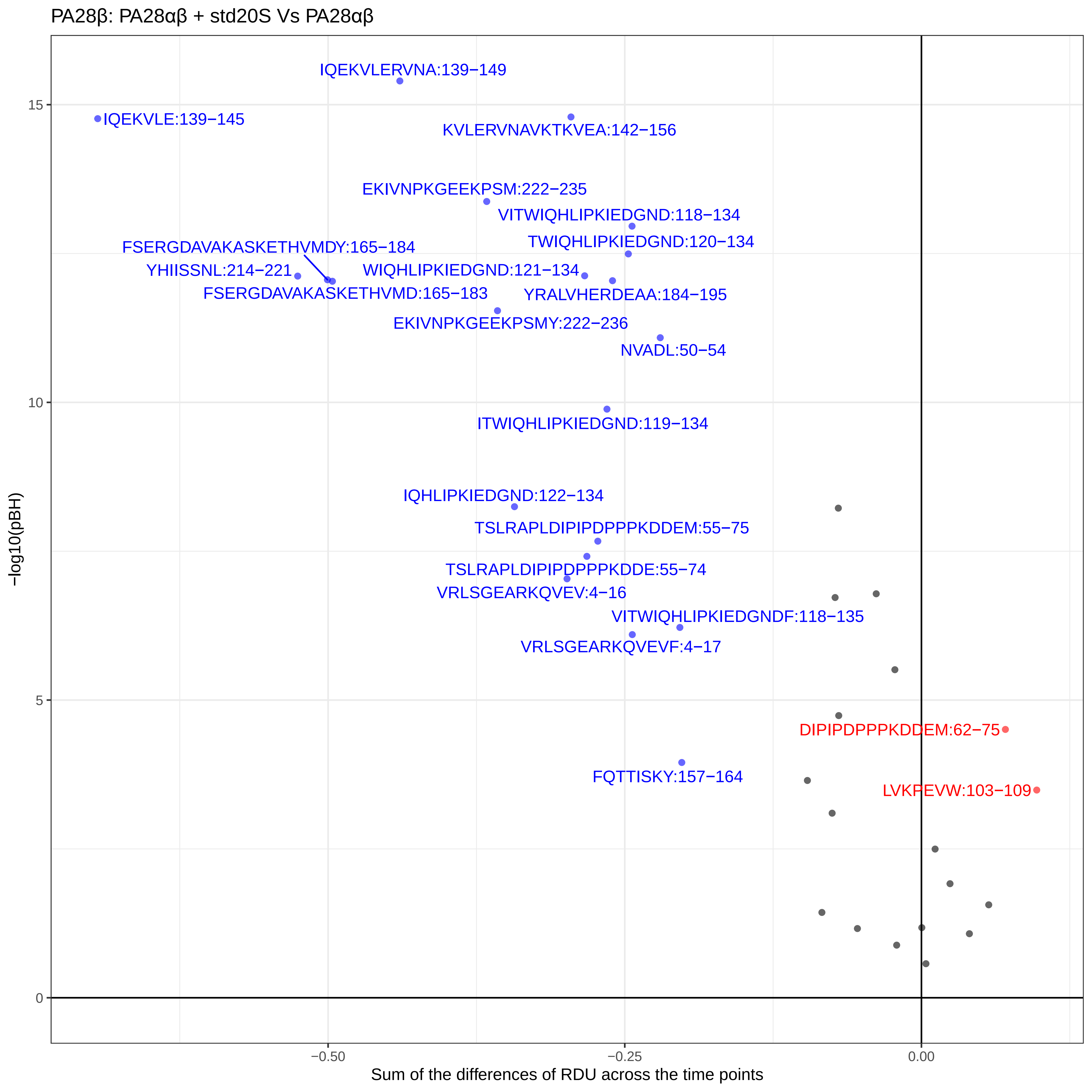

PA28β: PA28αβ + std20S Vs PA28αβ
IQEKVLERVNA:139−149
IQEKVLE:139−145
KVLERVNAVKTKVEA:142−156
EKIVNPKGEEKPSM:222−235
VITWIQHLIPKIEDGND:118−134
TWIQHLIPKIEDGND:120−134
FSERGDAVAKASKETHVMDY:165−184
YHIISSNL:214−221
WIQHLIPKIEDGND:121−134
FSERGDAVAKASKETHVMD:165−183
YRALVHERDEAA:184−195
EKIVNPKGEEKPSMY:222−236
NVADL:50−54
ITWIQHLIPKIEDGND:119−134
IQHLIPKIEDGND:122−134
TSLRAPLDIPIPDPPPKDDEM:55−75
TSLRAPLDIPIPDPPPKDDE:55−74
VRLSGEARKQVEV:4−16
VITWIQHLIPKIEDGNDF:118−135
VRLSGEARKQVEVF:4−17
DIPIPDPPPKDDEM:62−75
FQTTISKY:157−164
LVKPEVW:103−109
−log10(pBH)
15
10
5
0
−0.50
−0.25
0.00
Sum of the differences of RDU across the time points

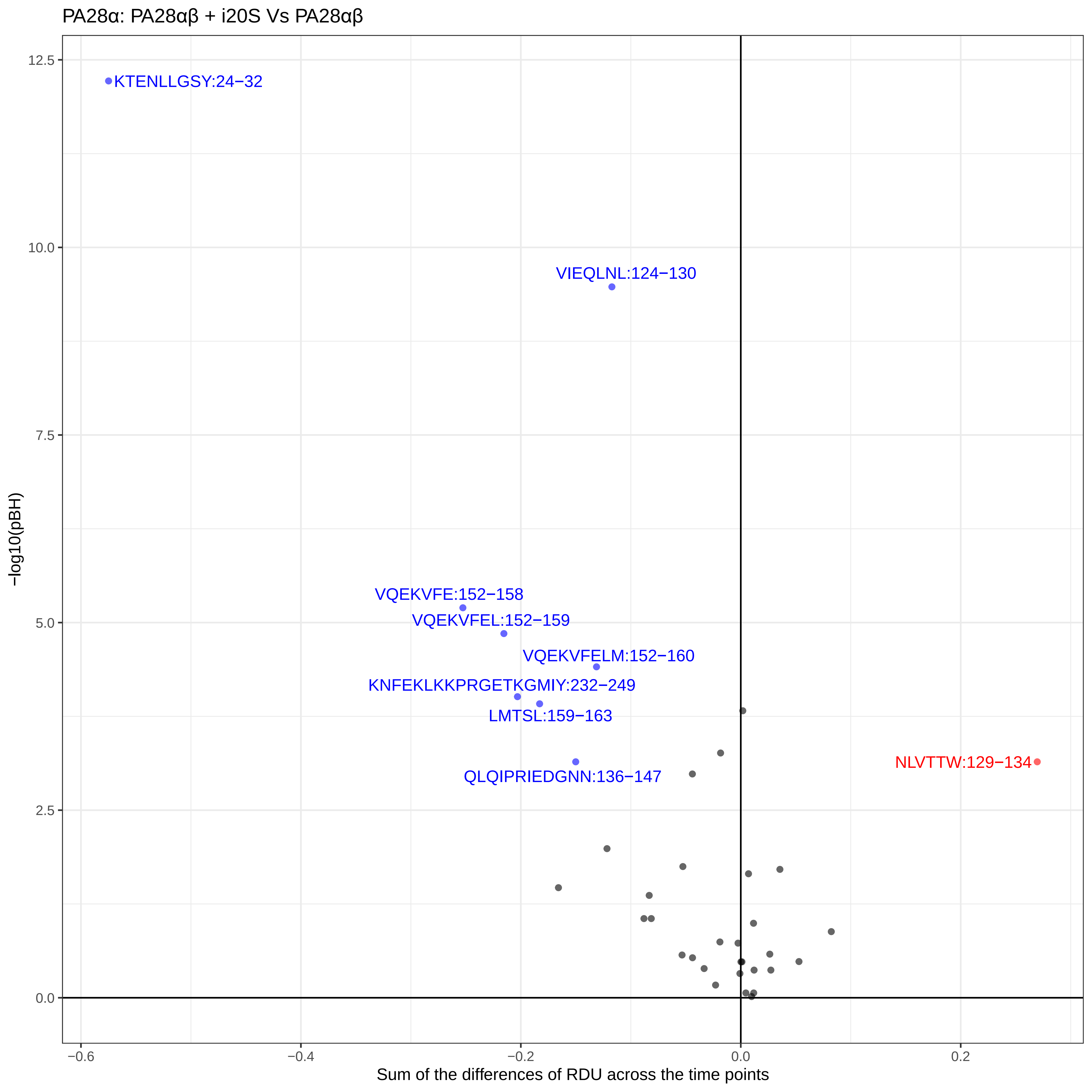

PA28α: PA28αβ + i20S Vs PA28αβ
KTENLLGSY:24−32
VIEQLNL:124−130
VQEKVFE:152−158
VQEKVFEL:152−159
VQEKVFELM:152−160
KNFEKLKKPRGETKGMIY:232−249
LMTSL:159−163
QLQIPRIEDGNN:136−147
NLVTTW:129−134
−log10(pBH)
12.5
10.0
7.5
5.0
2.5
0.0
−0.6
−0.4
−0.2
0.0
0.2
Sum of the differences of RDU across the time points

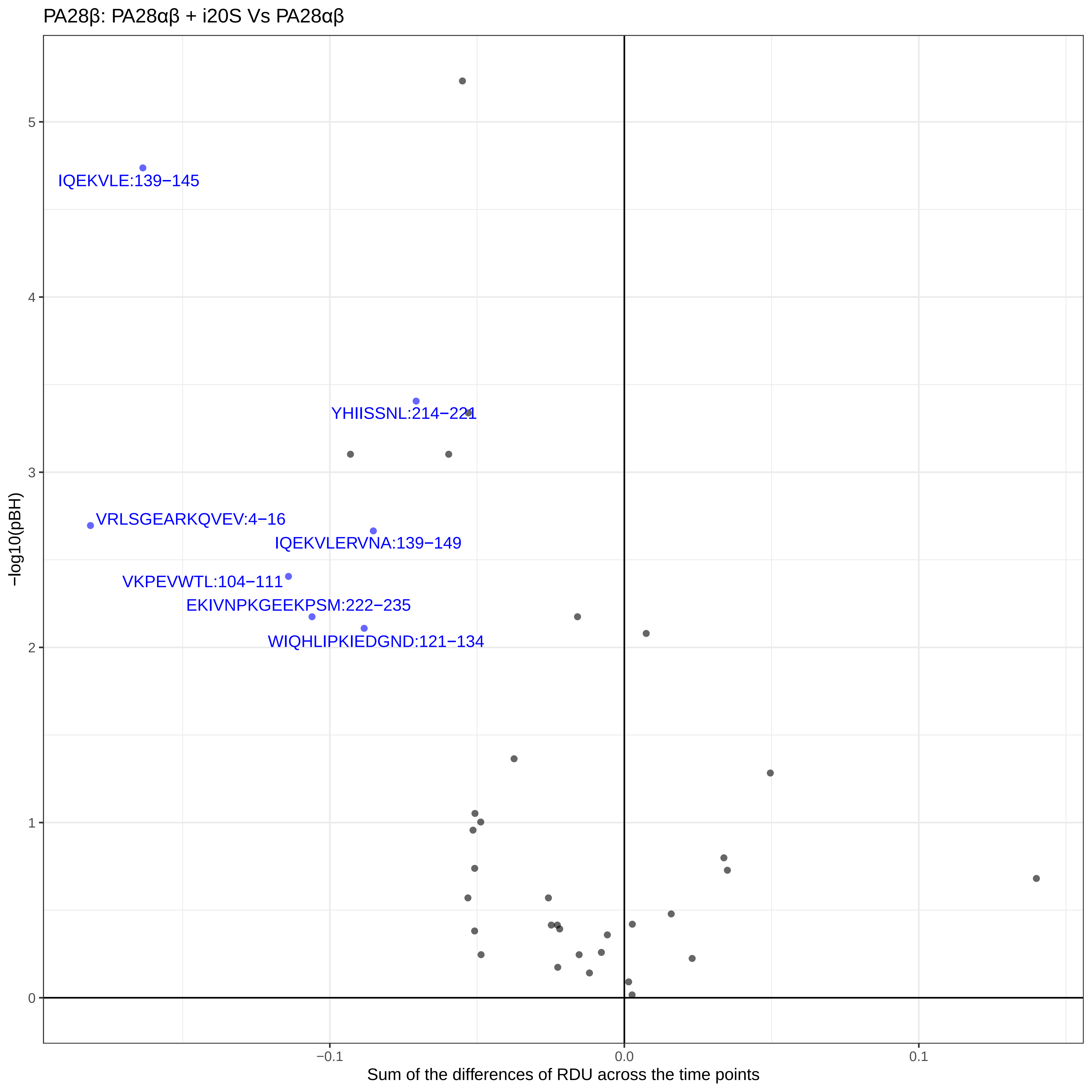
PA28β: PA28αβ + i20S Vs PA28αβ
IQEKVLE:139−145
YHIISSNL:214−221
VRLSGEARKQVEV:4−16
IQEKVLERVNA:139−149
VKPEVWTL:104−111
EKIVNPKGEEKPSM:222−235
WIQHLIPKIEDGND:121−134
−log10(pBH)
0
1
2
3
4
5
−0.1
0.0
0.1
Sum of the differences of RDU across the time points

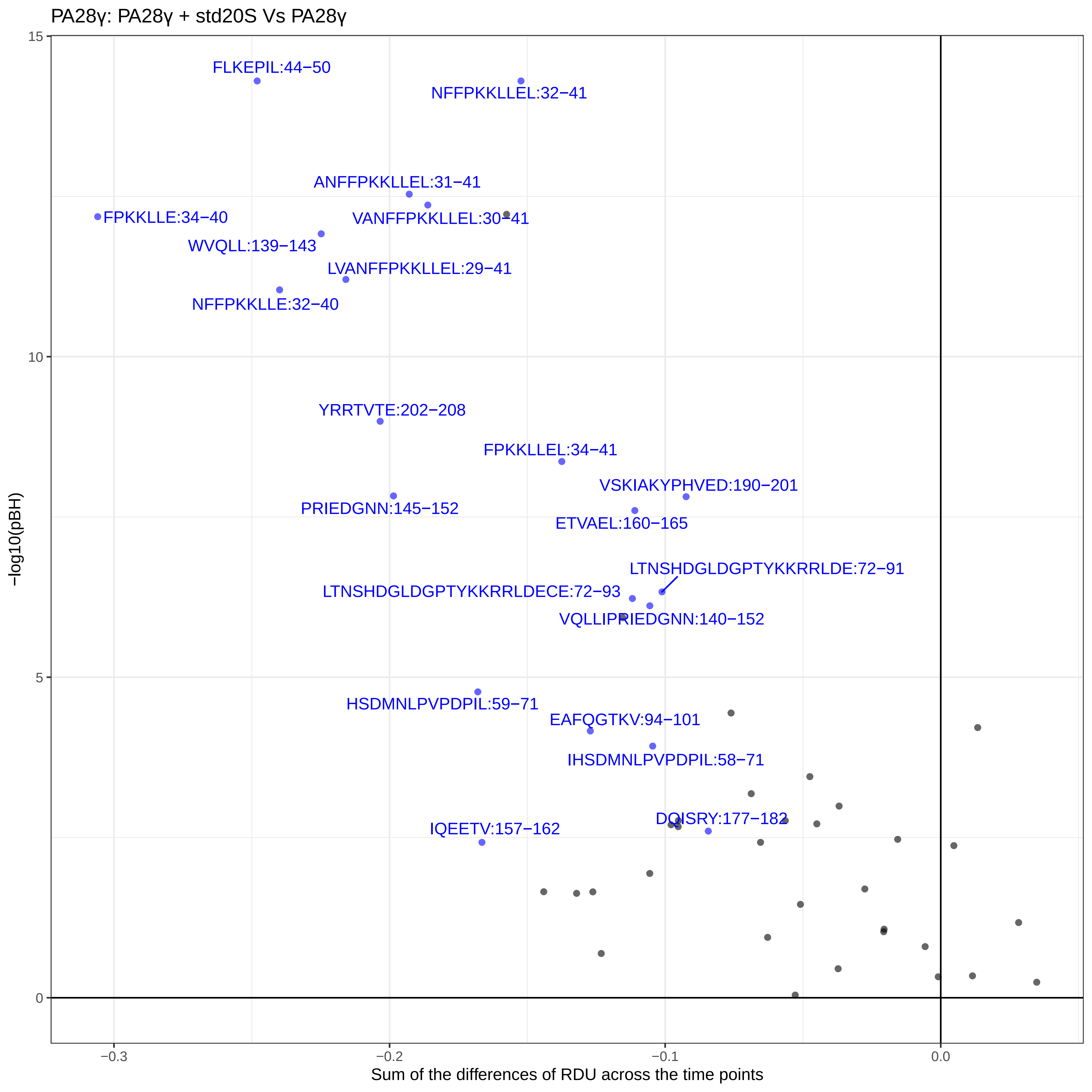

PA28γ: PA28γ + std20S Vs PA28γ
FLKEPIL:44−50
NFFPKKLLEL:32−41
ANFFPKKLLEL:31−41
FPKKLLE:34−40
VANFFPKKLLEL:30−41
WVQLL:139−143
LVANFFPKKLLEL:29−41
NFFPKKLLE:32−40
YRRTVTE:202−208
FPKKLLEL:34−41
VSKIAKYPHVED:190−201
PRIEDGNN:145−152
ETVAEL:160−165
LTNSHDGLDGPTYKKRRLDE:72−91
LTNSHDGLDGPTYKKRRLDECE:72−93
VQLLIPRIEDGNN:140−152
HSDMNLPVPDPIL:59−71
EAFQGTKV:94−101
IHSDMNLPVPDPIL:58−71
DQISRY:177−182
IQEETV:157−162
15
10
5
0
−log10(pBH)
−0.3
−0.2
−0.1
0.0
Sum of the differences of RDU across the time points

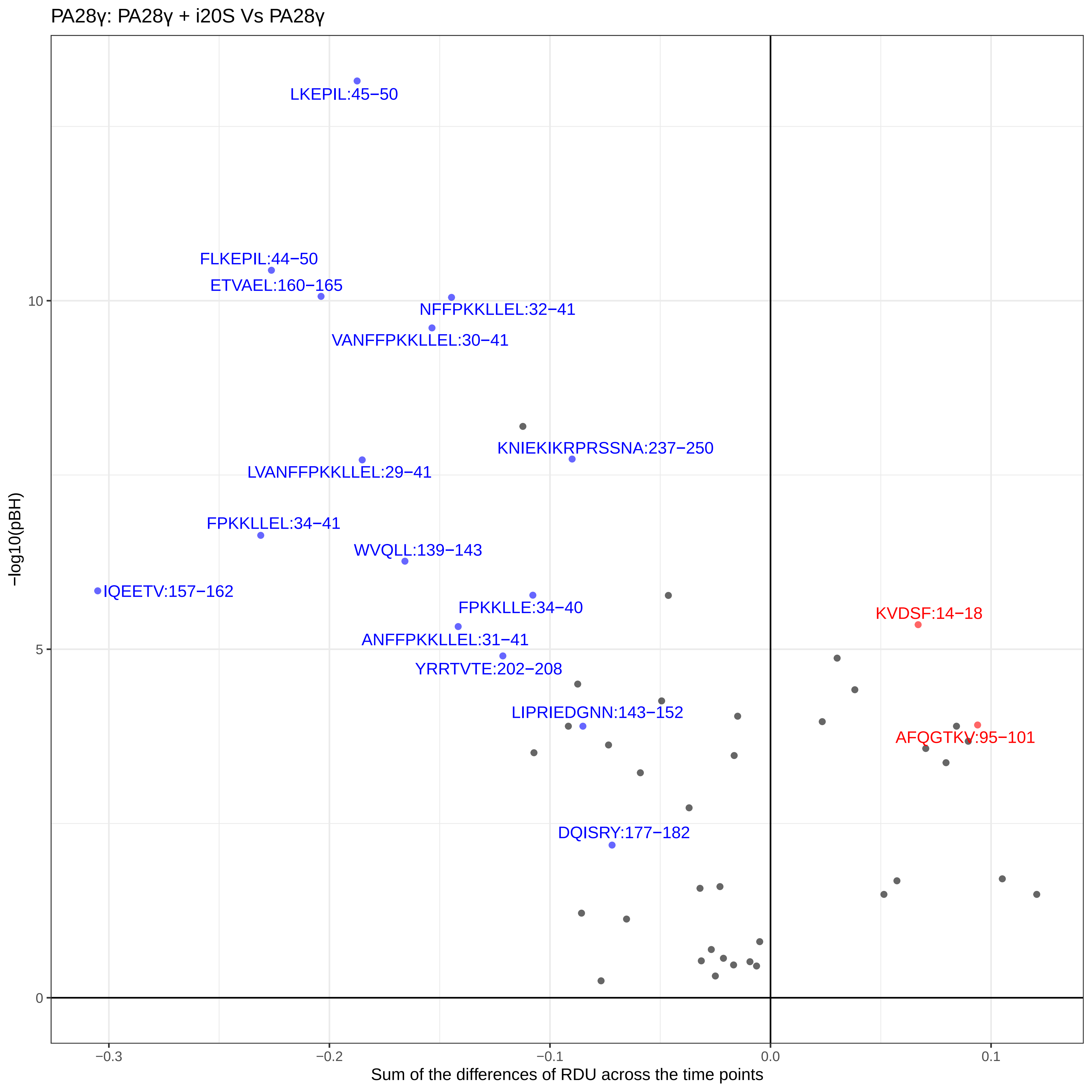
PA28γ: PA28γ + i20S Vs PA28γ
LKEPIL:45−50
FLKEPIL:44−50
ETVAEL:160−165
NFFPKKLLEL:32−41
VANFFPKKLLEL:30−41
KNIEKIKRPRSSNA:237−250
LVANFFPKKLLEL:29−41
FPKKLLEL:34−41
WVQLL:139−143
IQEETV:157−162
FPKKLLE:34−40
KVDSF:14−18
ANFFPKKLLEL:31−41
YRRTVTE:202−208
LIPRIEDGNN:143−152
AFQGTKV:95−101
DQISRY:177−182
−log10(pBH)
10
5
0
−0.3
−0.2
−0.1
0.0
0.1
Sum of the differences of RDU across the time points
