# Supplementary material for: Conformational maps of human 20S proteasomes reveal PA28- and immuno-dependent inter-ring crosstalks
Source: Nat Commun. 2020 Dec 1;11:6140. doi: 10.1038/s41467-020-19934-z (PMC7708635; doi:10.1038/s41467-020-19934-z)
Supplement: Supplementary file 19 — Reporting Summary [file 41467_2020_19934_MOESM19_ESM.pdf]

nature research

Corresponding author(s): Julien MARCOUX

Last updated by author(s): Oct 27, 2020

Reporting Summary

Nature Research wishes to improve the reproducibility of the work that we publish. This form provides structure for consistency and transparency in reporting. For further information on Nature Research policies, see our Editorial Policies and the Editorial Policy Checklist.

Statistics

For all statistical analyses, confirm that the following items are present in the figure legend, table legend, main text, or Methods section.

n/a	Confirmed	
☐	☒	The exact sample size (*n*) for each experimental group/condition, given as a discrete number and unit of measurement
☐	☒	A statement on whether measurements were taken from distinct samples or whether the same sample was measured repeatedly
☐	☒	The statistical test(s) used AND whether they are one- or two-sided<br>*Only common tests should be described solely by name; describe more complex techniques in the Methods section.*
☐	☒	A description of all covariates tested
☐	☒	A description of any assumptions or corrections, such as tests of normality and adjustment for multiple comparisons
☐	☒	A full description of the statistical parameters including central tendency (e.g. means) or other basic estimates (e.g. regression coefficient) AND variation (e.g. standard deviation) or associated estimates of uncertainty (e.g. confidence intervals)
☐	☒	For null hypothesis testing, the test statistic (e.g. *F*, *t*, *r*) with confidence intervals, effect sizes, degrees of freedom and *P* value noted<br>*Give P values as exact values whenever suitable.*
☒	☐	For Bayesian analysis, information on the choice of priors and Markov chain Monte Carlo settings
☒	☐	For hierarchical and complex designs, identification of the appropriate level for tests and full reporting of outcomes
☒	☐	Estimates of effect sizes (e.g. Cohen's *d*, Pearson's *r*), indicating how they were calculated

Our web collection on statistics for biologists contains articles on many of the points above.

Software and code

Policy information about availability of computer code

Data collection	Data was collected with MassLynX v.4.1 from Waters Scientific (Manchester, UK) and the robot was controlled via HDx Director v.1.0.3.9 (LEAP Technologies, Carborro, NC, USA).
Data analysis	Peptide identification was performed with ProteinLynx Global SERVER v.3.0.2 (PLGS, Waters, Manchester, UK). Data was analysed with DynamX v.3.0 from Waters Scientific (Manchester, UK). The R scripts of the analysis, with the fasta files and output tables and cxc files are freely available at zenodo.org with the DOI 10.5281/zenodo.3769174 under the Creative Commons Attribution 4.0 International licence. The resulting data were mapped to available 3D structures using our recently developed open-source web application HDX-Viewer v.1.2. All molecular representations were generated in UCSF ChimeraX v.0.9 (2019-06-06).

For manuscripts utilizing custom algorithms or software that are central to the research but not yet described in published literature, software must be made available to editors and reviewers. We strongly encourage code deposition in a community repository (e.g. GitHub). See the Nature Research guidelines for submitting code & software for further information.

Data

Policy information about availability of data

All manuscripts must include a data availability statement. This statement should provide the following information, where applicable:

- Accession codes, unique identifiers, or web links for publicly available datasets
- A list of figures that have associated raw data
- A description of any restrictions on data availability

The mass spectrometry proteomics data have been deposited to the ProteomeXchange Consortium via the PRIDE partner repository with the data set identifier PXD018921.

Field-specific reporting

Please select the one below that is the best fit for your research. If you are not sure, read the appropriate sections before making your selection.

☒ Life sciences ☐ Behavioural & social sciences ☐ Ecological, evolutionary & environmental sciences

For a reference copy of the document with all sections, see nature.com/documents/nr-reporting-summary-flat.pdf

Life sciences study design

All studies must disclose on these points even when the disclosure is negative.

Sample size	Each timepoint was analysed in triplicate, following the recommendations from Masson et al, Nature Methods 2019 (Recommendations for performing, interpreting and reporting hydrogen deuterium exchange mass spectrometry (HDX-MS) experiments) vol. 16 , 595–602.
Data exclusions	Raw MS spectra were inspected by specialists in the fields and spectra wrongly identified were removed from the analysis at an early stage.
Replication	All data were acquired in triplicates. The variances are reported. All attempts at replication were successful.
Randomization	not applicable to HDX-MS as randomization is not supported by the existing acquisition and data analysis software.
Blinding	not applicable to HDX-MS as blinding is not supported by the existing acquisition and data analysis software.

Reporting for specific materials, systems and methods

We require information from authors about some types of materials, experimental systems and methods used in many studies. Here, indicate whether each material, system or method listed is relevant to your study. If you are not sure if a list item applies to your research, read the appropriate section before selecting a response.

Materials & experimental systems

n/a	Involved in the study	
☒	☐	Antibodies
☒	☐	Eukaryotic cell lines
☒	☐	Palaeontology and archaeology
☒	☐	Animals and other organisms
☒	☐	Human research participants
☒	☐	Clinical data
☒	☐	Dual use research of concern

Methods

n/a	Involved in the study	
☒	☐	ChIP-seq
☒	☐	Flow cytometry
☒	☐	MRI-based neuroimaging
